# An Efficient Greener Approach for *N*-acylation of Amines in Water Using Benzotriazole Chemistry

**DOI:** 10.3390/molecules25112501

**Published:** 2020-05-28

**Authors:** Tarek S. Ibrahim, Israa A. Seliem, Siva S. Panda, Amany M. M. Al-Mahmoudy, Zakaria K. M. Abdel-Samii, Nabil A. Alhakamy, Hani Z. Asfour, Mohamed Elagawany

**Affiliations:** 1 Department of Pharmaceutical Chemistry, Faculty of Pharmacy, King Abdulaziz University, Jeddah 21589, Saudi Arabia; tmabrahem@kau.edu.sa; 2 Department of Pharmaceutical Organic Chemistry, Faculty of Pharmacy, Zagazig University, Zagazig 44519, Egypt; isliem@augusta.edu (I.A.S.); amanysinger77@gmail.com (A.M.M.A.-M.); zakariaabdelsamii@yahoo.com (Z.K.M.A.-S.); 3 Department of Chemistry & Physics, Augusta University, Augusta, GA 30912, USA; 4 Department of Pharmaceutics, Faculty of Pharmacy, King Abdulaziz University, Jeddah 21589, Saudi Arabia; nalhakamy@kau.edu.sa; 5 Department of Medical Microbiology and Parasitology, Faculty of Medicine, King Abdulaziz University, Jeddah 21589, Saudi Arabia; hasfour@kau.edu.sa; 6 Department of Pharmaceutical Chemistry, faculty of pharmacy, Damanhour University, Damanhour 22511, Egypt; alfath_tours@yahoo.com

**Keywords:** green chemistry, one-pot synthesis, acylation, benzotriazole chemistry, benzimidazole, microwave, aryl amide

## Abstract

A straightforward, mild and cost-efficient synthesis of various arylamides in water was accomplished using versatile benzotriazole chemistry. Acylation of various amines was achieved in water at room temperature as well as under microwave irradiation. The developed protocol unfolds the synthesis of amino acid aryl amides, drug conjugates and benzimidazoles. The environmentally friendly synthesis, short reaction time, simple workup, high yields, mild conditions and free of racemization are the key advantages of this protocol.

## 1. Introduction

*N*-Acylation reactions are widely used in the organic chemistry, biology, pharmaceutical and agricultural industries [1,2,3]. Chemically, they are a straightforward and powerful tool for the protection of amino groups in multistep organic syntheses, for their convenient activation towards further chemical transformations, or as widespread amide building blocks in biologically active targets, natural products and pharmaceuticals [4,5].

The amide bond is exceptionally imperative in medicinal chemistry [6,7,8]. Amide groups contribute to the unique properties of peptides, proteins, and numerous other natural and synthetic compounds. Most of the natural products and clinically used drugs contain an amide bond [9,10,11,12,13,14]. Approximately 25% of the pharmaceuticals present on the market contain at least one amide unit [15], and the functional group was present in 2/3 of the drug candidates surveyed by three leading pharmaceutical companies in 2006 [16]. A survey of the literature reveals that many drugs available in the market, such as *Penicillin* (antibacterial), *pyrazinamide* (antitubercular), *atorvastatin* (antihyperlipidemic) [17] and *valsartan* (angiotensin receptor), possess their specific capabilities due to presence of amide linkage in their structures [18].

Acylation using acetyl chloride and acetic anhydride is common among various reported strategies. However, *N*-acylation through acyl chloride and/or acid anhydride has been associated with many inherited disadvantages [19,20]. Further, for amino acid acylation, different coupling reagents are used, which are mostly nonselective, hazardous and difficult to handle [21].

To overcome the challenges of acyl chloride- and acid anhydride-mediated *N*-acylation reactions using acyl chlorides and/or acid anhydrides, numerous strategies have been investigated. Among them, the metal-catalyzed or direct coupling of unactivated carboxylic acids [22,23,24], acylation through *N*-acyl 1,5-diazabicyclo[4.3.0]non-5-ene (DBN) tetraphenylborate salts [25], Beckman rearrangements using mercury and ruthenium catalysts [26,27], copper-catalyzed oxidative amidation of aldehydes [28,29], triazole- and imidazole-mediated acyl transfer reactions [30,31,32], and acylation method through acylbenzotriazoles [33,34,35] are common. Benzotriazole chemistry has been explored, by the Katritzky group [19,21,36,37,38,39,40] and others [41,42], in various types of reactions, including in the synthesis of amides.

On the other hand, benzimidazoles are crucial core structures used to develop pharmaceuticals and materials. Substituted benzimidazoles exhibit biological activities such as antitumor [43], antihypertensive [44], antiulcer [45] and enzyme inhibition [46]. Some commonly employed synthetic methods for benzimidazoles include: (i) reaction of 1,2-phenylenediamines with carboxylic acids or their derivatives, like amidates, nitriles or orthoesters, in the presence of polyphosphoric acid [47] or mineral acids [48]; (ii) cyclization of *N*-(*N*-arylbenzimidoyl)-1,4-benzoquinoneimines under a thermal or acidic environment [49]; (iii) utilizing *o*-nitroanilines as intermediates [50]; (iv) oxidative cyclocondensation of *o*-phenylenediamine with aldehydes [51].

Recently, the development of green synthetic methods has become an important strategy in organic synthesis. Water has experienced increasing popularity due to being inexpensive, readily available, and environmentally benign. In addition, water: (i) is cheap, nonflammable, non-toxic and safe for use; (ii) eliminates additional efforts required to dry the substrates/reagents before use; (iii) offers unique physical and chemical properties that often achieve the reactivity or selectivity unattainable in organic solvents; and (iv) allows easy product isolation by filtration [52,53].

Benzotriazole chemistry has been practiced extensively in our group, and has often been found to be superior to conventional routes for acylation [21]. Earlier, we reported the acylation of mesalazine [54] and the synthesis of benzothiazole [52] in water under microwave conditions. In this communication, we extend the efficient synthetic protocol for the *N*-acylation of amines, which could be an important tool for conjugate chemistry and also for the synthesis of 2-substituted benzimidazoles without any catalyst, organic solvent or additional reagent. This protocol runs under both microwave and room temperature and gives quantitative yields. To the best of our knowledge, this is the first environmentally benign, catalyst- and organic solvent-free synthesis of *N*-acylated products of amines in water.

## 2. Results and Discussion

Carboxamines are important key intermediates, scaffolds for polymers, dendrimers and bioactive molecules [55]. Among arylamines, amino acid arylamides are often used as substrates in fluorogenic, chromogenic and amperogenic enzymatic assays [56]. For these applications, chirality is an important factor. Several methods have been reported for arylamides, including the use of enzymes and flow chemistry [57,58,59,60,61,62,63,64,65,66,67,68,69,70,71], and we believe we are reporting, for the first time, the synthesis of amino acid arylamides in water.

We investigated the reaction conditions for the *N*-acylation of anilines with our in-house prepared, protected aminoacylbenzotriazoles in water. Optimization of the reaction conditions showed the best outcomes under microwave irradiation at 50 °C for 15–20 min, over conventional heating (Table 1). We were also able to get the desired product by stirring the reactants at room temperature for 1–2 h.

There was no significant change in reaction time and yield when the above reaction was carried out both in tap water and saturated brine solution separately. Furthermore, we also tried the reaction in deionized water, to rule out the possibility of any metallic impurities from tap water catalyzing the reaction. The results obtained were comparable in tap and deionized water, so to avoid the effort and energy consumption needed to prepare deionized water, we chose tap water for our reactions.

Even though room temperature works for the *N*-acylation reactions, at a larger-scale, the reactions proceed nearly to completion as some of the reactants are left unreacted. We carried out the above reaction in both conventional heating and microwave irradiation conditions on a large scale. We got a better yield with high purity under microwave conditions, in comparison with conventional heating.

**Scheme 1:**
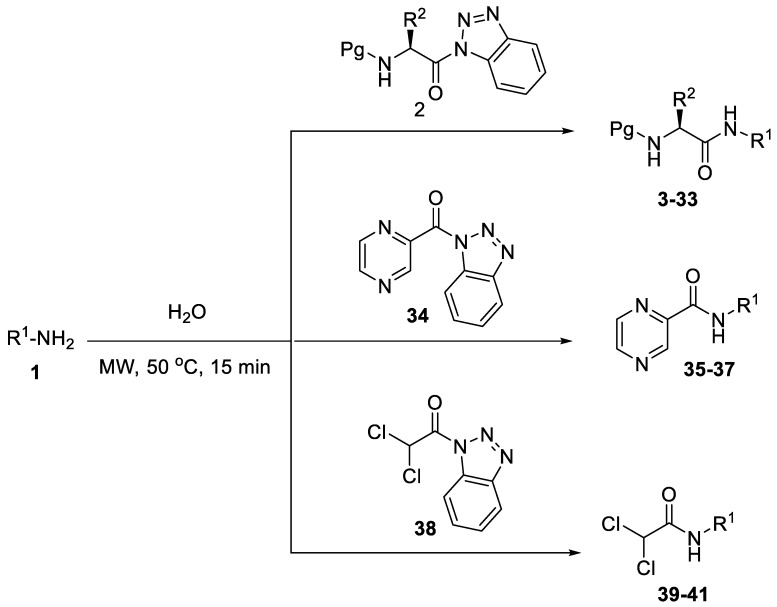
Synthesis of *N*-acylated amines.

We therefore ran the reactions of aromatic amines with benzotriazolides of protected amino acids in water, under microwave irradiation for 15 to 20 min (Scheme 1). Our reaction condition yields pure *N*-acylated products for all three types of protected amino acids (Boc, Cbz and Fmoc) with various substituted anilines (Table 2). We believe the driving force of the reaction is controlled by diffusion, since both of the reactants are water insoluble and form a heterogeneous reaction mixture. To justify our hypothesis, we used hexanes, a non-polar solvent, as a reaction medium in which both reactants are insoluble, and we found an equivalent outcome. We thus preferred nonflammable water over hexanes in our reactions. To explore the use of our reaction condition, we used different amines with various benzotriazolides. Our optimized reaction condition retains the chirality of the products, which was confirmed by performing reactions with both the DL and L forms of amino acids. High-performance liquid chromatography (HPLC) analysis of compound **9** (contains *L*-alanine) showed a single peak, with nearly the same retention time (16.290 min) as that of one of the two peaks (16.813 and 18.407 min) obtained from the mixture of the racemic compound **9+9′** (contains *DL*-alanine) with the enantiopure compound **9**. The increase in height of one peak supports the retention of chiral integrity in our reaction protocol (Supplementary Material).

**Scheme 2:**
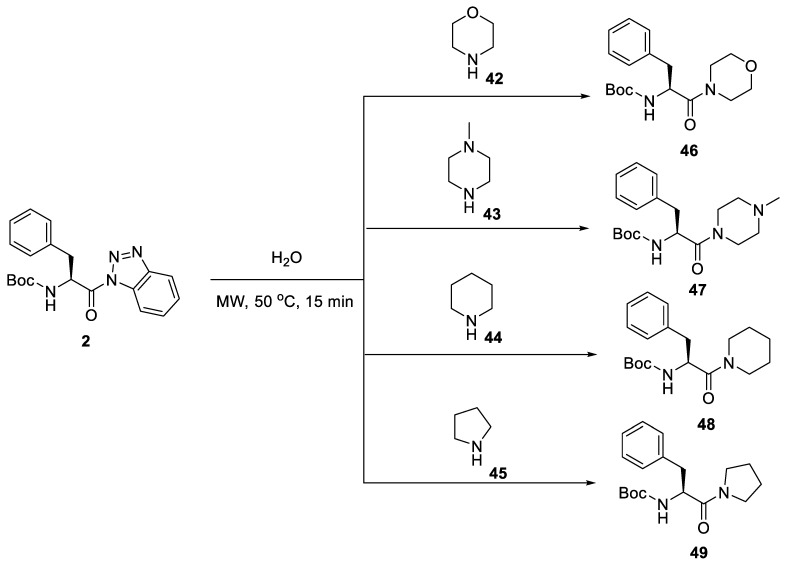
Synthesis of Boc-protected amino acid with secondary heterocyclic amine conjugates.

In addition to the primary amines, we also tried our optimized reaction condition with secondary amines. We were able to get the *N*-acylated secondary amines in good yields with high purity (Scheme 2). Earlier, we reported these conjugates, which were synthesized by treating benzotriazolide of boc-protected amino acids with secondary amines in the presence of triethylamine in tetrahydrofuran (THF) [35].

**Scheme 3:**
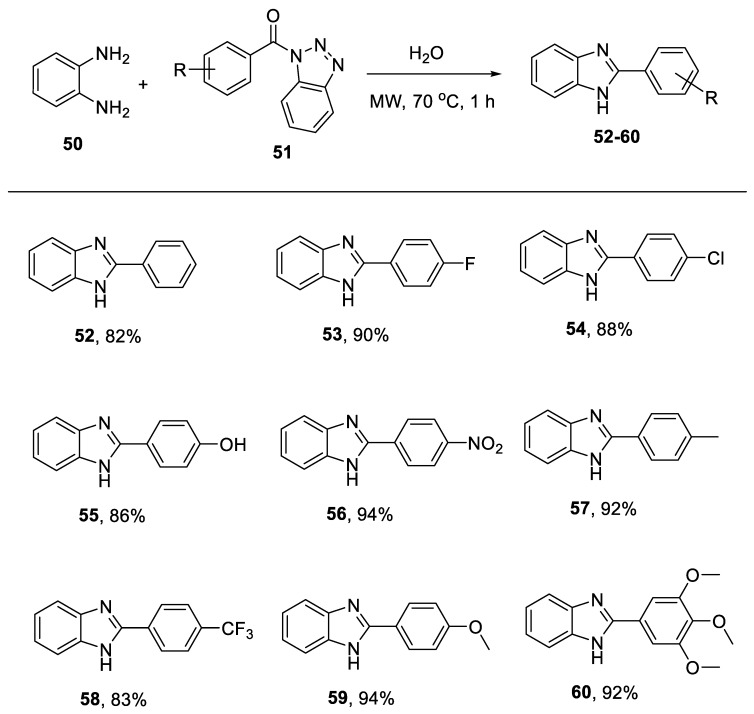
Synthesis of 2-substituted benzimidazoles.

Despite tremendous success in the synthesis of 2-substituted benzimidazoles, many of the methodologies suffer from one or more limitations, such as long reaction times, the formation of several side products, harsh reaction conditions, low yields, complicated work-up procedures and the generation of acidic and metallic wastes. As a consequence, the development of a new method, or technical improvement of the existing methods, is still an important experimental challenge. To expand the range of applicability of our optimized greener protocol, we treated benzotriazole-activated substituted benzoic acids with o-phenylenediamine in water, under microwave conditions. We obtained our desired product in 1 h (Scheme 3).

To elucidate the use of water in our reaction protocol, we added 5 mol% of a phase-transfer catalyst (Aliquat 336), which lowered the yields of the products, again supporting our proposed reaction mechanism of diffusion. The physical state of the reactants is also important: the use of microwave irradiation over conventional heating significantly improved the yields and purity, with retention of the chiral integrity. All the synthesized compounds were fully characterized by spectral studies (Supplementary Material).

## 3. Conclusions

In conclusion, we report mild, fast, efficient, facile and green conditions for the N-acylation of amines in water, without the use of catalyst or reagent. The heterogeneous reaction runs in water and forms the N-acylated products without loss of chirality and with high yields. The optimized reaction conditions work well at room temperature as well as under microwave irradiation for small-scale reactions, but for large-scale reactions microwave conditions are preferred. The application of microwaves and the concept of a heterogeneous reaction mixture expands the use of the reaction condition for the synthesis of 2-substituted benzimidazoles. Given its qualities of being racemization-free, high yield, catalyst- and solvent-free and ecofriendly, as well as the possibility of it scaling-up, the reaction has substantial potential for implementation by the pharmaceutical and agriculture industries.

## 4. Experimental Section

Melting points were determined on a capillary point apparatus equipped with a digital thermometer. NMR spectra were recorded in (DMSO-d_6_) on Bruker NMR spectrometers operating at 500 MHz for 1H [with tetramethysilane (TMS) as an internal standard] and 125 MHz for 13C. All microwave-assisted reactions were carried out with a single mode cavity Discover Microwave Synthesizer (CEM Corporation, Charlotte, NC, USA). The reaction mixtures were transferred into a 10 mL glass pressure microwave tube equipped with a magnetic stirrer bar. The tube was closed with a silicon septum and the reaction mixture was subjected to microwave irradiation (Discover mode; run time: 60 s; Power Max-cooling mode). HPLC analysis was carried out on Agilent 6120 LCMS instrument with Chirobiotic T column.

### 4.1. General Methods for N-acylation

In a typical procedure, a mixture of amine (1 equiv.) and N-protected aminoacylbenzotriazole or arylylbenzotriazole (1 equiv.) was subjected to microwave irradiation (20 W, 50 °C) in water (3 mL) for 15–20 min. After completion of the reaction, aqueous Na_2_CO_3_ or 4N HCl was added and the mixture was extracted with ethyl acetate or filter the precipitates, followed by washing with water. In most of the cases the isolated products were in pure form, and some were recrystallized in ethanol. Benzotriazoles could be recovered from the aqueous layer by pH-controlled acidification.

*tert-Butyl (S)-(1-oxo-3-phenyl-1-(phenylamino)propan-2-yl)carbamate***(3)**. White microcrystals (94%); m.p. 137–139 °C (Lit. m.p. 138–139 °C [57]). ^1^H NMR (500 MHz, DMSO-*d**_6_***) δ: 10.01 (s, 1H), 7.58 (d, *J* = 7.8 Hz, 1H), 7.44–6.90 (m, 10H), 4.32 (s, 1H), 3.49–1.79 (m, 2H), 2.01–0.59 (m, 9H). ^13^C NMR (125 MHz, DMSO-*d**_6_***) δ: 171.2, 155.9, 139.4, 138.4, 129.9, 129.7, 129.6, 129.3, 129.2, 128.6, 128.5, 126.7, 126.7, 123.8, 119.9, 119.8, 78.6, 57.0, 37.9, 28.6. HRMS *m*/*z* calcd for C_20_H_24_N_2_O_3_ [M + H]^+^ 341.1787, found 341.1789.

*tert-Butyl (S)-(3-methyl-1-oxo-1-(phenylamino)butan-2-yl)carbamate***(4)**. White microcrystals (93%); m.p. 123–125 °C (Lit. m.p. 120–121 °C [58]). ^1^H NMR (500 MHz, DMSO-*d*_6_) δ 9.95 (s, 1H), 7.60 (d, *J* = 7.4 Hz, 2H), 7.30 (t, *J* = 7.2 Hz, 2H), 7.05 (t, *J* = 7.5 Hz, 1H), 6.82 (d, *J* = 6.7 Hz, 1H), 3.94 (t, *J* = 8.6 Hz, 1H), 2.06–1.94 (m, 1H), 1.39 (s, 9H), 0.90 (d, *J* = 5.1 Hz, 6H); ^13^C NMR (125 MHz, DMSO-*d*_6_) *δ* 171.2, 156.1, 139.3, 129.2, 123.8, 119.7, 78.5, 61.0, 30.9, 28.7, 19.7. HRMS *m*/*z* calcd for C_16_H_24_N_2_O_3_ [M + H]^+^ 293.1787, found 293.1786. 

*Benzyl (2-oxo-2-(phenylamino)ethyl)carbamate***(5)**. White microcrystals (95%); m.p. 145–146 °C (Lit. m.p. 148–149 °C [59]). ^1^H NMR (500 MHz, DMSO-*d*_6_) δ 9.99 (s, 1H), 7.61 (d, *J* = 7.6 Hz, 2H), 7.55 (t, *J* = 6.8 Hz, 1H), 7.43–7.23 (m, 7H), 7.05 (t, *J* = 6.8 Hz, 1H), 5.07 (s, 2H), 3.84 (d, *J* = 5.7 Hz, 2H); ^13^C NMR (125 MHz, DMSO-*d*_6_) *δ* 168.4, 157.1, 139.4, 137.5, 129.2, 128.8, 128.3, 128.2, 123.7, 119.6, 66.0, 44.6. HRMS *m*/*z* calcd for C_16_H_16_N_2_O_3_ [M + H]^+^ 285.1161, found 285.1169.

*(9H-Fluoren-9-yl)methyl (2-oxo-2-(phenylamino)ethyl)carbamate***(6)**. White microcrystals (91%); m.p. 165–167 °C [60]. ^1^H NMR (500 MHz, DMSO-*d*_6_) δ 10.02 (s, 1H), 7.89 (d, *J* = 7.4 Hz, 2H), 7.74 (d, *J* = 7.3 Hz, 2H), 7.66–7.58 (m, 3H), 7.42 (t, *J* = 7.2 Hz, 2H), 7.36–7.29 (m, 4H), 7.05 (t, *J* = 6.4 Hz, 1H), 4.33 (d, *J* = 6.7 Hz, 2H), 4.25 (t, *J* = 6.5 Hz, 1H), 3.84 (d, *J* = 5.7 Hz, 2H).; ^13^C NMR (125 MHz, DMSO-*d*_6_) *δ* 168.4, 157.1, 144.3, 141.2, 139.4, 129.9, 129.2, 128.1, 127.6, 125.7, 123.7, 120.6, 119.6, 66.2, 47.1, 44.5. HRMS *m*/*z* calcd for C_23_H_20_N_2_O_3_ [M + H]^+^ 373.1474, found 373.1477. 

*tert-Butyl (R)-(3-methyl-1-oxo-1-(p-tolylamino)butan-2-yl)carbamate***(7)**. Yellow microcrystals (95%); m.p. 118–120 °C (Lit. m.p. 115–117 °C [58]). ^1^H NMR (500 MHz, DMSO-*d*_6_) δ 9.05 (s, 1H), 7.39 (d, *J* = 7.6 Hz, 2H), 6.99 (d, *J* = 5.0 Hz, 2H), 5.79 (d, *J* = 9.2 Hz, 1H), 4.25 (t, *J* = 7.9 Hz, 1H), 2.28 (s, 3H), 2.21–2.13 (m, 1H), 1.43 (s, 9H), 1.05 (d, *J* = 7.1 Hz, 6H); ^13^C NMR (125 MHz, DMSO-*d*_6_) *δ* 170.8, 156.6, 135.4, 133.5, 129.2, 120.2, 80.0, 60.8, 31.3, 28.4, 20.8, 19.3. HRMS *m*/*z* calcd for C_17_H_26_N_2_O_3_ [M + H]^+^ 307.1943, found 307.1944.

*Benzyl (2-oxo-2-(p-tolylamino)ethyl)carbamate***(8)**. White microcrystals (88%); m.p. 152–154 °C (Lit. m.p. 153–154 °C [61]). ^1^H NMR (500 MHz, DMSO-*d**_6_***) δ: 9.86 (s, 1H), 7.52 (t, *J* = 5.8 Hz, 1H), 7.46 (d, *J* = 8.2 Hz, 1H), 7.40–7.29 (m, 6H), 7.10 (d, *J* = 8.2 Hz, 2H), 5.05 (s, 2H), 3.79 (d, *J* = 6.1 Hz, 2H), 2.24 (s, 3H). ^13^C NMR (125 MHz, DMSO-*d**_6_***) δ: 167.6, 156.6, 137.0, 136.4, 132.1, 129.1, 128.4, 128.3, 128.2, 127.9, 127.8, 127.7, 126.4, 119.1, 65.5, 43.9, 20.4. HRMS *m*/*z* calcd for C_17_H_18_N_2_O_3_ [M + H]^+^ 299.1317, found 299.1319.

*Benzyl (S)-(1-oxo-1-(p-tolylamino) propan-2-yl) carbamate***(9)**. White microcrystals (96%); m.p. 158–160 °C (Lit. m.p. 160–162 °C [59]). ^1^H NMR (500 MHz, DMSO-*d**_6_***) δ: 9.86 (s, 1H), 7.56 (d, *J* = 7.2 Hz, 1H), 7.47 (d, *J* = 8.0 Hz, 1H), 7.41–7.24 (m, 6H), 7.10 (d, *J* = 8.1 Hz, 2H), 5.02 (q, *J* = 12.6 Hz, 2H), 4.37–3.96 (m, 1H), 2.25 (s, 3H), 1.28 (d, *J* = 7.1 Hz, 3H). ^13^C NMR (125 MHz, DMSO-*d**_6_***) δ: 171.2, 155.7, 136.9, 136.5, 132.1, 129.0, 129.0, 128.4, 128.3, 127.8, 127.7, 127.5, 119.2, 118.4, 65.4, 50.7, 20.4, 18.1. HRMS *m*/*z* calcd for C_18_H_20_N_2_O_3_ [M + H]^+^ 313.1474, found 313.1481.

*Benzyl (RS)-(1-oxo-1-(p-tolylamino) propan-2-yl) carbamate***(9+9′)**. White solid (92%), m.p. 152–154 °C. 1H NMR (500 MHz, DMSO-d6) δ: 9.86 (s, 1H), 7.56 (s, 1H), 7.47 (d, J = 8.0 Hz, 2H), 7.39–7.26 (m, 5H), 7.10 (d, J = 8.2 Hz, 2H), 5.02 (q, J = 12.6 Hz, 2H), 4.34–4.01 (m, 1H), 2.25 (s, 3H), 1.28 (d, J = 7.1 Hz, 3H). 13C NMR (125 MHz, DMSO-d6) δ: 171.2, 155.7, 136.9, 136.5, 132.1, 129.1, 129.0, 128.4, 128.3, 127.8, 127.8, 127.7, 119.2, 119.2, 65.3, 50.7, 20.4, 18.1. HRMS m/z calcd for C18H20N2O3 [M + H]+ 313.1474, found 313.1488. 

*Benzyl (S)-(3-methyl-1-oxo-1-(p-tolylamino)butan-2-yl)carbamate***(10)**. White microcrystals (97%); m.p. 183–185 °C [62]. ^1^H NMR (500 MHz, DMSO-*d*_6_) δ 9.98 (s, 1H), 7.60–7.25 (m, 8H), 7.15 (d, *J* = 6.4 Hz, 2H), 5.09 (s, 2H), 4.03 (t, *J* = 6.4 Hz, 1H), 2.29 (s, 3H), 2.15–1.94 (m, 1H), 0.95 (d, *J* = 3.9 Hz, 6H); ^13^C NMR (125 MHz, DMSO-*d*_6_) *δ* 170.7, 156.8, 137.5, 136.8, 132.8, 129.6, 128.9, 128.3, 128.2, 119.8, 66.0, 61.5, 30.9, 20.9, 19.7. HRMS *m*/*z* calcd for C_20_H_24_N_2_O_3_ [M + H]^+^ 341.1787, found 341.1788.

*Benzyl (S)-(4-(methylthio)-1-oxo-1-(p-tolylamino)butan-2-yl)carbamate***(11)**. White microcrystals (79%); m.p. 185–187 °C. ^1^H NMR (500 MHz, DMSO-*d**_6_***) δ: 9.94 (s, 1H), 7.63 (d, *J* = 7.8 Hz, 1H), 7.48 (d, *J* = 8.2 Hz, 2H), 7.42–7.25 (m, 5H), 7.11 (d, *J* = 8.2 Hz, 2H), 5.07–4.99 (m, 2H), 4.22 (dd, *J* = 13.2, 8.5 Hz, 1H), 2.66–2.32 (m, 2H), 2.25 (s, 3H), 2.04 (s, 3H), 2.05–1.69 (m, 2H). ^13^C NMR (125 MHz, DMSO-*d**_6_***) δ: 170.2, 156.1, 136.9, 136.3, 132.2, 129.1, 129.0, 128.3, 128.3, 127.8, 127.8, 127.7, 119.3, 119.3, 65.4, 54.6, 31.6, 29.7, 20.4, 14.6. HRMS *m*/*z* calcd for C_20_H_24_N_2_O_3_S [M + H]^+^ 372.1508, found 372.1512.

*(9H-Fluoren-9-yl)methyl (S)-(1-oxo-3-phenyl-1-(p-tolylamino)propan-2-yl)carbamate***(12)**. White microcrystals (94%); m.p. 193–195 °C. ^1^H NMR (500 MHz, DMSO-*d*_6_) δ 10.03 (s, 1H), 7.86 (d, *J* = 7.3 Hz, 2H), 7.73 (d, *J* = 8.3 Hz, 1H), 7.64 (t, *J* = 7.5 Hz, 2H), 7.47 (d, *J* = 7.8 Hz, 2H), 7.42–7.24 (m, 9H), 7.11 (d, *J* = 7.7 Hz, 2H), 4.48–4.35 (m, 1H), 4.23–4.14 (m, 3H), 3.08–3.00 (m, 1H), 2.96–2.84 (m, 1H), 2.24 (s, 3H); ^13^C NMR (125 MHz, DMSO-*d*_6_) *δ* 170.7, 156.4, 144.1, 141.1, 138.3, 136.7, 133.0, 129.7, 129.6, 128.6, 128.1, 127.5, 126.9, 125.7, 120.5, 119.9, 66.2, 57.3, 47.0, 38.0, 20.9. HRMS *m*/*z* calcd for C_31_H_28_N_2_O_3_ [M + H]^+^ 477.2100, found 477.2109.

*(9H-Fluoren-9-yl)methyl (1-oxo-1-(p-tolylamino)propan-2-yl)carbamate***(13)**. White microcrystals (75%); m.p. 172–174 °C. ^1^H NMR (500 MHz, DMSO-*d**_6_***) δ: 9.87 (s, 1H), 7.89 (d, *J* = 7.5 Hz, 2H), 7.76–7.54 (m, 3H), 7.51–7.43 (m, 2H), 7.44–7.29 (m, 4H), 7.10 (d, *J* = 8.2 Hz, 2H), 4.39–4.09 (m, 4H), 2.25 (s, 3H), 1.43–1.21 (m, 3H). ^13^C NMR (125 MHz, DMSO-*d**_6_***) δ: 171.3, 155.8, 143.9, 140.7, 136.5, 132.1, 129.0, 127.6, 127.0, 125.3, 120.1, 119.1, 65.6, 50.7, 46.6, 20.4, 18.1. HRMS *m*/*z* calcd for C_25_H_24_N_2_O_3_ [M + H]^+^ 401.1787, found 401.1785.

*Benzyl (S)-(1-((4-methoxyphenyl)amino)-1-oxo-3-phenylpropan-2-yl)carbamate***(14)**. White microcrystals (80%); m.p. 164–166 °C [Lit. m.p. 167 °C [63]]. ^1^H NMR (500 MHz, DMSO-*d**_6_***) δ: 9.94 (s, 1H), 7.64 (d, *J* = 8.4 Hz, 1H), 7.48 (d, *J* = 8.9 Hz, 2H), 7.36–7.20 (m, 10H), 6.88 (d, *J* = 9.0 Hz, 2H), 4.96 (s, 2H), 4.38 (td, *J* = 9.7, 4.8 Hz, 1H), 3.72 (s, 3H), 3.02 (dd, *J* = 13.6, 4.6 Hz, 1H), 2.85 (dd, *J* = 15.5, 8.2 Hz, 1H). ^13^C NMR (125 MHz, DMSO-*d**_6_***) δ: 169.9, 155.9, 155.3, 137.9, 136.9, 131.9, 129.8, 129.21, 129.1, 128.3, 128.2, 128.0, 127.7, 127.5, 127.4, 126.5, 126.3, 124.9, 120.9, 113.8, 65.3, 56.8, 55.1, 37.6. HRMS *m*/*z* calcd for C_24_H_24_N_2_O_4_ [M + H]^+^ 405.1736, found 405.1737.

*Benzyl (S)-(1-((4-methoxyphenyl)amino)-1-oxopropan-2-yl)carbamate***(15)**. White microcrystals (85%); m.p. 160–162 °C (Lit. m.p. 161.5–162.5 °C [64]). ^1^H NMR (500 MHz, DMSO-*d**_6_***) δ: 9.88 (s, 1H), 7.63–7.45 (m, 3H), 7.42–7.27 (m, 5H), 6.88 (d, *J* = 9.0 Hz, 2H), 5.03 (q, *J* = 12.6 Hz, 2H), 4.35–4.04 (m, 1H), 3.72 (s, 3H), 1.29 (d, *J* = 7.1 Hz, 3H). ^13^C NMR (125 MHz, DMSO-*d**_6_***) δ: 171.0, 155.7, 154.8, 137.0, 132.2, 132.1, 128.4, 128.3, 127.8, 127.7, 125.4, 125.3, 120.7, 113.8, 65.4, 55.2, 50.7, 18.2. HRMS *m*/*z* calcd for C_18_H_20_N_2_O_4_ [M + H]^+^ 329.1423, found 329.1431.

*Benzyl (S)-(1-((2-methoxyphenyl)amino)-1-oxopropan-2-yl)carbamate***(16)**. White microcrystals (98%); m.p. 185–187 °C [65]. ^1^H NMR (500 MHz, DMSO-*d**_6_***) δ: 9.04 (s, 1H), 8.03 (d, *J* = 7.4 Hz, 1H), 7.75 (d, *J* = 6.7 Hz, 1H), 7.47–7.21 (m, 5H), 7.16–6.97 (m, 2H), 6.91 (t, *J* = 8.2 Hz, 1H), 5.06 (q, *J* = 12.4 Hz, 2H), 4.45–4.18 (m, 1H), 3.81 (s, 3H), 1.29 (d, *J* = 7.1 Hz, 3H). ^13^C NMR (125 MHz, DMSO-*d**_6_***) δ: 171.3, 155.9, 148.9, 136.9, 128.4, 128.4, 127.8, 127.7, 127.1, 124.2, 124.2, 120.6, 120.3, 111.1, 65.5, 55.8, 50.9, 17.8. HRMS *m*/*z* calcd for C_18_H_20_N_2_O_4_ [M + H]^+^ 329.1423, found 329.1422.

*tert-Butyl (S)-(1-((4-fluorophenyl)amino)-3-methyl-1-oxobutan-2-yl)carbamate***(17)**. Yellow microcrystals (94%); m.p. 138–140 °C (Lit. m.p. 141–142 °C [58]). ^1^H NMR (500 MHz, DMSO-*d*_6_) δ 10.02 (s, 1H), 7.62 (dd, *J* = 7.9, 5.1 Hz, 2H), 7.13 (t, *J* = 8.7 Hz, 2H), 6.83 (d, *J* = 8.2 Hz, 1H), 3.91 (t, *J* = 7.6 Hz, 1H), 2.04–1.94 (m, 1H), 1.39 (s, 9H), 0.90 (d, *J* = 6.3 Hz, 6H); ^13^C NMR (125 MHz, DMSO-*d*_6_) *δ* 171.1, 159.4, 157.5, 156.1, 135.7, 121.5, 121.4, 115.8, 115.6, 78.5, 61.0, 30.8, 28.6, 19.6. HRMS *m*/*z* calcd for C_16_H_23_FN_2_O_3_ [M + H]^+^ 311.1693, found 311.1699.

*Benzyl (S)-(1-((4-fluorophenyl) amino)-1-oxo-3-phenylpropan-2-yl) carbamate***(18)**. White microcrystals (77%); m.p. 191–193 °C (Lit. m.p. 195 °C [63]). ^1^H NMR (500 MHz, DMSO-*d**_6_***) δ: 9.94 (s, 1H), 7.64 (d, *J* = 8.4 Hz, 1H), 7.48 (d, *J* = 8.9 Hz, 2H), 7.36–7.20 (m, 10H), 6.88 (d, *J* = 9.0 Hz, 2H), 4.96 (s, 2H), 4.38 (td, *J* = 9.7, 4.8 Hz, 1H), 3.72 (s, 3H), 3.02 (dd, *J* = 13.6, 4.6 Hz, 1H), 2.85 (dd, *J* = 15.5, 8.2 Hz, 1H). ^13^C NMR (125 MHz, DMSO-*d**_6_***) δ: 169.9, 155.9, 155.3, 137.9, 136.9, 131.9, 129.8, 129.21, 129.1, 128.3, 128.2, 128.0, 127.7, 127.5, 127.4, 126.5, 126.3, 124.9, 120.9, 113.8, 65.3, 56.8, 55.1, 37.6. HRMS *m*/*z* calcd for C_23_H_21_FN_2_O_3_ [M + H]^+^ 393.1536, found 393.1533.

*Benzyl (2-((4-fluorophenyl)amino)-2-oxoethyl)carbamate***(19)**. White microcrystals (84%); m.p. 174–176 °C. ^1^H NMR (500 MHz, DMSO-*d**_6_***) δ: 10.02 (s, 1H), 7.61 (dd, *J* = 8.8, 5.0 Hz, 2H), 7.56 (t, *J* = 6.0 Hz, 1H), 7.41–7.32 (m, 6H), 7.15 (t, *J* = 8.9 Hz, 1H), 5.06 (s, 2H), 3.80 (d, *J* = 6.1 Hz, 2H). ^13^C NMR (125 MHz, DMSO-*d**_6_***) δ: 167.8, 158.9, 156.6, 137.0, 135.3, 128.4, 128.3, 128.2, 127.8, 127.7, 120.8, 120.8, 115.4, 115.2, 65.5, 43.9. HRMS *m*/*z* calcd for C_16_H_15_FN_2_O_3_ [M + H]^+^ 303.1067, found 303.1066.

*Benzyl (S)-(1-((4-fluorophenyl)amino)-1-oxopropan-2-yl)carbamate***(20)**. White microcrystals (90%); m.p. 192–194 °C [65]. ^1^H NMR (500 MHz, DMSO-*d**_6_***) δ: 10.03 (s, 1H), 7.69–7.51 (m, 3H), 7.44–7.25 (m, 5H), 7.23–7.04 (m, 2H), 5.03 (q, *J* = 12.7 Hz, 2H), 4.32–3.96 (m, 1H), 1.29 (d, *J* = 7.1 Hz, 3H). ^13^C NMR (125 MHz, DMSO-*d**_6_***) δ: 171.4, 158.9, 155.8, 136.9, 135.4, 128.3, 128.30, 127.8, 127.71, 127.7, 120.9, 120.9, 115.3, 115.1, 65.4, 50.7, 17.9. HRMS *m*/*z* calcd for C_17_H_17_FN_2_O_3_ [M + H]^+^ 317.1223, found 317.1228.

*(9H-Fluoren-9-yl)methyl (2-((4-fluorophenyl)amino)-2-oxoethyl)carbamate***(21)**. Yellow microcrystals (90%); m.p. 142–143 °C. ^1^H NMR (500 MHz, DMSO-*d*_6_) δ 10.20 (s, 1H), 7.66 (d, *J* = 8.0 Hz, 1H), 7.64–7.54 (m, 2H), 7.36–7.19 (m, 10H), 7.15 (t, *J* = 8.4 Hz, 2H), 4.97 (s, 2H), 4.45–4.36 (m, 1H), 3.07–3.00 (m, 1H), 2.90–2.82 (m, 1H); ^13^C NMR (125 MHz, DMSO-*d*_6_) *δ* 170.9, 159.6, 157.6, 156.5, 138.1, 137.4, 135.6, 129.7, 128.8, 128.6, 128.2, 128.0, 126.9, 121.7, 121.7, 115.8, 115.7, 65.8, 57.4, 37.9. HRMS *m*/*z* calcd for C_23_H_19_FN_2_O_3_ [M + H]^+^ 391.1380, found 391.1393.

*tert-Butyl (S)-(1-((4-chlorophenyl)amino)-1-oxo-3-phenylpropan-2-yl)carbamate***(22)**. White microcrystals (98%); m.p. 152–154 °C [66]. ^1^H NMR (500 MHz, DMSO-*d**_6_***) δ: 10.17 (s, 1H), 7.61 (d, *J* = 8.8 Hz, 2H), 7.50–7.00 (m, 8H), 4.31 (dd, *J* = 12.8, 9.2 Hz, 1H), 3.26–2.65 (m, 2H), 1.59–1.11 (m, 9H). ^13^C NMR (125 MHz, DMSO-*d**_6_***) δ: 170.9, 155.4, 138.0, 137.8, 129.3, 129.2, 129.0, 128.8, 128.6, 128.1, 128.0, 126.8, 126.3, 126.3, 120.8, 78.1, 56.6, 55.1, 37.3, 28.1. HRMS *m*/*z* calcd for C_20_H_23_ClN_2_O_3_ [M + H]^+^ 375.1397, found 375.1389.

*Benzyl (2-((4-chlorophenyl)amino)-2-oxoethyl)carbamate***(23)**. White microcrystals (95%); m.p. 160–162 °C. ^1^H NMR (500 MHz, DMSO-*d**_6_***) δ: 10.10 (s, 1H), 7.62 (d, *J* = 8.7 Hz, 2H), 7.56 (t, *J* = 5.9 Hz, 1H), 7.42–7.29 (m, 7H), 5.05 (s, 2H), 3.81 (d, *J* = 6.1 Hz, 2H). ^13^C NMR (125 MHz, DMSO-*d**_6_***) δ: 168.6, 157.1, 138.3, 137.5, 131.5, 129.1, 128.9, 128.8, 128.7, 128.3, 128.2, 127.2, 127.1, 121.1, 65.9, 44.6. HRMS *m*/*z* calcd for C_16_H_15_ClN_2_O_3_ [M + H]^+^ 319.0771, found 319.0777.

*Benzyl (S)-(1-((4-chlorophenyl)amino)-1-oxo-3-phenylpropan-2-yl)carbamate***(24)**. White microcrystal (90%); m.p. 182–184 °C. ^1^H NMR (500 MHz, DMSO-*d**_6_***) δ: 10.29 (s, 1H), 7.76 (d, *J* = 7.8 Hz, 1H), 7.63 (d, *J* = 8.8 Hz, 2H), 7.44–7.23 (m, 11H), 7.21 (d, *J* = 7.2 Hz, 1H), 4.96 (s, 2H), 4.80–3.81 (m, 1H), 3.02 (dd, *J* = 13.7, 4.6 Hz, 1H), 2.86 (dd, *J* = 13.6, 10.3 Hz, 1H). ^13^C NMR (125 MHz, DMSO-*d**_6_***) δ: 171.2, 156.4, 138.3, 138.2, 137.4, 130.0, 129.8, 129.7, 129.1, 128.8, 128.8, 128.7, 128.6, 128.2, 128.0, 127.8, 127.4, 127.3, 126.9, 121.3, 65.8, 57.5, 37.9. HRMS *m*/*z* calcd for C_23_H_21_ClN_2_O_3_ [M + H]^+^ 409.1241, found 409.1244.

*Benzyl (S)-(1-((4-chlorophenyl)amino)-1-oxopropan-2-yl)carbamate***(25)**. White microcrystal (73%); m.p. 163–165 °C (Lit. m.p. 165–166 °C [67]). ^1^H NMR (500 MHz, DMSO-*d_6_*) δ: 10.11 (s, 1H), 7.72–7.55 (m, 3H), 7.42–7.28 (m, 7H), 5.53–4.69 (m, 2H), 4.50–3.73 (m, 1H), 1.29 (d, *J* = 7.1 Hz, 3H). ^13^C NMR (125 MHz, DMSO-*d**_6_***) δ: 171.7, 155.78, 137.9, 136.9, 128.6, 128.6, 128.3, 128.3, 127.8, 127.7, 126.8, 126.7, 120.8, 120.7, 65.4, 50.8, 17.9. HRMS *m*/*z* calcd for C_17_H_17_ClN_2_O_3_ [M + H]^+^ 333.0928, found 333.0923. 

*Benzyl (S)-(1-((4-chlorophenyl)amino)-4-(methylthio)-1-oxobutan-2-yl)carbamate***(26)**. White microcrystal (82%); m.p. 150–152 °C [68]. ^1^H NMR (500 MHz, DMSO-*d**_6_***) δ: 10.20 (s, 1H), 8.28–7.57 (m, 3H), 7.47–7.27 (m, 7H), 5.11–4.97 (m, 2H), 4.24 (dd, *J* = 12.9, 8.5 Hz, 1H), 2.84–2.29 (m, 2H), 2.06 (s, 3H), 1.98–1.82 (m, 2H). ^13^C NMR (125 MHz, DMSO-*d**_6_***) δ: 170.7, 156.1, 137.8, 136.9, 131.7, 128.6, 128.3, 128.3, 127.8, 127.7, 127.7 126.9, 120.9, 119.7, 65.5, 54.7, 31.4, 29.7, 14.6. HRMS *m*/*z* calcd for C_19_H_21_ClN_2_O_3_S [M + H]^+^ 393.0961, found 393.0969.

*(9H-Fluoren-9-yl)methyl (1-((4-chlorophenyl)amino)-1-oxopropan-2-yl)carbamate***(27)**. White microcrystal (90%); m.p. 197–199 °C. ^1^H NMR (500 MHz, DMSO-*d**_6_***) δ: 10.13 (s, 1H), 7.89 (d, *J* = 7.4 Hz, 2H), 7.82–7.54 (m, 5H), 7.48–7.25 (m, 6H), 4.65–3.89 (m, 4H), 1.30 (d, *J* = 7.1 Hz, 3H). ^13^C NMR (125 MHz, DMSO-*d**_6_***) δ: 171.7, 155.8, 143.8, 143.8, 140.7, 137.9, 128.6, 127.6, 127.0, 126.7, 125.3, 125.3, 125.2, 120.7, 120.1, 65.6, 50.8, 46.6, 17.9. HRMS *m*/*z* calcd for C_24_H_21_ClN_2_O_3_ [M + H]^+^ 421.1241, found 421.1235.

*(9H-Fluoren-9-yl)methyl (S)-(1-((4-chlorophenyl)amino)-1-oxo-3-phenylpropan-2-yl)carbamate***(28)**. White microcrystal; (98%); m.p. 210–212 °C. ^1^H NMR (500 MHz, DMSO-*d**_6_***) δ: 7.92–7.80 (m, 4H), 7.75–7.59 (m, 2H), 7.41 (dd, *J* = 11.1, 4.6 Hz, 3H), 7.37–7.02 (m, 10H), 6.27 (s, 1H), 4.64–3.86 (m, 3H), 3.48–2.68 (m, 2H). ^13^C NMR (125 MHz, DMSO-*d**_6_***) δ: 172.2, 143.9, 142.6, 139.40, 137.4, 135.4, 133.2, 129.5, 129.4, 129.2, 128.9, 128.5, 128.0, 127.8, 127.3, 126.1, 125.7, 123.9, 122.3, 122.2, 121.4, 121.2, 120.7, 120.0, 115.3, 109.7, 79.2, 57.2, 53.8, 37.9. HRMS *m*/*z* calcd for C_30_H_25_ClN_2_O_3_ [M + H]^+^ 497.1554, found 497.1553.

*Benzyl (S)-(1-((2-chlorophenyl)amino)-1-oxopropan-2-yl)carbamate***(29)**. White microcrystal (70%); m.p. 162–164 °C. ^1^H NMR (500 MHz, DMSO-*d**_6_***) δ: 9.43 (s, 1H), 7.76 (d, *J* = 7.8 Hz, 1H), 7.70 (d, *J* = 6.8 Hz, 1H), 7.49 (d, *J* = 7.9 Hz, 1H), 7.42–7.28 (m, 6H), 7.19 (t, *J* = 7.5 Hz, 1H), 5.05 (s, 2H), 4.45–4.14 (m, 1H), 1.33 (d, *J* = 7.1 Hz, 3H). ^13^C NMR (125 MHz, DMSO-*d**_6_***) δ: 171.72, 155.9, 136.9, 134.56, 129.4, 129.4, 128.3, 128.3, 127.8, 127.7, 127.5, 126.2, 126.0, 125.3, 65.5, 50.6, 17.8. HRMS *m*/*z* calcd for C_17_H_17_ClN_2_O_3_ [M + H]^+^ 333.0928, found 333.0941.

*Benzyl (S)-(1-((4-nitrophenyl)amino)-1-oxopropan-2-yl)carbamate***(30)**. White microcrystal (70%); m.p. 95–97 °C (Lit. m.p. 99 °C [69]). ^1^H NMR (500 MHz, DMSO-*d**_6_***) δ: 8.93–8.01 (m, 3H), 7.82 (t, *J* = 7.6 Hz, 1H), 7.65 (t, *J* = 7.7 Hz, 1H), 7.54–7.20 (m, 4H), 7.16–6.81 (m, 1H), 5.59–5.38 (m, 1H), 5.12–4.95 (m, 2H), 1.54 (d, *J* = 7.2 Hz, 3H). ^13^C NMR (125 MHz, DMSO-*d**_6_***) δ: 172.4, 156.0, 145.3, 136.7, 131.2, 130.7, 128.4, 128.3, 127.9, 127.8, 127.7, 126.8, 120.2, 113.9, 65.8, 50.1, 16.8. HRMS *m*/*z* calcd for C_17_H_17_N_3_O_5_ [M + H]^+^ 344.1168, found 344.1160.

*Benzyl (2-((2-hydroxyphenyl)amino)-2-oxoethyl)carbamate***(31)**. White microcrystals (90%); m.p. 180–182 °C (Lit. m.p. 174–176 °C [65]). 1H NMR (500 MHz, DMSO-*d*_6_) δ 9.93 (s, 1H), 9.11 (s, 1H), 7.98–7.83 (m, 1H), 7.82–7.64 (m, 1H), 7.52–7.23 (m, 5H), 6.98–6.73 (m, 2H), 5.08 (s, 2H), 3.88 (d, *J* = 4.7 Hz, 2H); ^13^C NMR (125 MHz, DMSO-*d*_6_) *δ* 168.6, 157.2, 147.7, 137.4, 128.9, 128.3, 128.1, 126.5, 124.8, 121.6, 119.5, 115.7, 66.1, 44.9. HRMS *m*/*z* calcd for C_16_H_16_N_2_O_4_ [M + H]^+^ 301.1110, found 301.1117.

*Benzyl (S)-(1-oxo-1-(pyridin-4-ylamino)propan-2-yl)carbamate***(32)**. White microcrystals (89%); m.p. 152–154 °C [70]. ^1^H NMR (500 MHz, DMSO-*d**_6_***) δ: 8.43–8.14 (m, 4H), 7.82 (t, *J* = 7.7 Hz, 1H), 7.65 (t, *J* = 7.7 Hz, 1H), 7.47–7.22 (m, 5H), 5.51–5.49 (m, 1H), 5.07–5.02 (m, 2H), 1.54 (d, *J* = 7.2 Hz, 3H). ^13^C NMR (125 MHz, DMSO-*d**_6_***) δ: 172.4, 156.0, 145.3, 136.7, 131.1, 130.6, 128.4, 128.3, 127.9, 127.8, 126.7, 120.2, 113.9, 65.8, 50.1, 16.8. HRMS *m*/*z* calcd for C_16_H_17_N_3_O_3_ [M + H]^+^ 300.1270, found 300.1274.

*Benzyl (S)-(1-oxo-1-(pyridin-3-ylamino)propan-2-yl)carbamate***(33)**. White microcrystals (97%); m.p.155–157 °C. ^1^H NMR (500 MHz, DMSO-*d**_6_***) δ: 8.39–8.13 (m, 4H), 7.82 (t, *J* = 7.7 Hz, 1H), 7.65 (t, *J* = 7.7 Hz, 1H), 7.46–7.24 (m, 5H), 5.53–5.47 (m, 1H), 5.07–5.02 (m, 2H), 1.54 (d, *J* = 7.3 Hz, 3H). ^13^C NMR (125 MHz, DMSO-*d**_6_***) δ: 172.4, 156.0, 145.3, 137.0, 136.7, 131.1, 130.6, 128.4, 127.9, 127.8, 126.8, 120.2, 113.9, 65.8, 50.1, 16.8. HRMS *m*/*z* calcd for C_16_H_17_N_3_O_3_ [M + H]^+^ 300.1270, found 300.1265.

*N-(p-Tolyl)pyrazine-2-carboxamide***(35)**. White microcrystals (71%); m.p. 138–140 °C (Lit. m.p. 148 °C [72]). ^1^H NMR (500 MHz, DMSO-*d**_6_***) δ: 10.62 (s, 1H), 9.28 (d, *J* = 1.4 Hz, 1H), 8.92 (d, *J* = 2.5 Hz, 1H), 8.80 (dd, *J* = 2.5, 1.4 Hz, 1H), 7.77 (d, *J* = 8.4 Hz, 2H), 7.17 (d, *J* = 8.3 Hz, 2H), 2.29 (s, 3H). ^13^C NMR (125 MHz, DMSO-*d**_6_***) δ: 161.4, 147.6, 145.1, 143.9, 143.2, 135.6, 133.2, 129.04, 120.5, 20.5. HRMS *m*/*z* calcd for C_12_H_11_N_3_O [M + H]^+^ 214.0902, found 214.0913.

*N-(4-Methoxyphenyl)pyrazine-2-carboxamide***(36)**. White microcrystals (81%); m.p. 147–149 °C (Lit. m.p. 149–150 °C [73]). ^1^H NMR (500 MHz, DMSO-*d**_6_***) δ: 10.62 (s, 1H), 9.28 (d, *J* = 1.4 Hz, 1H), 8.92 (d, *J* = 2.5 Hz, 1H), 8.80 (dd, *J* = 2.5, 1.4 Hz, 1H), 7.80 (d, *J* = 9.0 Hz, 2H), 6.95 (d, *J* = 9.0 Hz, 2H), 3.75 (s, 3H). ^13^C NMR (125 MHz, DMSO-*d**_6_***) δ: 161.2, 155.9, 147.5, 145.2, 143.9, 143.2, 131.2, 122.0, 113.8, 55.2. HRMS *m*/*z* calcd for C_12_H_11_N_3_O_2_ [M + H]^+^ 230.0851, found 230.0843.

*N-(4-Fluorophenyl)pyrazine-2-carboxamide***(37)**. White microcrystals (98%); m.p. 155–157 °C (Lit. m.p. 154–155 °C [72]). ^1^H NMR (500 MHz, DMSO-*d**_6_***) δ: 10.82 (s, 1H), 9.30 (d, *J* = 1.4 Hz, 1H), 8.97 (d, *J* = 2.5 Hz, 1H), 8.81 (dd, *J* = 2.5, 1.4 Hz, 1H), 8.14–7.69 (m, 2H), 7.24–7.19 (m, 2H). ^13^C NMR (125 MHz, DMSO-*d**_6_***) δ: 161.6, 159.5, 157.6, 147.7, 144.9, 144.0, 143.2, 134.6, 122.5, 115.3, 115.2. HRMS *m*/*z* calcd for C_11_H_8_FN_3_O [M + H]^+^ 218.0651, found 218.0664.

*2,2-Dichloro-N-(p-tolyl) acetamide***(39)**. White microcrystals (85%); m.p. 157–159 °C (Lit. m.p. 159–160 °C [74]). ^1^H NMR (500 MHz, DMSO-*d**_6_***) δ: 10.56 (s, 1H), 7.48 (d, *J* = 8.4 Hz, 2H), 7.17 (d, *J* = 8.4 Hz, 2H), 6.58 (s, 1H), 2.27 (s, 3H). ^13^C NMR (125 MHz, DMSO-*d**_6_***) δ: 161.5, 135.0, 133.8, 129.4, 129.4, 119.8, 119.8, 67.3, 20.5. HRMS *m*/*z* calcd for C_9_H_9_Cl_2_NO [M + H]^+^ 218.0061, found 218.0057.

*2,2-Dichloro-N-(4-fluorophenyl) acetamide***(40)**. White microcrystals (80%); m.p. 130–132 °C (Lit. m.p. 134–135 °C [74]). ^1^H NMR (500 MHz, DMSO-*d**_6_***) δ: 10.71 (s, 1H), 7.71–7.55 (m, 2H), 7.24–7.19 (m, 2H), 6.59 (s, 1H). ^13^C NMR (126 MHz, DMSO-*d**_6_***) δ: 161.7, 159.7, 133.9, 121.8, 121.7, 115.8, 115.6, 67.3. HRMS *m*/*z* calcd for C_8_H_6_Cl_2_FNO [M + H]^+^ 221.9810, found 221.9813.

*2,2-Dichloro-N-(4-chlorophenyl) acetamide***(41)**. White microcrystals (80%); m.p. 143–145 °C (Lit. m.p. 141–142 °C [74]). ^1^H NMR (500 MHz, DMSO-*d**_6_***) δ: 10.79 (s, 1H), 7.63 (d, *J* = 8.8 Hz, 2H), 7.43 (d, *J* = 8.8 Hz, 2H), 6.59 (s, 1H). ^13^C NMR (125 MHz, DMSO-*d**_6_***) δ: 161.8, 136.5, 128.9, 128.3, 128.3, 121.4, 121.4, 67.2. HRMS *m*/*z* calcd for C_8_H_6_Cl_3_NO [M + H]^+^ 237.9515, found 237.9550.

### 4.2. General Methods for Preparation of 2-Substituted Benzimidazoles

In a typical procedure, a mixture of *o*-phenylenediamine (1 equiv.) and acylbenzotriazole (1 equiv.) was subjected to microwave irradiation (20 W, 50 °C) in water (3 mL) for 1 h. After completion of the reaction, aqueous 4N HCl was added and the precipitates were filtered, followed by washing with water. The isolated products were recrystallized in ethanol to get the desired benzimidazoles in pure form. Benzotriazoles could be recovered from the aqueous layer by pH-controlled acidification.

*2-Phenyl-1H-benzo[d]imidazole***(52)**. White microcrystals (82%); m.p. 295–296 °C (lit. m.p. 295 °C [75]). ^1^H NMR (500 MHz, DMSO-*d*_6_) *δ* 8.32–8.30 (m, 2H), 7.85–7.82 (m, 2H), 7.75–7.70 (m, 3H), 7.54–7.52 (m, 2H); ^13^C NMR (125 MHz, DMSO-*d*_6_) *δ* 151.2, 143.8, 134.9, 130.2, 129.5, 128.9, 126.4, 122.5, 121.6, 118.9, 111.3. HRMS *m*/*z* calcd for C_13_H_10_N_2_ [M + H]^+^ 195.0844, found 195.0856.

*2-(4-Fluorophenyl)-1H-benzo[d]imidazole***(53)**. White microcrystals (90%); m.p. 249–251 °C (lit. m.p. 248 °C [75]). ^1^H NMR (500 MHz, DMSO-*d*_6_) *δ* 8.24–8.21 (m, 2H), 7.60–7.59 (m, 2H), 7.22–7.17 (m, 4H); ^13^C NMR (125 MHz, DMSO-*d*_6_) *δ* 150.2, 147.7, 135.1, 134.5, 129.1, 129.0, 128.2, 122.8, 121.9, 116.9, 111.4. HRMS *m*/*z* calcd for C_13_H_9_FN_2_ [M + H]^+^ 213.0750, found 312.0746.

*2-(4-Chlorophenyl)-1H-benzo[d]imidazole***(54)**. White crystalline solid (88%); m.p. 299–301 °C (lit. m.p. 302 °C [75]). ^1^H NMR (500 MHz, DMSO-*d*_6_) *δ* 12.72 (s, 1H, D_2_O exchangable, > NH), 8.11–7.93 (m, 2H), 7.46–7.43 (m, 4H), 7.16–7.14 (m, 2H); ^13^C NMR (125 MHz, DMSO-*d*_6_) *δ* 150.1, 146.9, 135.0, 134.6, 129.1, 128.7, 128.6, 127.8, 121.8, 122.0, 117.2. HRMS *m*/*z* calcd for C_13_H_9_ClN_2_ [M + H]^+^ 229.0454, found 229.0458.

*4-(1H-Benzo[d]imidazol-2-yl)phenol***(55)**. Yellow microcrystals (86%); m.p. 280–282 °C (lit. m.p. 280 °C [75]). ^1^H NMR (500 MHz, DMSO-*d*_6_) *δ* 10.03 (s, 1H, D_2_O exchangable, > NH), 7.98 (d, *J* = 8.1 Hz, 2H), 7.55–7.48 (m, 2H), 7.20–7.14 (m, 2H), 6.91(d, *J* = 8.1 Hz, 2H); ^13^C NMR (125 MHz, DMSO-*d*_6_) *δ* 165.0, 157.1, 144.2, 133.3, 127.5, 125.9, 123.7, 121.4, 120.1, 115.5. HRMS *m*/*z* calcd for C_13_H_10_N_2_O [M + H]^+^ 211.0793, found 211.0792.

*2-(4-Nitrophenyl)-1H-benzo[d]imidazole***(56)**. Light yellow microcrystals (94%); m.p. 318–320 °C (lit. m.p. 317 °C [75]). ^1^H NMR (500 MHz, DMSO-*d*_6_) *δ* 13.05 (br s, 1H, D_2_O exchangable, > NH), 8.00 (dd, *J* = 15.4, 7.8 Hz, 2H), 7.87 (td, *J* = 7.8, 1.2 Hz, 1H), 7.76 (td *J* = 7.8, 1.4 Hz, 1H), 7.65–7.42 (m, 2H), 7.25 (d, *J* = 5.5 Hz, 2H); ^13^C NMR (125 MHz, DMSO-*d*_6_) *δ* 149.0, 147.6, 143.3, 134.6, 132.5, 130.7, 123.9, 123.1, 121.9, 119.2, 111.5. HRMS *m*/*z* calcd for C_13_H_9_N_3_O_2_ [M + H]^+^ 240.0695, found 240.0677.

*2-(4-Methylphenyl)-1H-benzo[d]imidazole***(57)**. White microcrystals (92%); m.p. 274–276 °C (lit. m.p. 275 °C [75]). ^1^H NMR (500 MHz, DMSO-*d*_6_) *δ* 12.59 (s, 1H, D_2_O exchangable, > NH), 8.08 (d, *J* = 8.1 Hz, 2H), 7.58–7.44 (m, 2H), 7.35 (d, *J* = 7.8 Hz, 2H), 7.12–7.11 (m, 2H), 2.35 (s, 3H); ^13^C NMR (125 MHz, DMSO-*d*_6_) *δ* 151.4, 139.4, 129.4, 127.4, 126.4, 121.9, 116.3, 21.0. HRMS *m*/*z* calcd for C_14_H_12_N_2_ [M + H]^+^ 209.1000, found 209.1018.

*2-(4-Trifluoromethylphenyl)-1H-benzo[d]imidazole***(58)**. White microcrystals (83%); m.p. 264–265 °C (lit. m.p. 263 °C [75]); ^1^H NMR (500 MHz, DMSO-*d*_6_) *δ* 12.79 (s, 1H, D_2_O exchangable, > NH), 8.34 (d, *J* = 8.8 Hz, 2H), 7.79–7.68 (m, 2H), 7.61 (d, *J* = 8.8 Hz, 2H), 7.04–6.96 (m, 2H); ^13^C NMR (125 MHz, DMSO-*d*_6_) *δ* 160.3, 151.3, 138.2, 127.7, 126.1, 124.5, 122.4, 121.4, 115.1. HRMS *m*/*z* calcd for C_14_H_9_F_3_N_2_ [M + H]^+^ 263.0718, found 263.0722.

*2-(4-Methoxyphenyl)-1H-benzo[d]imidazole***(59)**. White microcrystals (94%); m.p. 227–229 °C (lit. m.p. 228 °C [75]); ^1^H NMR (500 MHz, DMSO-*d*_6_) *δ* 12.74 (s, 1H, D_2_O exchangable, > NH), 8.17 (d, *J* = 8.8 Hz, 2H), 7.59–7.57 (m, 2H), 7.20–7.17 (m, 2H), 7.04 (d, *J* = 8.8 Hz, 2H), 3.86 (s, 3H); ^13^C NMR (125 MHz, DMSO-*d*_6_) *δ* 160.3, 151.3, 127.7, 130.1, 122.4, 121.4, 116.5, 114.3, 54.8. HRMS *m*/*z* calcd for C_14_H_12_N_2_O [M + H]^+^ 225.0950, found 225.0976.

*2-(3,4,5-Trimethoxyphenyl)-1H-benzo[d]imidazole***(60)**. Light yellow microcrystals (92%); m.p. 259–260 °C (lit. m.p. 259 °C [75]). ^1^H NMR (500 MHz, DMSO-*d*_6_) *δ* 7.78 (d, *J* = 7.8 Hz, 1H), 7.65 (d, *J* = 7.6 Hz, 1H), 7.45 (t, *J* = 7.2 Hz, 1H), 7.30 (t, *J* = 7.2 Hz, 1H), 7.01 (s, 2H), 4.04 (s, 6H), 3.88 (s, 3H); ^13^C NMR (125 MHz, DMSO-*d*_6_) *δ* 155.1, 153.1, 142.2, 138.4, 124.7, 123.2, 121.9, 108.8, 60.1, 57.4. HRMS *m*/*z* calcd for C_16_H_16_N_2_O_3_ [M + H]^+^ 285.1161, found 285.1168.

## Figures and Tables

**Table 1:** Optimization of reaction condition.

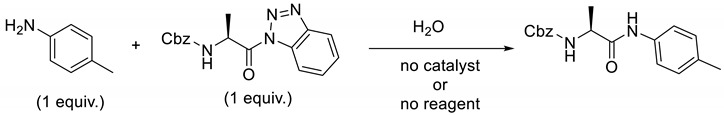			
**Entry**	**Reaction Temp. (°C)**	**Reaction Time**	**Yield ^a^ (%)**
1	20 (Room temp.)	1 h	74
2	20 (Room temp.)	2 h	86
3	50 (Conv.)	30 min	77
4	50 (Conv.)	1 h	82
5	70 (MW)	15 min	96

^a^ Isolated yield.

**Table 2:** Preparation of *N*-acylated amines.

Entry	R-NH_2_	Product	Yield (%)	Mp (°C)
1	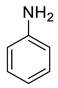	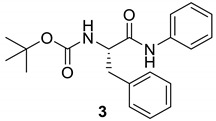	94	137–139
2	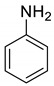	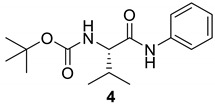	93	123–125
3	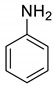	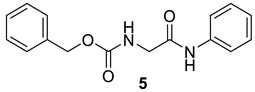	95	145–146
4	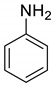	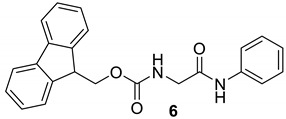	91	165–167
5	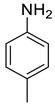	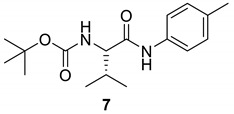	95	118–120
6	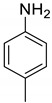	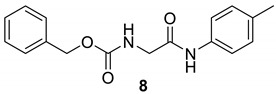	88	152–154
7	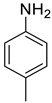	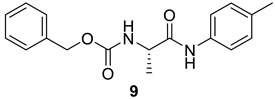	96	158–160
8	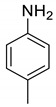	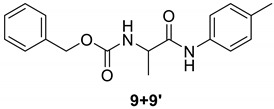	92	152–154
9	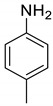	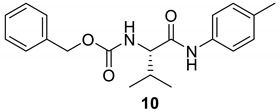	97	183–185
10	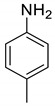	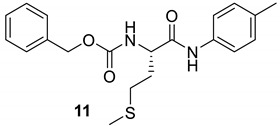	79	185–187
11	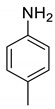	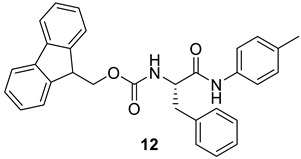	94	193–195
12	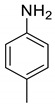	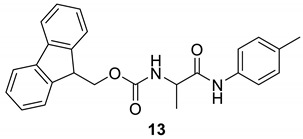	75	172–174
13	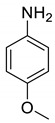	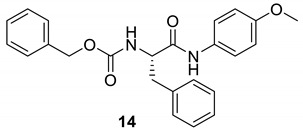	80	164–166
14	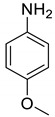	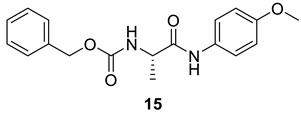	85	160–162
15	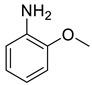	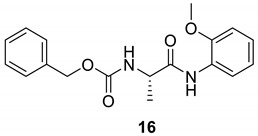	98	185–187
16	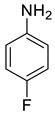	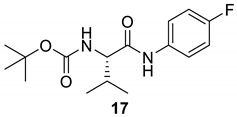	94	138–140
17	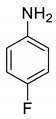	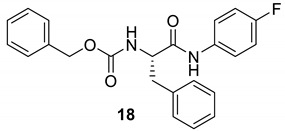	77	191–193
18	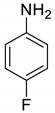	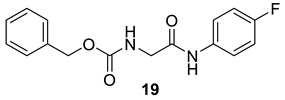	84	174–176
19	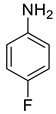	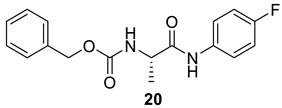	90	192–194
20	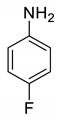	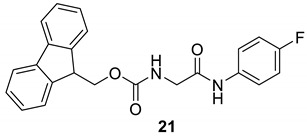	90	142–143
21	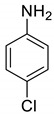	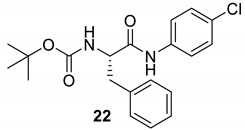	98	152–154
22	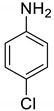	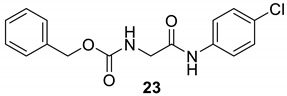	95	160–162
23	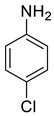	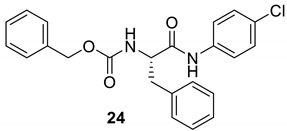	90	182–184
24	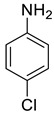	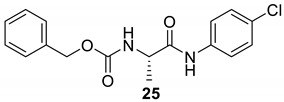	73	163–165
25	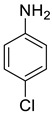	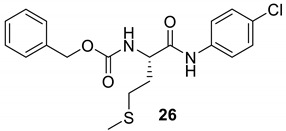	82	150–152
26	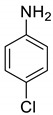	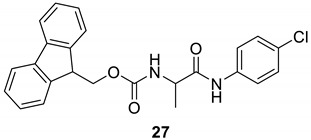	90	197–199
27	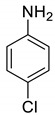	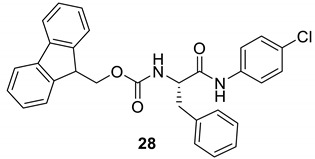	98	210–212
28	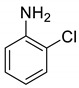	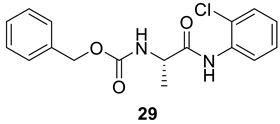	70	162–164
29	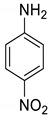	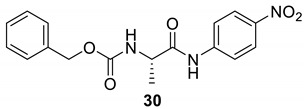	70	95–97
30	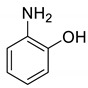	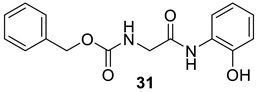	90	180–182
31	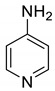	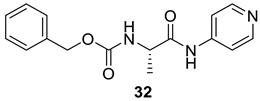	89	152–154
32	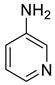	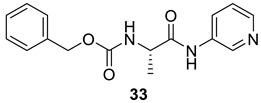	97	155–157
33	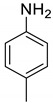	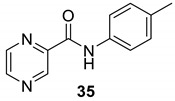	71	138–140
34	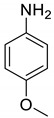	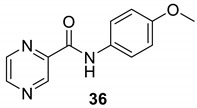	81	147–149
35	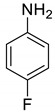	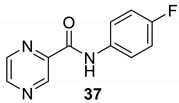	98	155–157
36	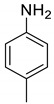	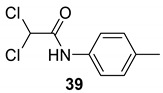	85	157–159
37	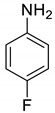	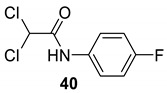	80	130–132
38	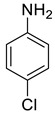	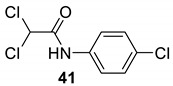	80	143–145

## Supplementary Materials

The following are available online at https://www.mdpi.com/1420-3049/25/11/2501/s1, general experimental data and NMR spectra.
