# Supplementary material for: An Efficient Greener Approach for N-acylation of Amines in Water Using Benzotriazole Chemistry
Source: Molecules. 2020 May 28;25(11):2501. doi: 10.3390/molecules25112501 (PMC7321353; doi:10.3390/molecules25112501)
Supplement: Supplementary file 1 [file molecules-25-02501-s001.pdf]

molecules

Supplementary Information

An efficient greener approach for N-acylation of amines in water using benzotriazole chemistry

Tarek S. Ibrahim [1,2], Israa A. Seliem [2,3], Siva S. Panda [3,*], Amany M. M. Al-Mahmoudy [2], Zakaria K. M. Abdel-samii [2], Nabil A. Alhakamy [4], Hany Z. Asfour [5] and Mohamed Elagawany [6]

1 Department of Pharmaceutical Chemistry, Faculty of Pharmacy, King Abdulaziz University, Jeddah, 21589, Saudi Arabia. tmabrahem@kau.edu.sa
2 Department of Pharmaceutical Organic Chemistry, Faculty of Pharmacy, Zagazig University, Zagazig, 44519, Egypt. isliem@augusta.edu (I.A.S.); amanysinger77@gmail.com (A.M.M.A-M.); zakariaabdelsamii@yahoo.com (Z.K.M.A.)
3 Department of Chemistry & Physics, Augusta University, Augusta, GA 30912, USA
4 Department of Pharmaceutics, Faculty of Pharmacy, King Abdulaziz University, Jeddah 21589, Saudi Arabia.
5 Department of Medical Microbiology and Parasitology, Faculty of Medicine, King Abdulaziz University, Jeddah 21589, Saudi Arabia
6 Department of Pharmaceutical Chemistry, faculty of pharmacy, Damanhour University, Damanhour, Egypt. alfath_tours@yahoo.com
* Correspondence: sspanda12@gmail.com, sipanda@augusta.edu; Tel: +1-706-667-4022; fax: +1-706-667-4519

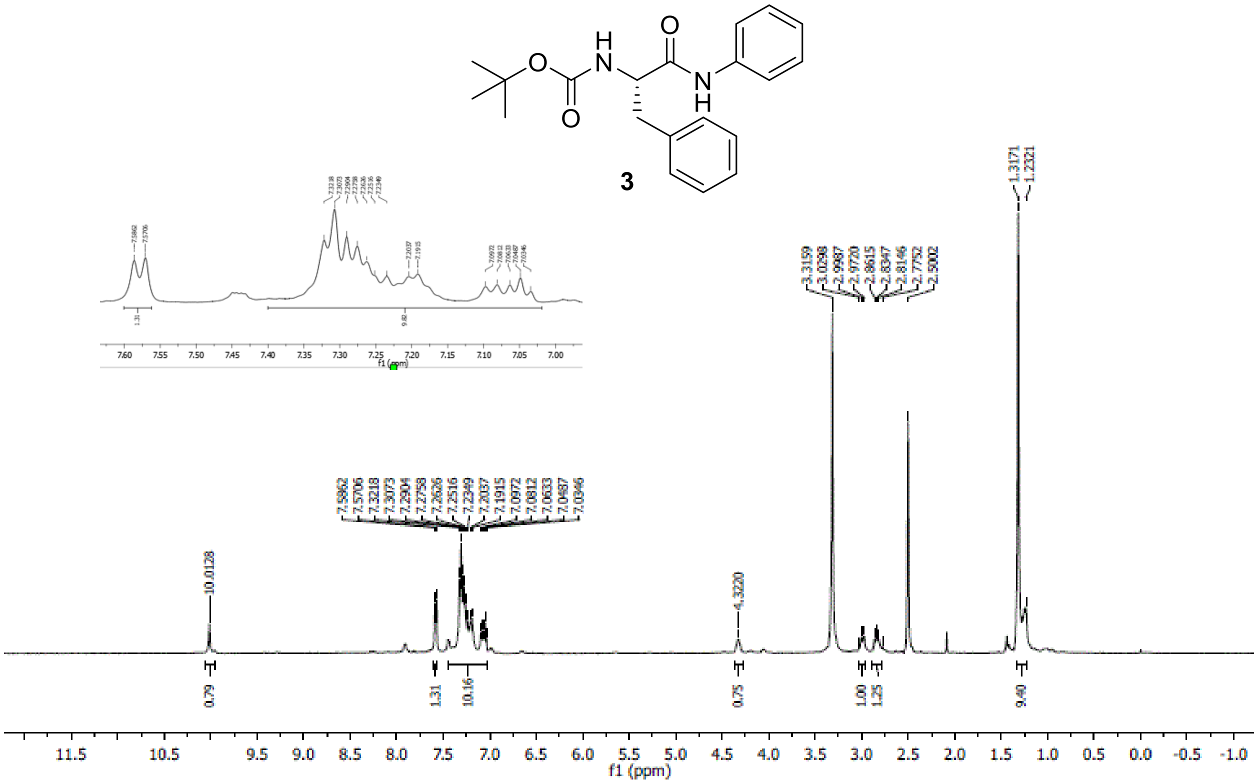
O
H
N
O
N
H
O
3
10.0128
7.5862
7.5706
7.3218
7.3073
7.2904
7.2758
7.2626
7.2516
7.2349
7.2037
7.1915
7.0972
7.0812
7.0633
7.0487
7.0346
4.3220
3.3159
3.0298
2.9987
2.9720
2.8615
2.8347
2.8146
2.7752
2.5002
1.3171
1.2321
0.79
1.31
10.16
0.75
1.00
1.25
9.40
11.5
10.5
9.5
9.0
8.5
8.0
7.5
7.0
6.5
6.0
5.5
5.0
4.5
4.0
3.5
3.0
2.5
2.0
1.5
1.0
0.5
0.0
-0.5
-1.0
f1 (ppm)

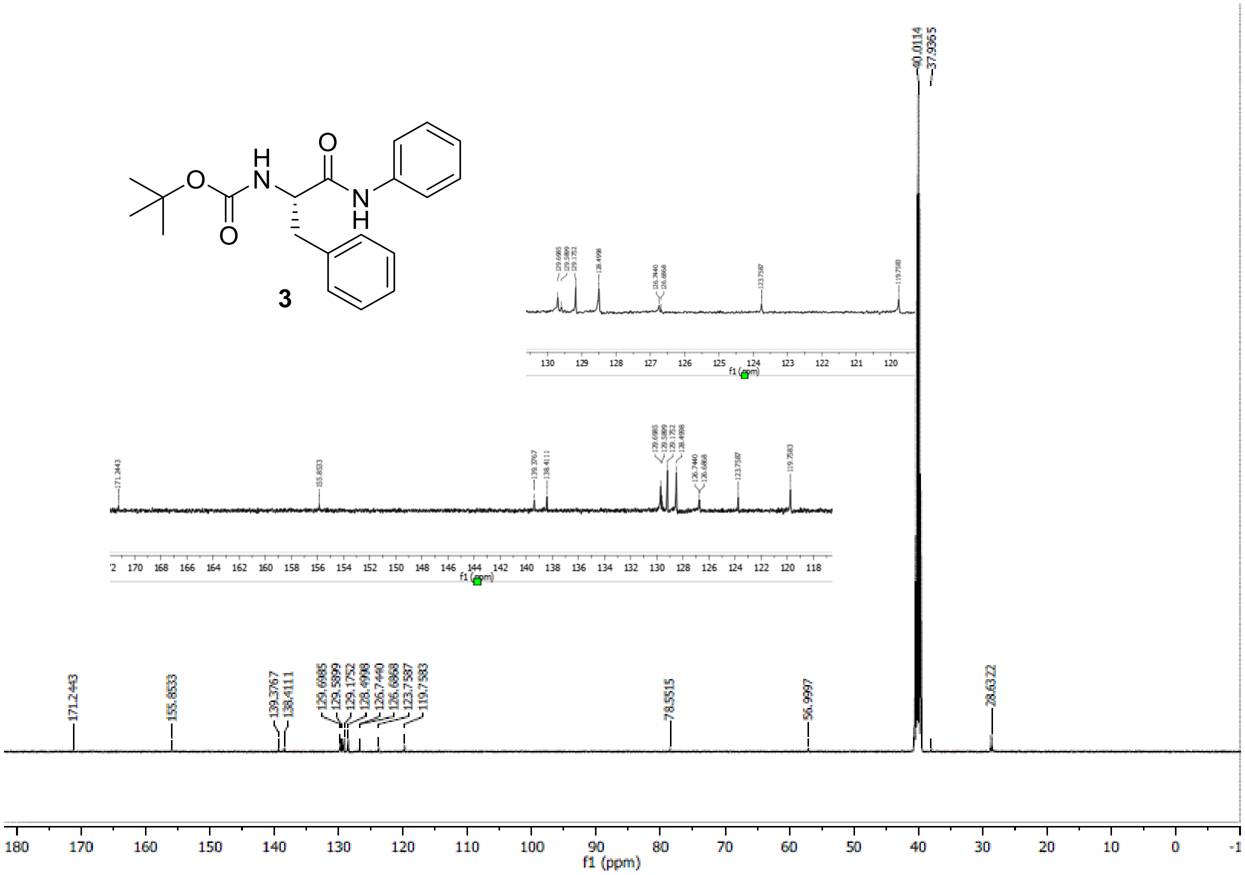
40.0114
37.9365
O
H
N
O
N
H
O
3
171.2443
155.8533
139.3767
138.4111
129.6985
129.5899
129.1752
128.4998
126.7440
126.6868
123.7587
119.7583
78.5515
56.9997
28.6322
180
170
160
150
140
130
120
110
100
90
80
70
60
50
40
30
20
10
0
-1
f1 (ppm)

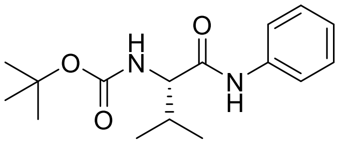

4

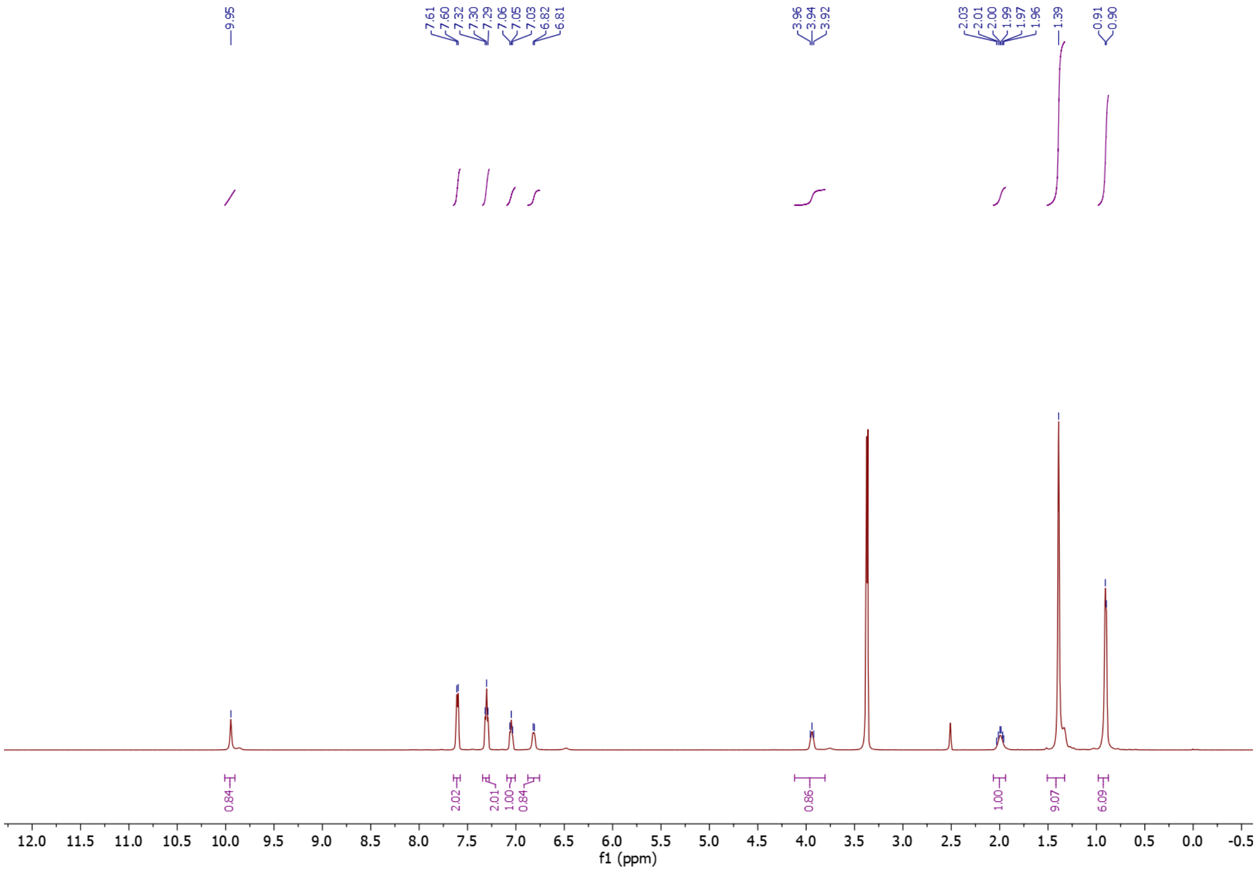

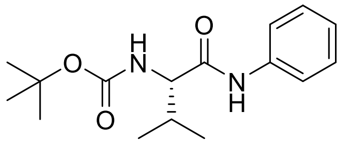

4

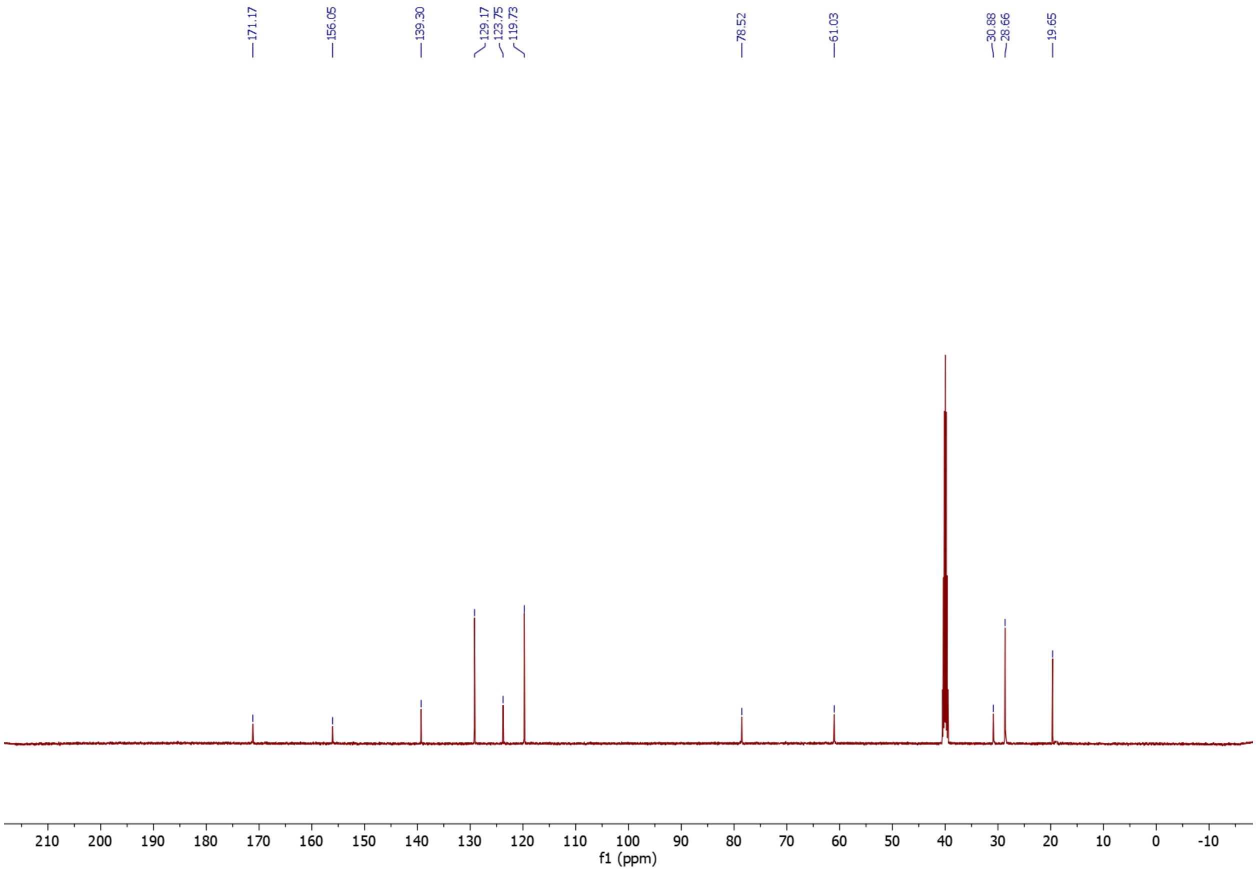
171.17
156.05
139.30
129.17
123.75
119.73
78.52
61.03
30.88
28.66
19.65
210
200
190
180
170
160
150
140
130
120
110
100
90
80
70
60
50
40
30
20
10
0
-10
f1 (ppm)

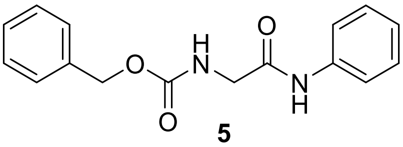
O
H
N
O
N
H
O

5

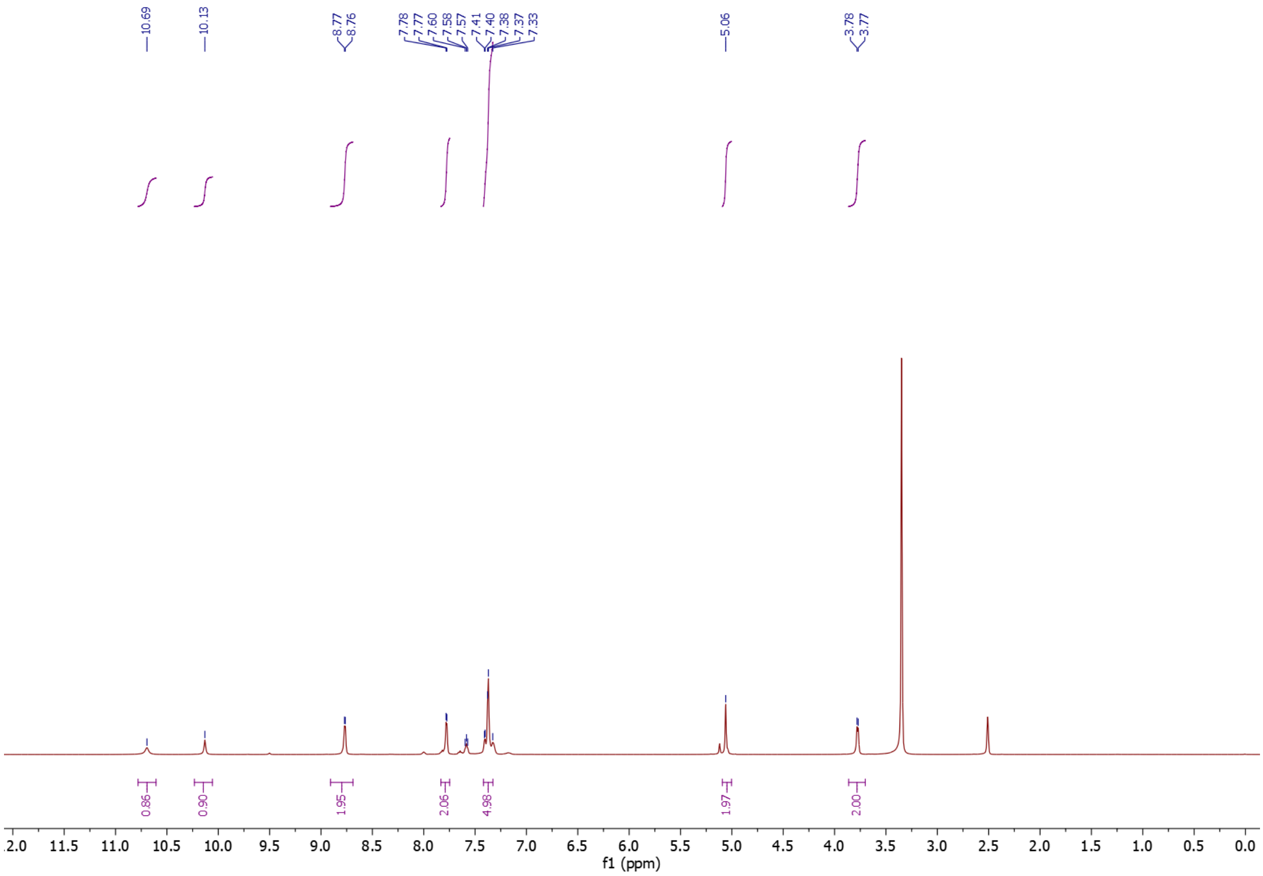
10.69
10.13
8.77
8.76
7.78
7.77
7.60
7.58
7.57
7.41
7.40
7.38
7.37
7.33
5.06
3.78
3.77
0.86
0.90
1.95
2.06
4.98
1.97
2.00
.2.0 11.5 11.0 10.5 10.0 9.5 9.0 8.5 8.0 7.5 7.0 6.5 6.0 5.5 5.0 4.5 4.0 3.5 3.0 2.5 2.0 1.5 1.0 0.5 0.0
f1 (ppm)

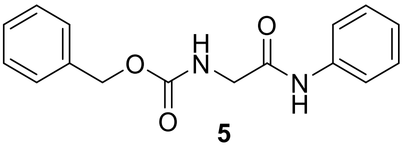

5

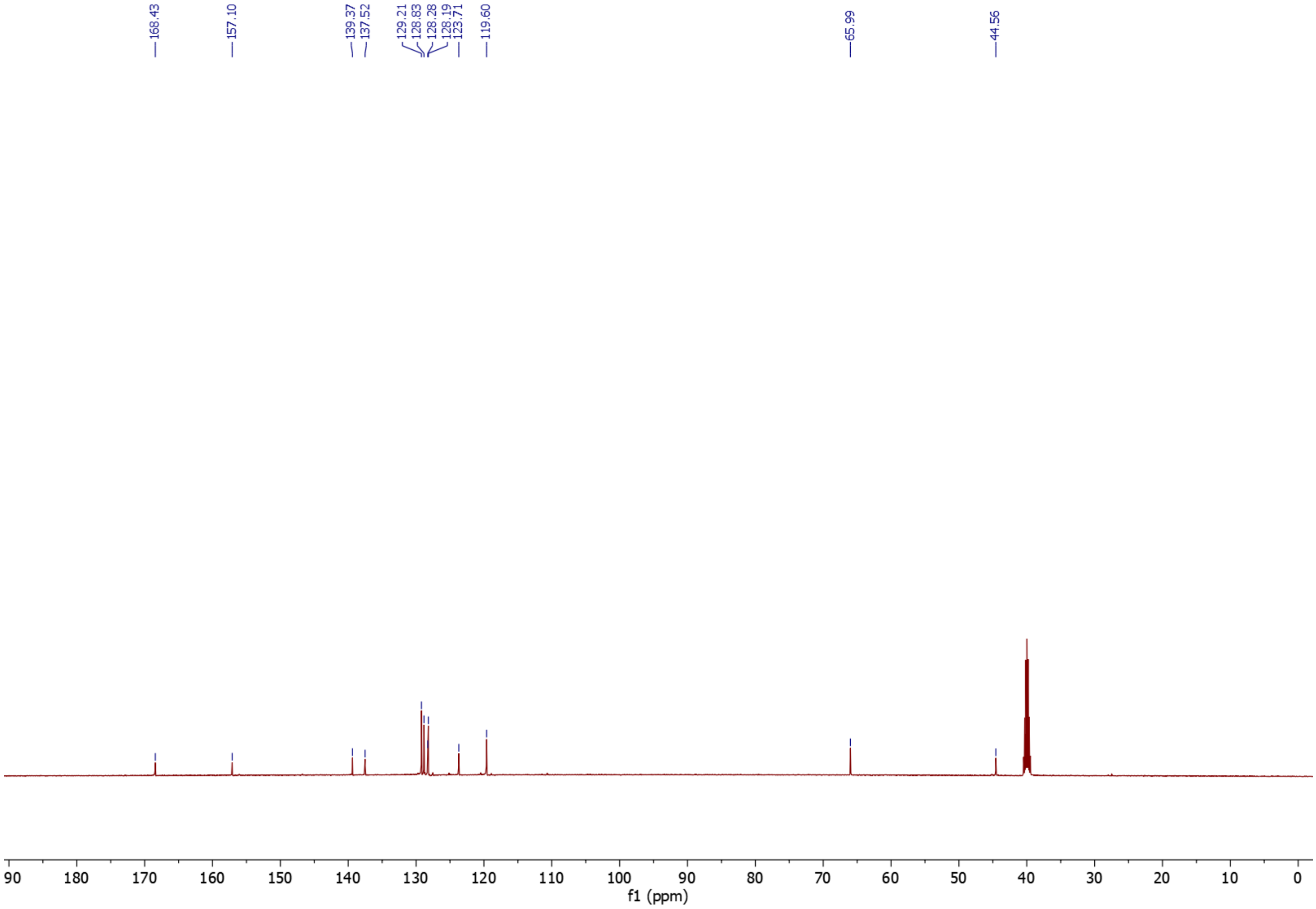
168.43
157.10
139.37
137.52
129.21
128.83
128.28
128.19
123.71
119.60
65.99
44.56
f1 (ppm)

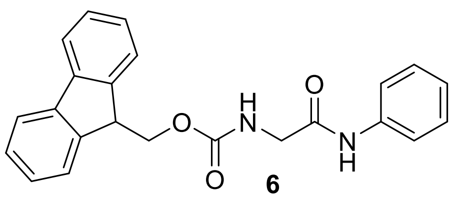

6

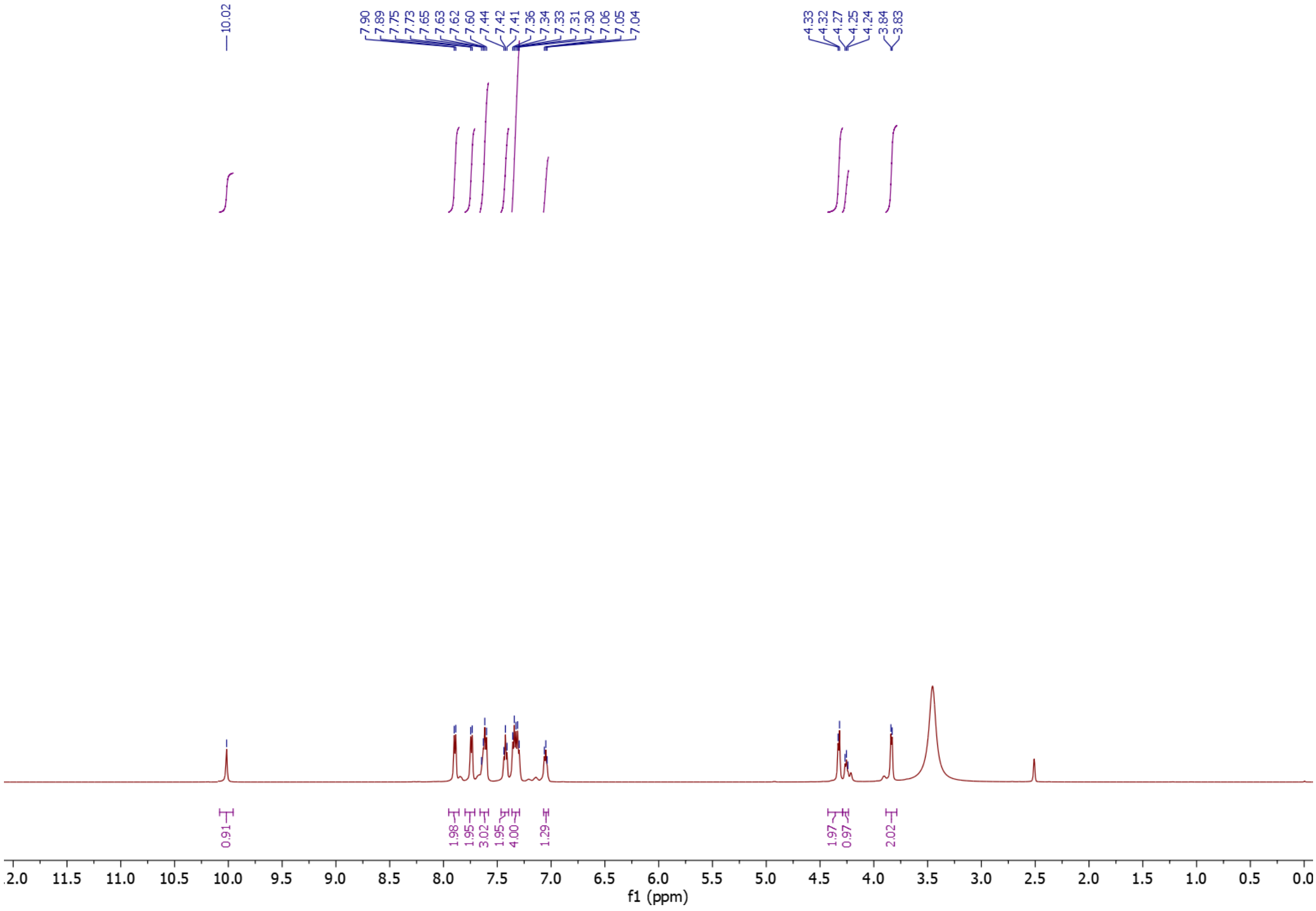
10.02
7.90
7.89
7.75
7.73
7.65
7.63
7.62
7.60
7.44
7.42
7.41
7.36
7.34
7.33
7.31
7.30
7.06
7.05
7.04
4.33
4.32
4.27
4.25
4.24
3.84
3.83
0.91
1.98
1.95
3.02
1.95
4.00
1.29
1.97
0.97
2.02
f1 (ppm)

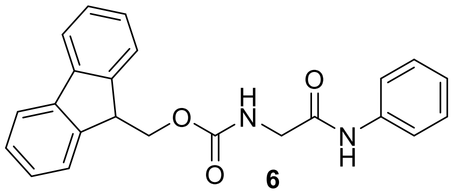
O
H
N
O
N
H
O

6

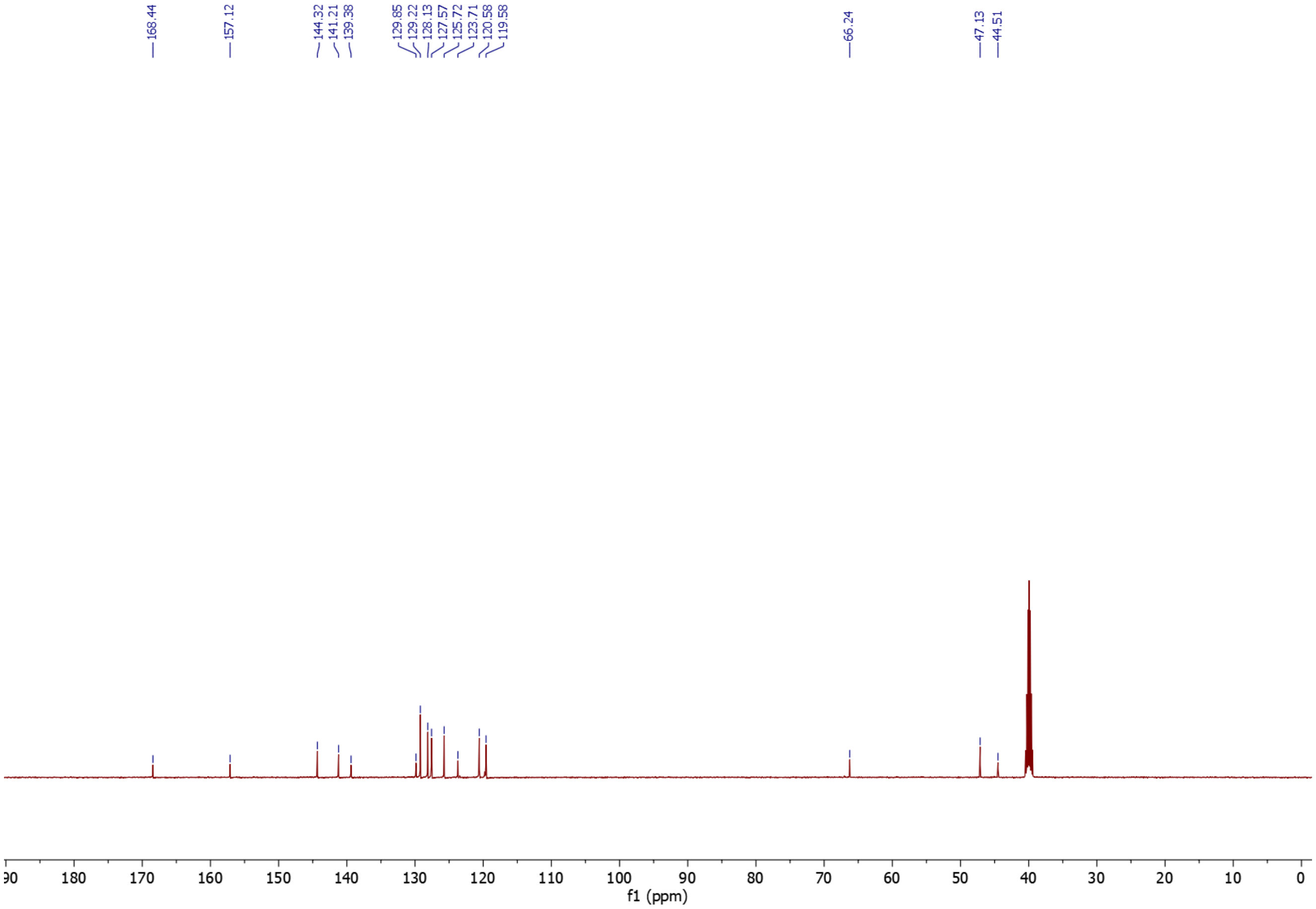
168.44
157.12
144.32
141.21
139.38
129.85
129.22
128.13
127.57
125.72
123.71
120.58
119.58
66.24
47.13
44.51
f1 (ppm)

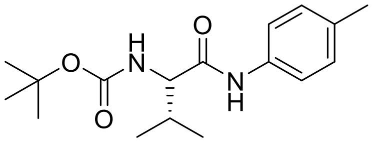

7

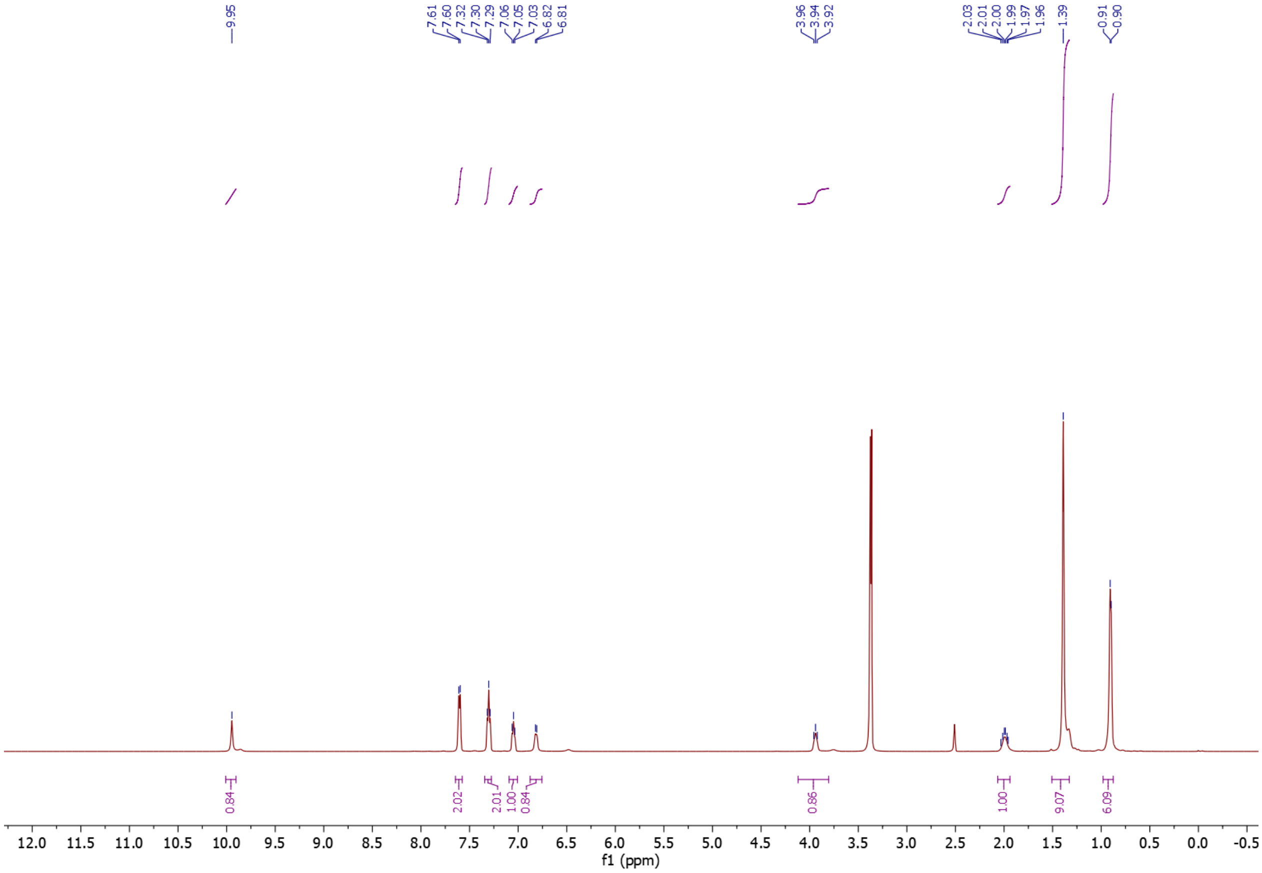
9.95
7.61
7.60
7.32
7.30
7.29
7.06
7.05
7.03
6.82
6.81
3.96
3.94
3.92
2.03
2.01
2.00
1.99
1.97
1.96
1.39
0.91
0.90
0.84
2.02
2.01
1.00
0.84
0.86
1.00
9.07
6.09
12.0 11.5 11.0 10.5 10.0 9.5 9.0 8.5 8.0 7.5 7.0 6.5 6.0 5.5 5.0 4.5 4.0 3.5 3.0 2.5 2.0 1.5 1.0 0.5 0.0 -0.5
f1 (ppm)

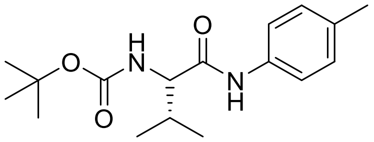
H
N
O
O
O
N
H

7

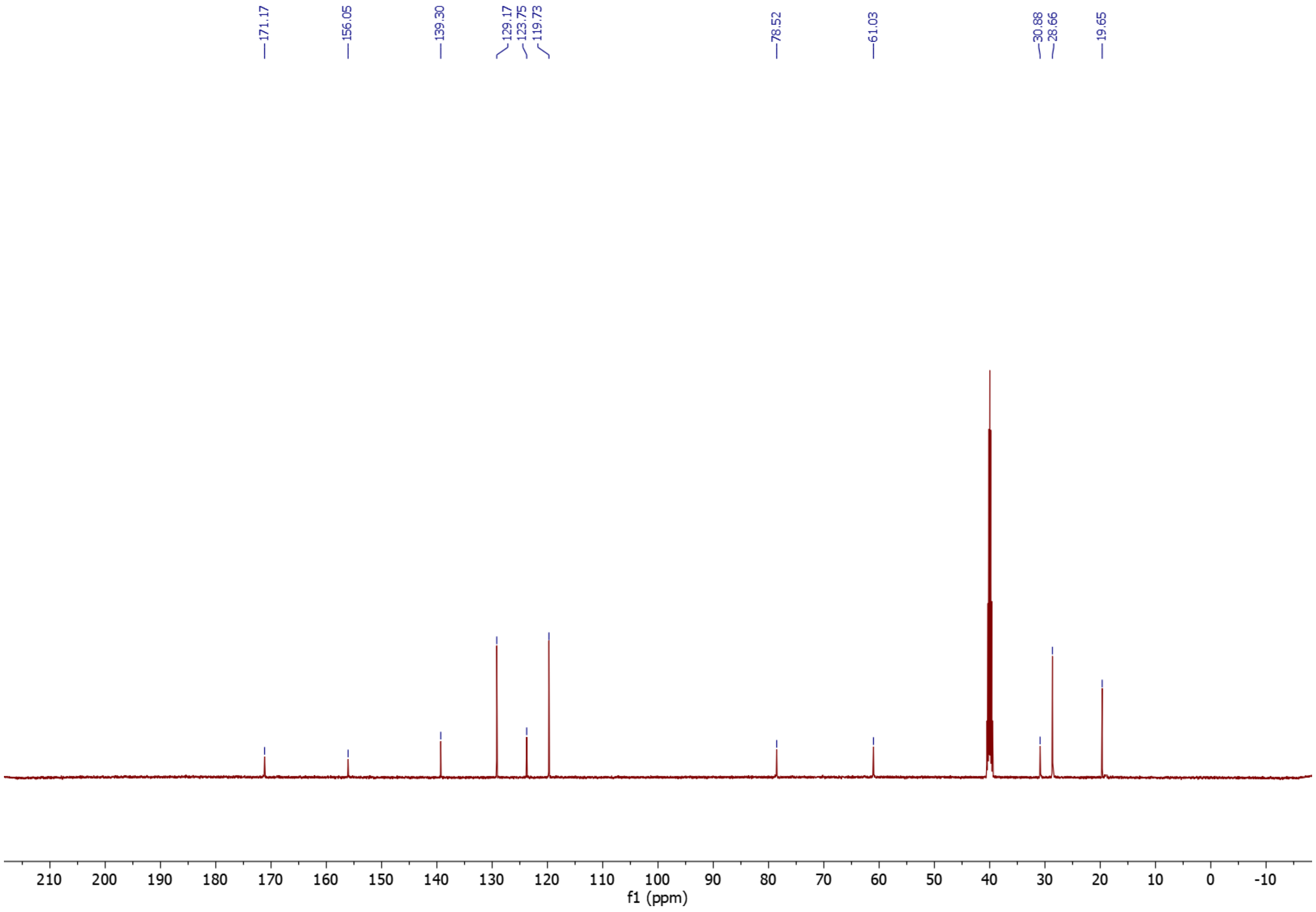
171.17
156.05
139.30
129.17
123.75
119.73
78.52
61.03
30.88
28.66
19.65
210 200 190 180 170 160 150 140 130 120 110 100 90 80 70 60 50 40 30 20 10 0 -10
f1 (ppm)

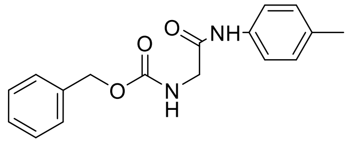

8

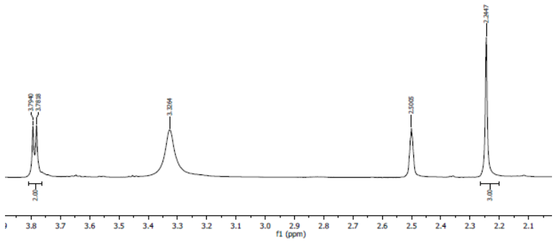

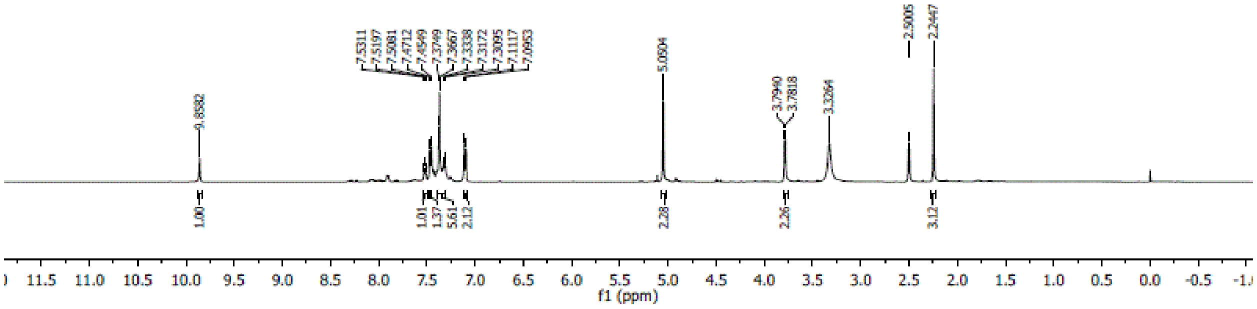
7.5311
7.5197
7.5081
7.4712
7.4549
7.3749
7.3667
7.3338
7.3172
7.3095
7.1117
7.0953
9.8582
5.0504
3.7940
3.7818
3.3264
2.5005
2.2447
1.00
1.01
1.37
5.61
2.12
2.28
2.26
3.12
f1 (ppm)

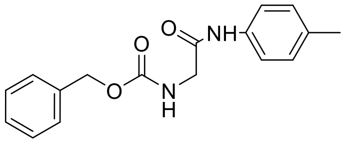

8

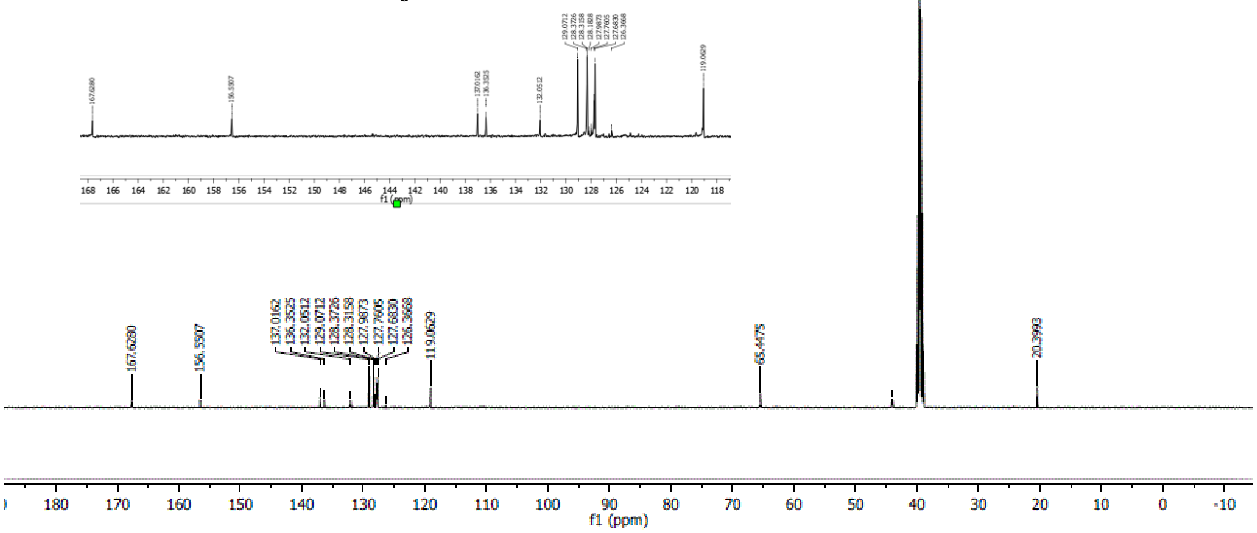
43.9900
39.4999
167.6280
156.5507
137.0162
136.3525
132.0512
129.0712
128.3726
128.3158
127.9873
127.7605
127.6830
126.3668
119.0629
65.4475
20.3993
f1 (ppm)

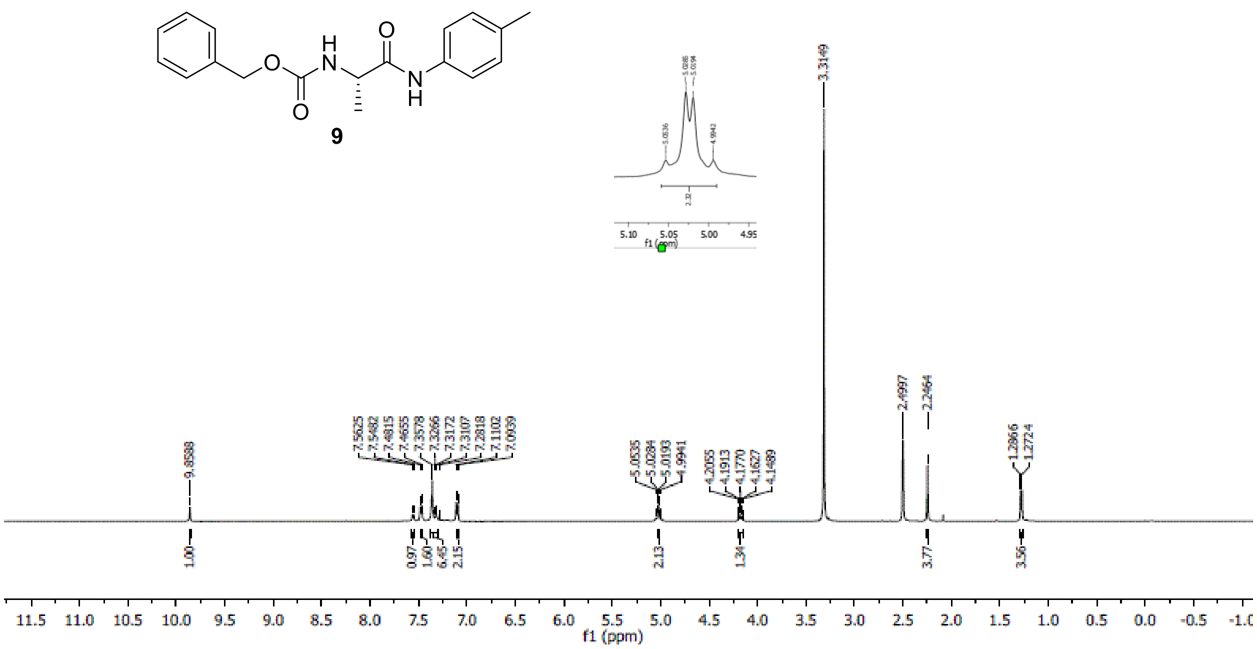
9
3.3149
2.4997
2.2464
9.8588
7.5625
7.5482
7.4815
7.4655
7.3578
7.3266
7.3172
7.3107
7.2818
7.1102
7.0939
5.0535
5.0284
5.0193
4.9941
4.2055
4.1913
4.1770
4.1627
4.1489
1.2866
1.2724
1.00
0.97
1.60
6.45
2.15
2.13
1.34
3.77
3.56
f1 (ppm)

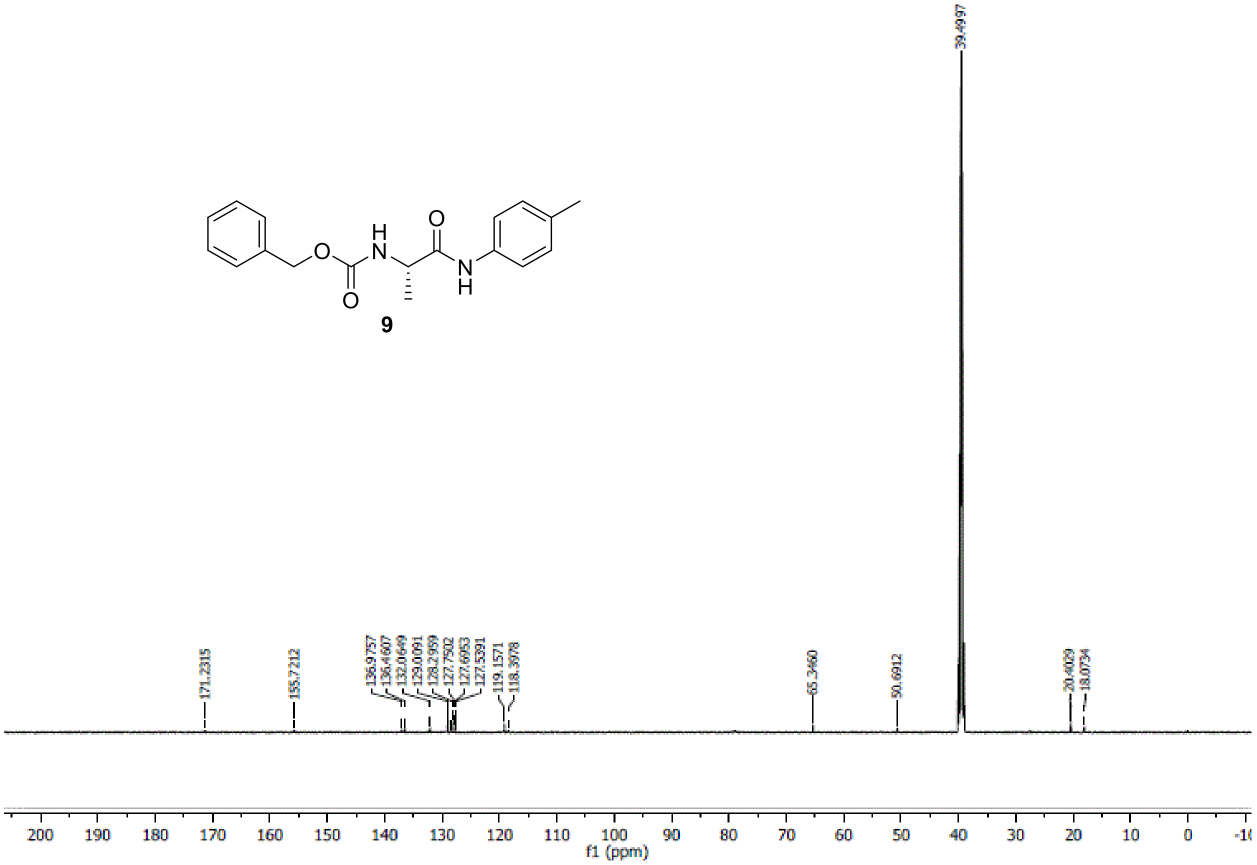
9
39.4997
171.2315
155.7212
136.9757
136.4607
132.0649
129.0091
128.2959
127.7502
127.6953
127.5391
119.1571
118.3978
65.3460
50.6912
20.4029
18.0734
f1 (ppm)

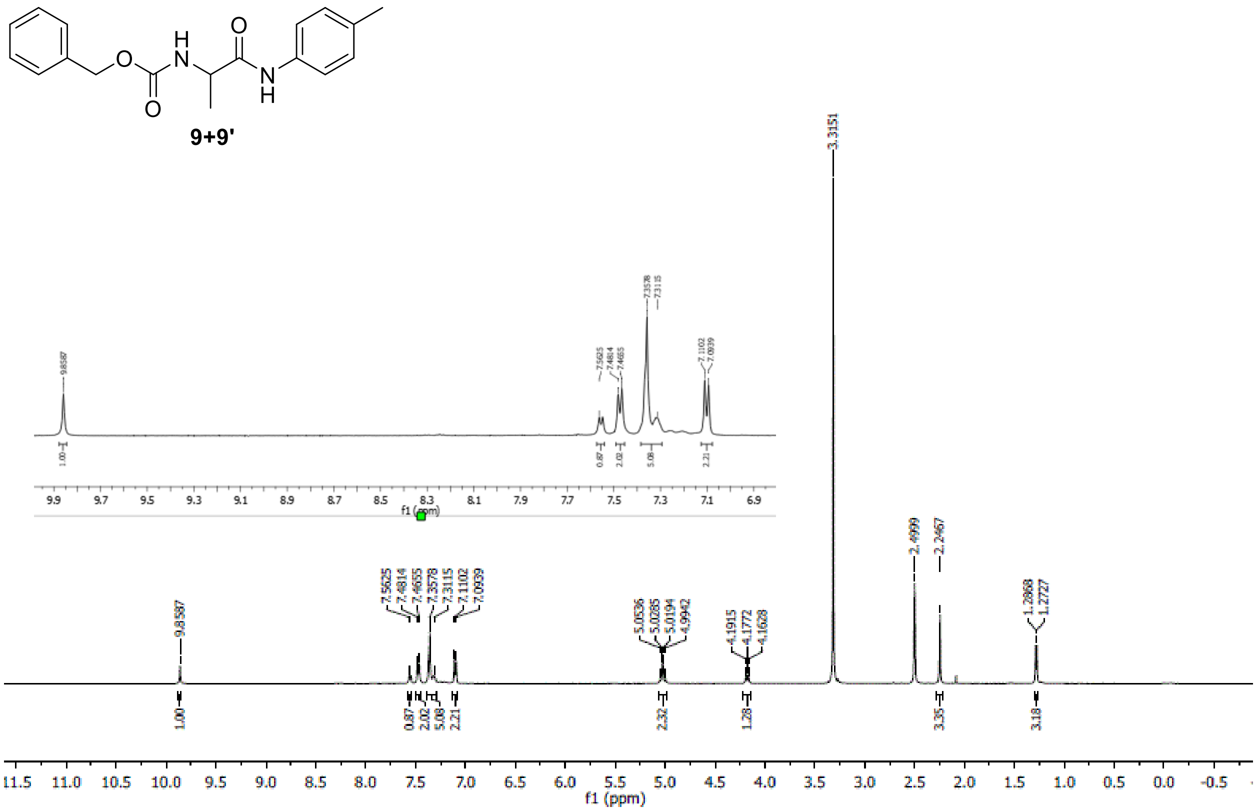

9+9'
9.8587
7.5625 7.4814 7.4655 7.3578 7.3115 7.1102 7.0939
5.0536 5.0285 5.0194 4.9942
4.1915 4.1772 4.1628
3.3151
2.4999
2.2467
1.2868 1.2727
1.00
0.87 2.02 5.08 2.21
2.32
1.28
3.35
3.18
f1 (ppm)

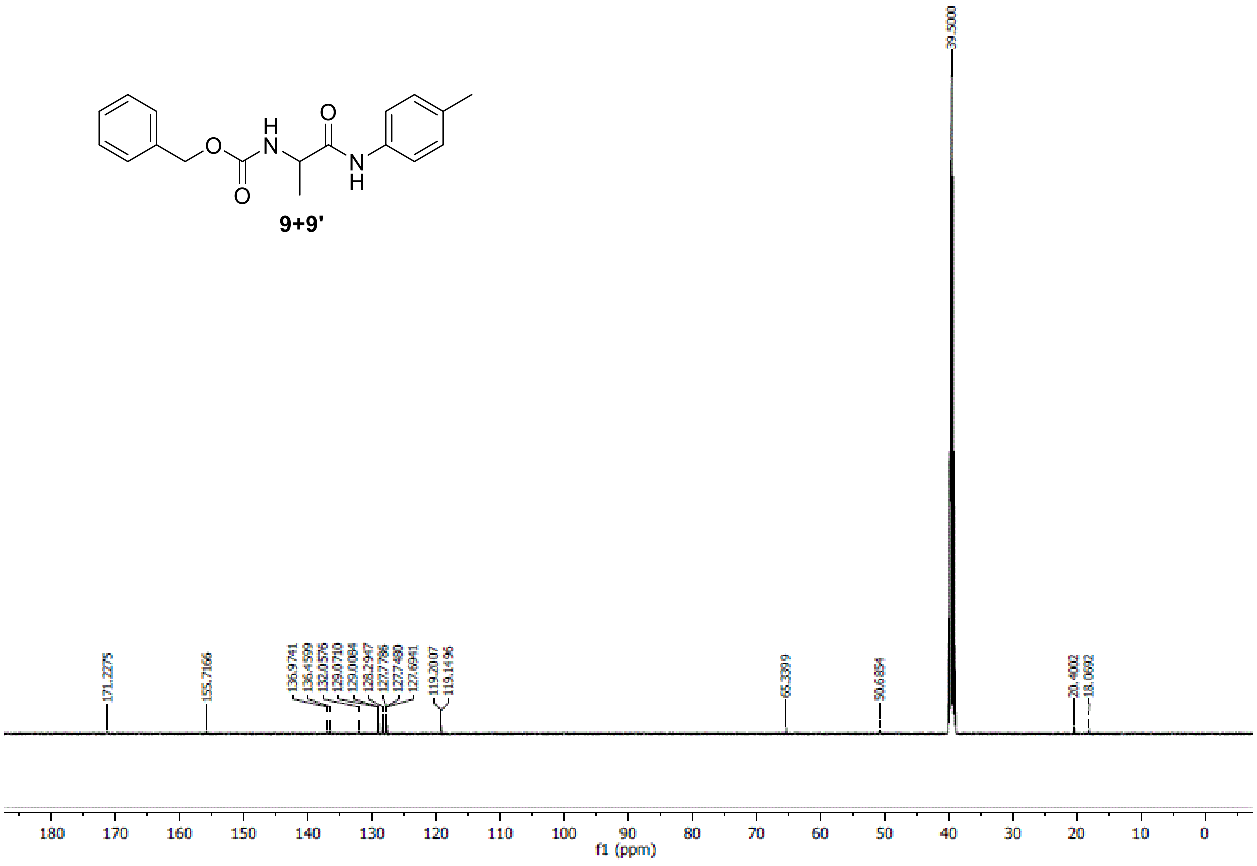

9+9'
39.5000
171.2275
155.7166
136.9741 136.4599 132.0576 129.0710 129.0084 128.2947 127.7786 127.7480 127.6941 119.2007 119.1496
65.3399
50.6854
20.4002 18.0692
f1 (ppm)

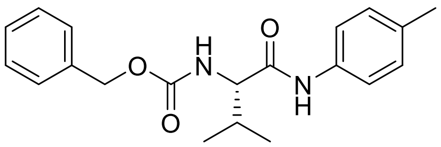
H
N
O
O
O
N
H

10

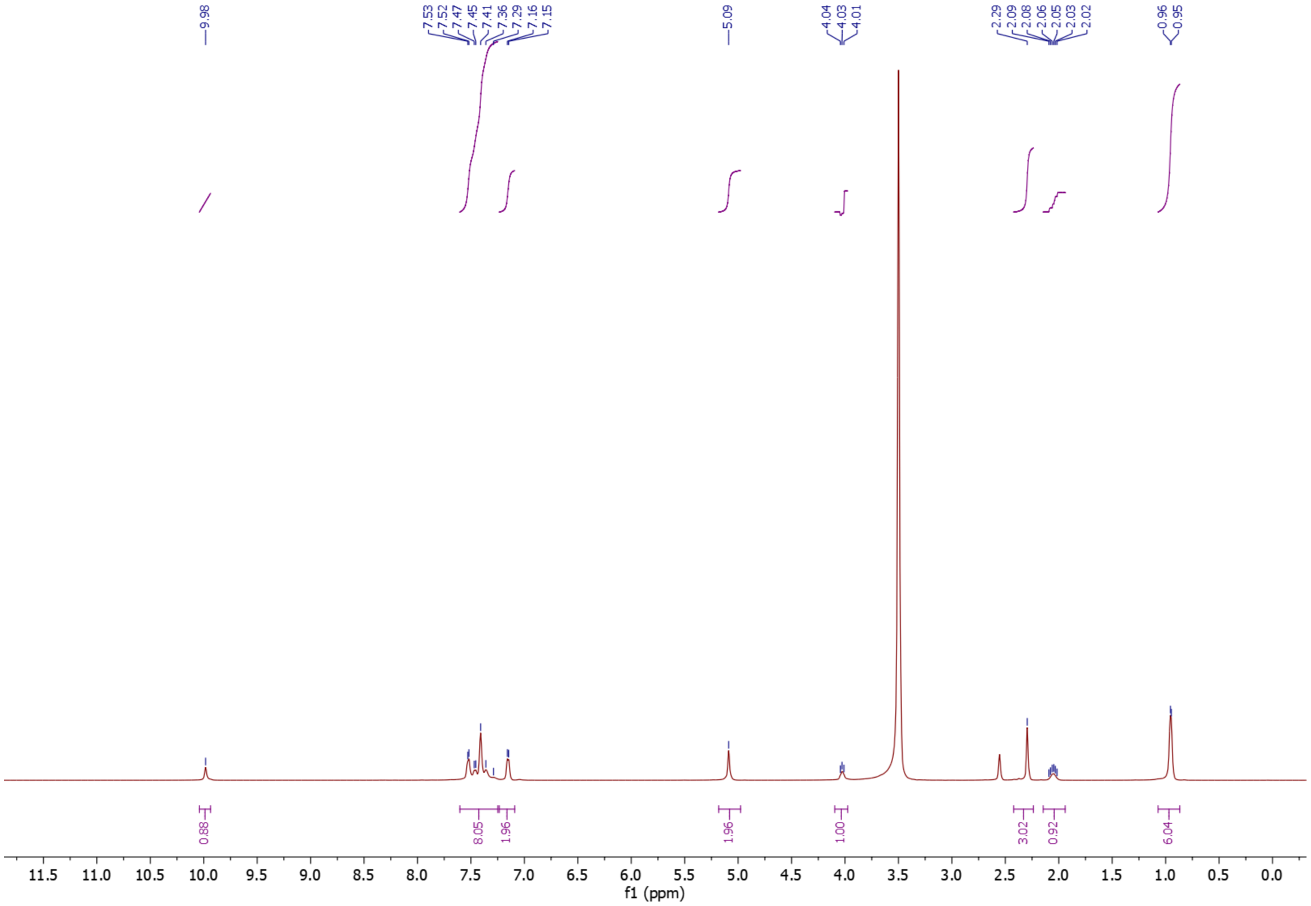
9.98
7.53
7.52
7.47
7.45
7.41
7.36
7.29
7.16
7.15
5.09
4.04
4.03
4.01
2.29
2.09
2.08
2.06
2.05
2.03
2.02
0.96
0.95
0.88
8.05
1.96
1.96
1.00
3.02
0.92
6.04
11.5 11.0 10.5 10.0 9.5 9.0 8.5 8.0 7.5 7.0 6.5 6.0 5.5 5.0 4.5 4.0 3.5 3.0 2.5 2.0 1.5 1.0 0.5 0.0
f1 (ppm)

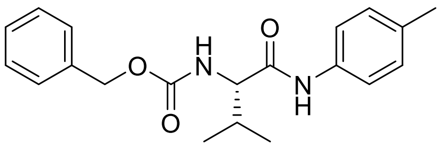

10

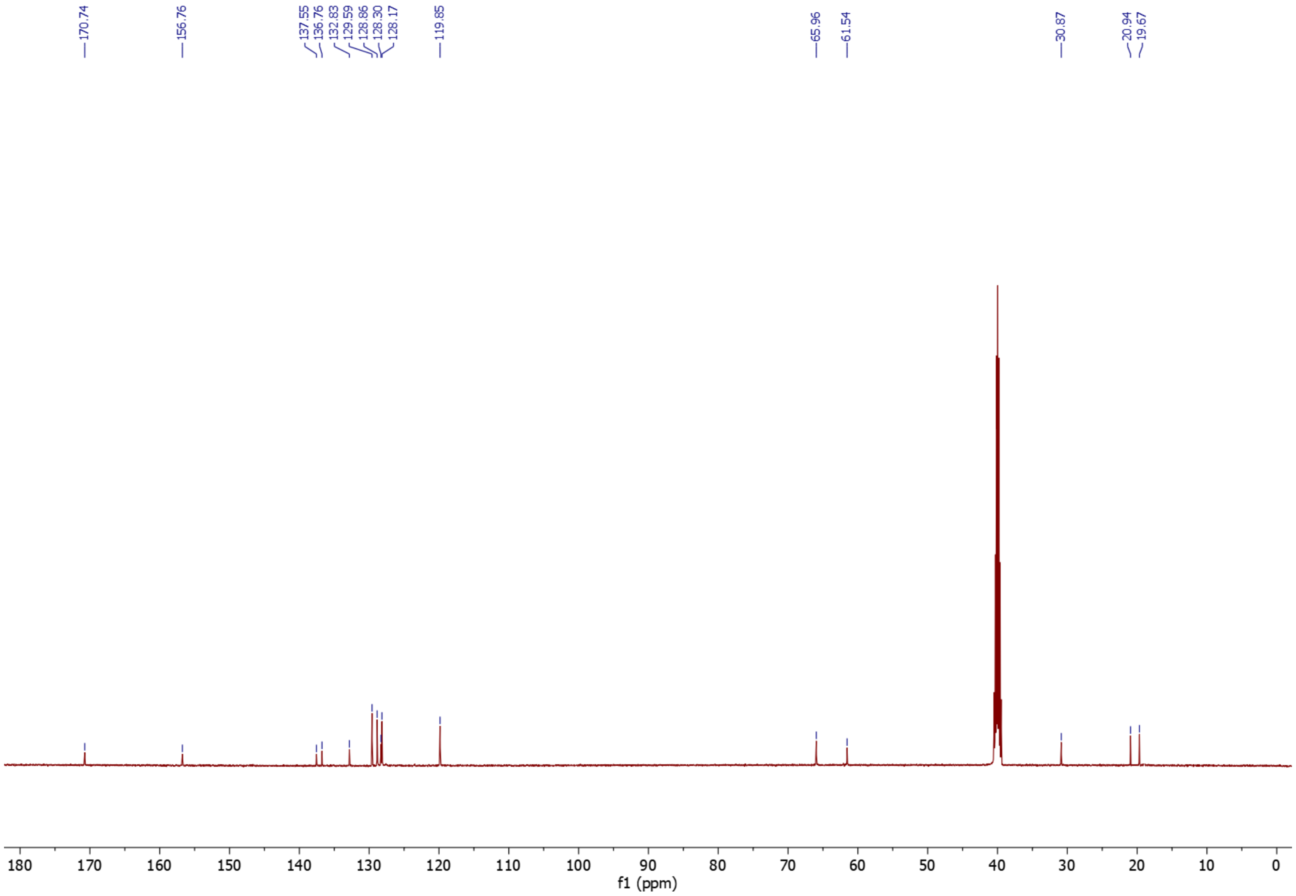
170.74
156.76
137.55
136.76
132.83
129.59
128.86
128.30
128.17
119.85
65.96
61.54
30.87
20.94
19.67
180 170 160 150 140 130 120 110 100 90 80 70 60 50 40 30 20 10 0
f1 (ppm)

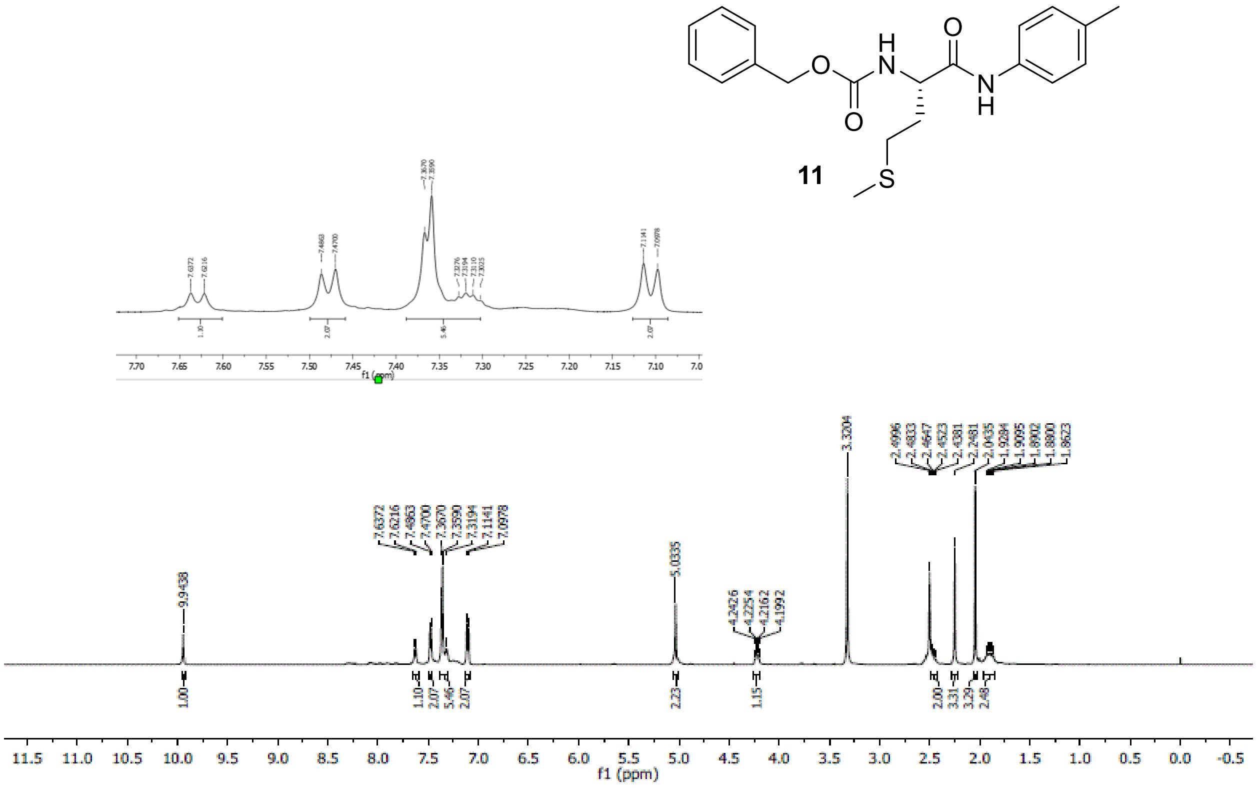

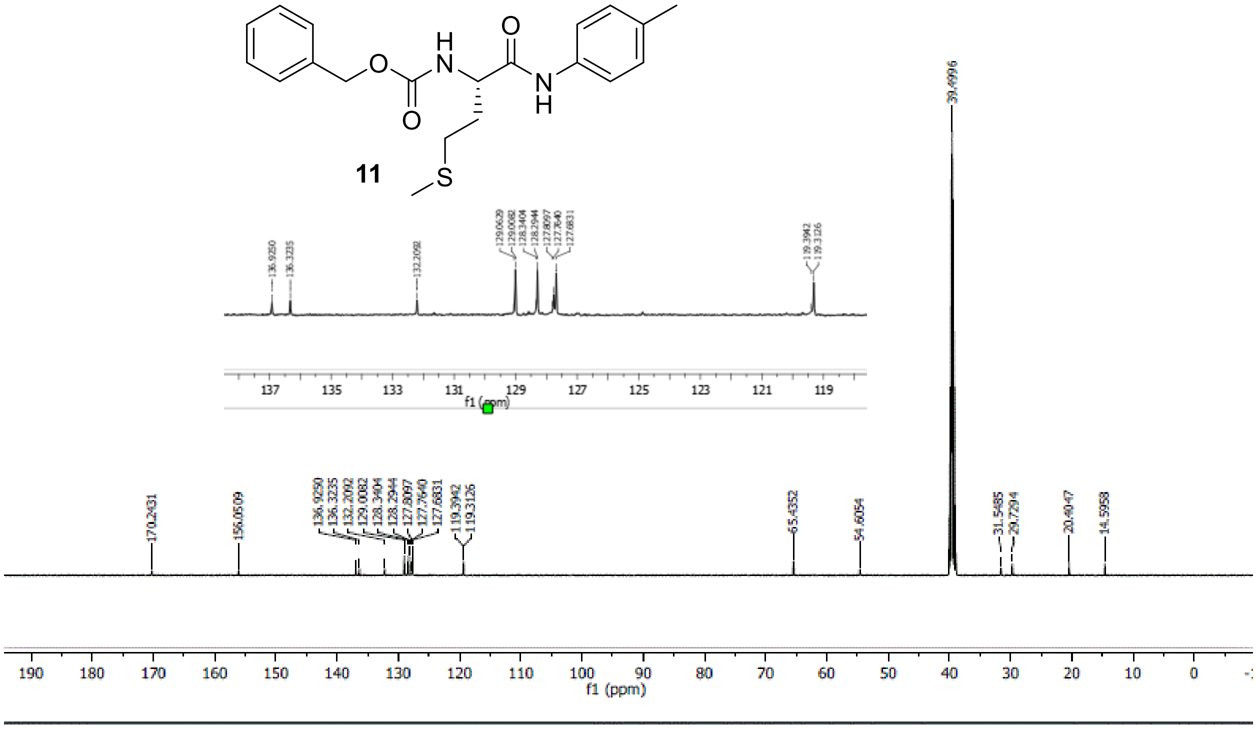

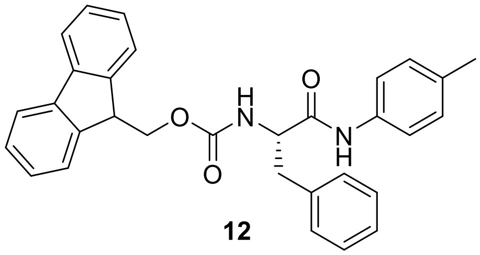

12

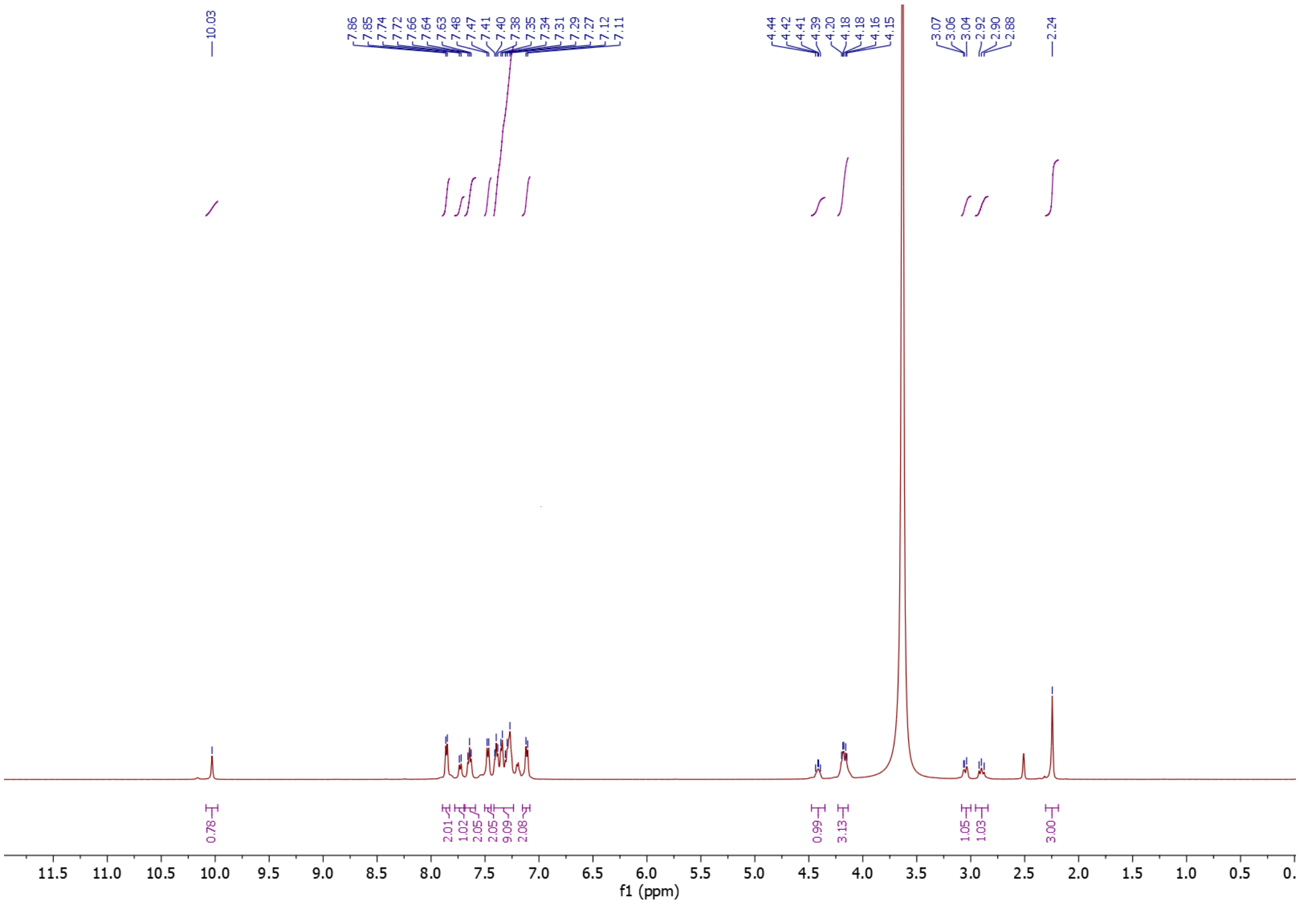

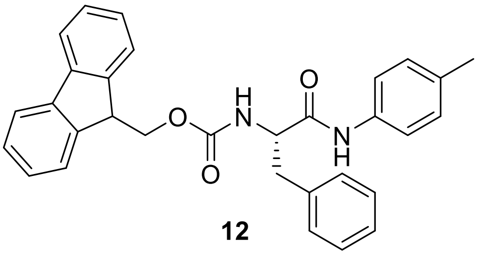

12

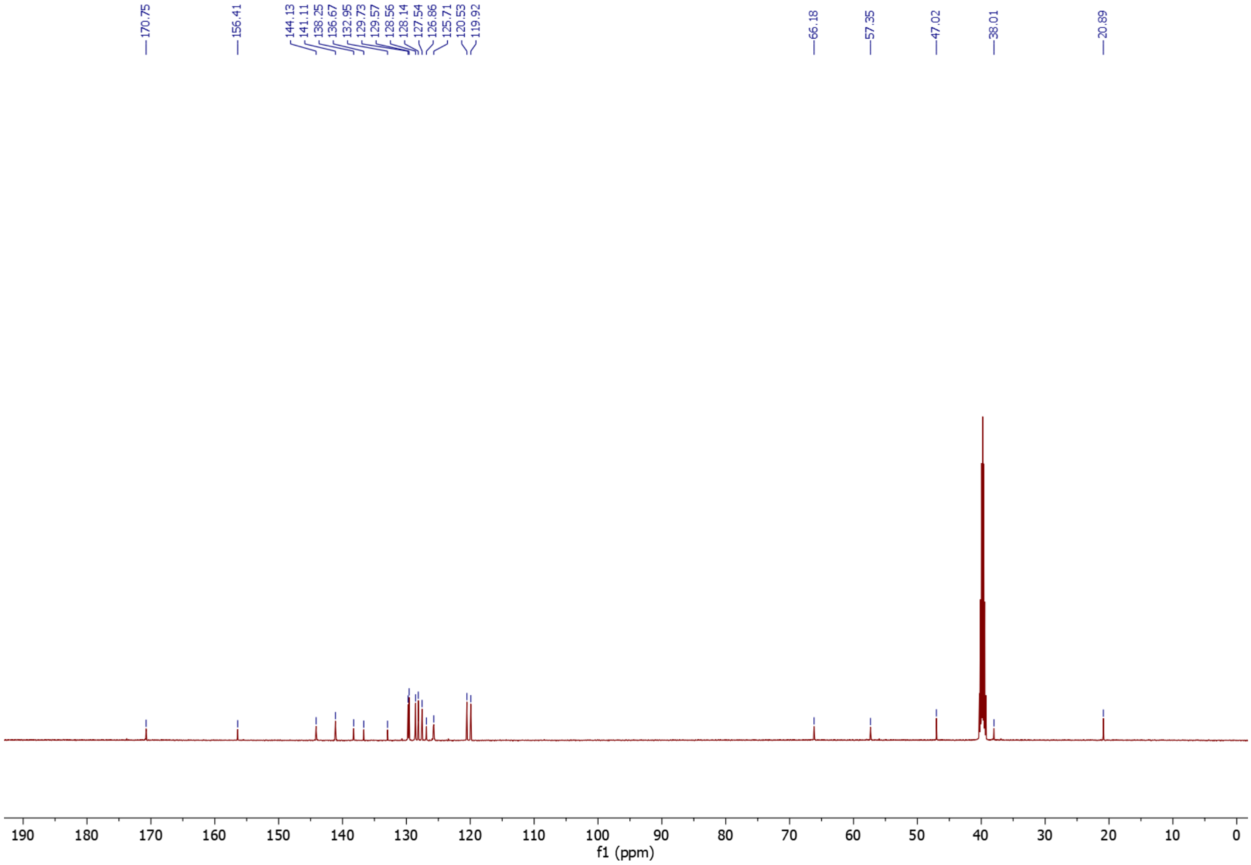
170.75
156.41
144.13
141.11
138.25
136.67
132.95
129.73
129.57
128.56
128.14
127.54
126.86
125.71
120.53
119.92
66.18
57.35
47.02
38.01
20.89
190 180 170 160 150 140 130 120 110 100 90 80 70 60 50 40 30 20 10 0
f1 (ppm)

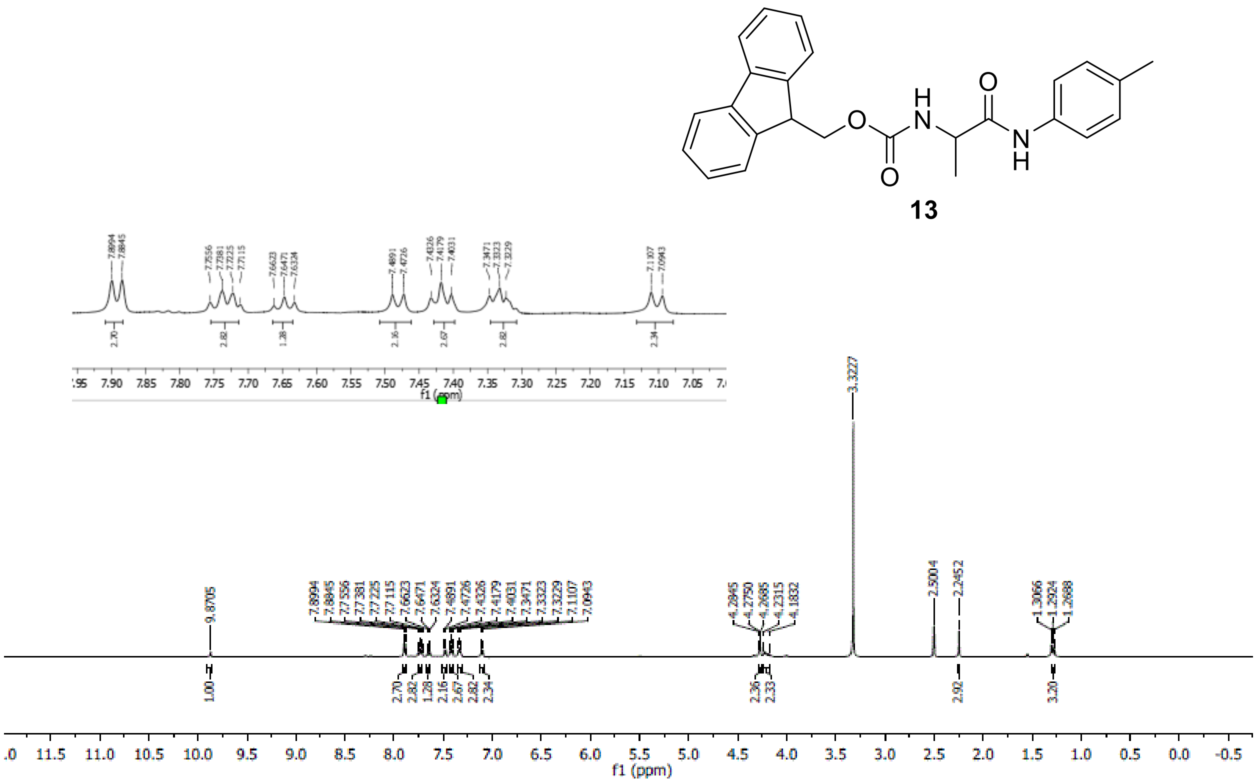
13

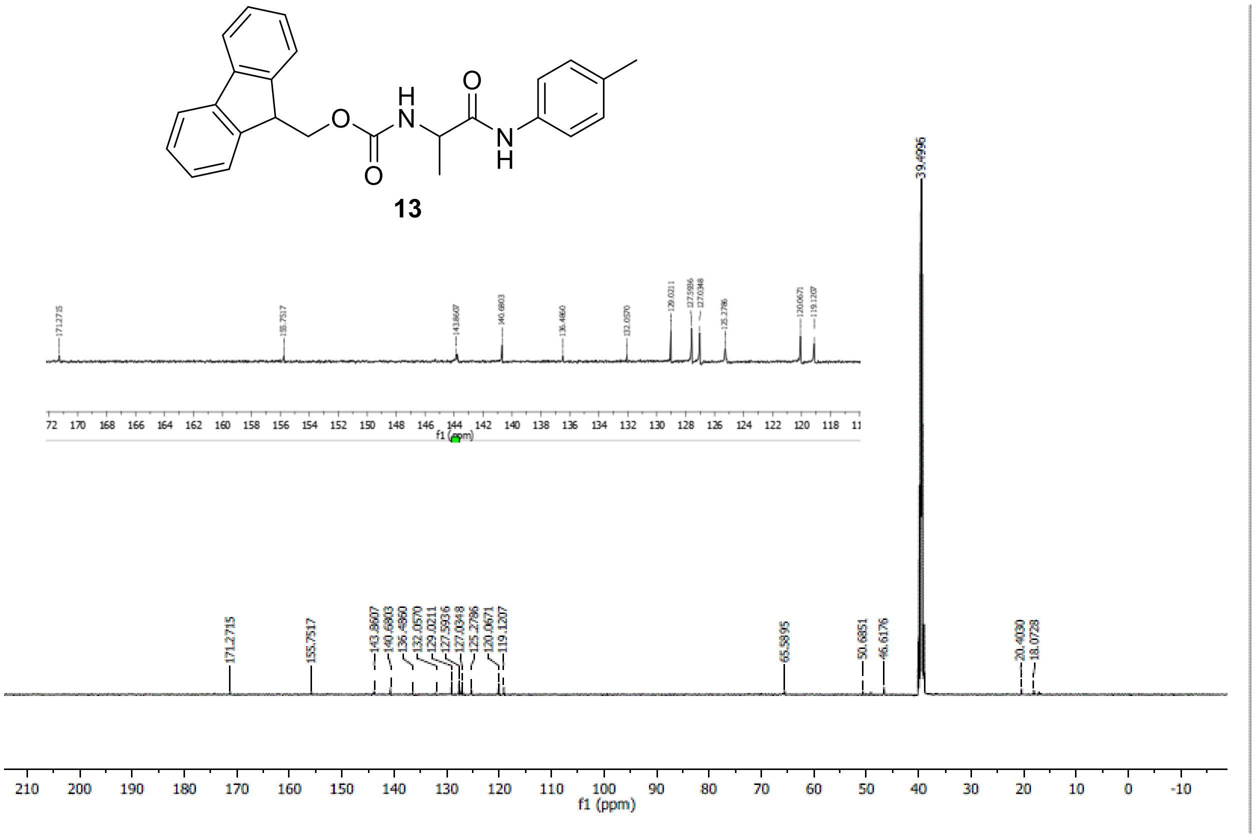
13

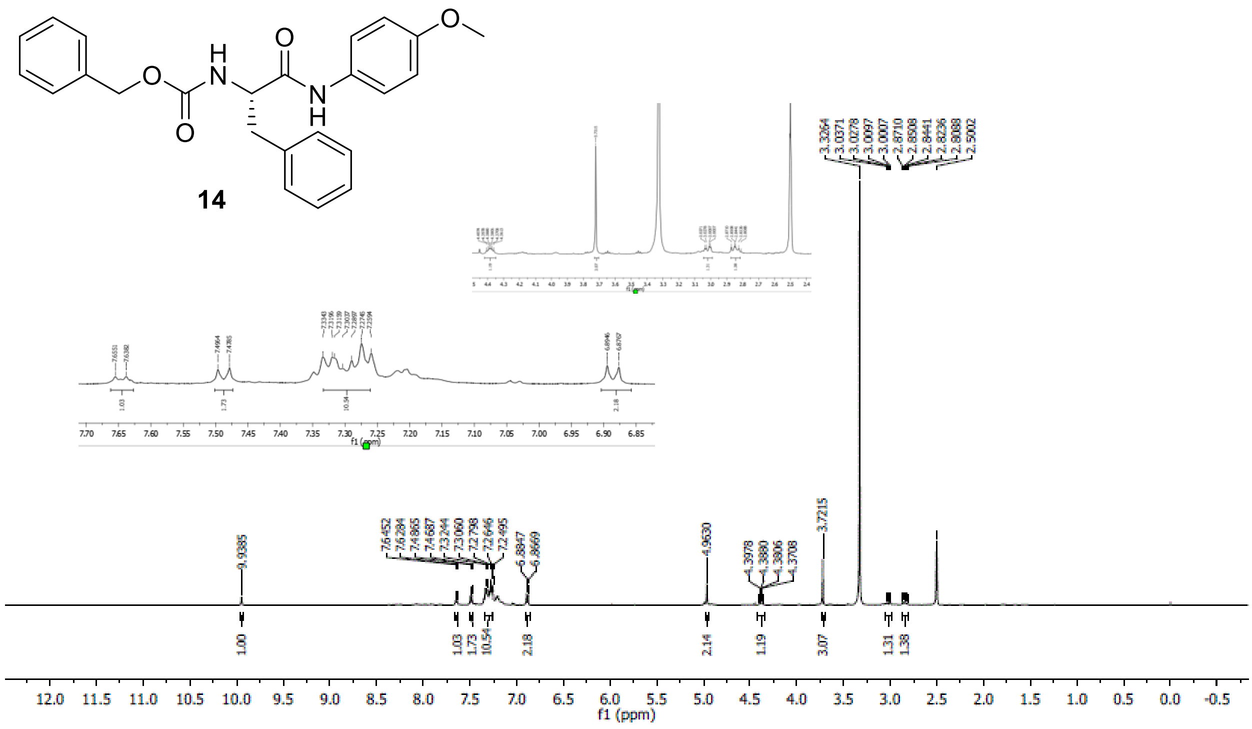
14
9.9385
4.9630
3.7215
1.00
1.03
1.73
10.54
2.18
2.14
1.19
3.07
1.31
1.38
f1 (ppm)

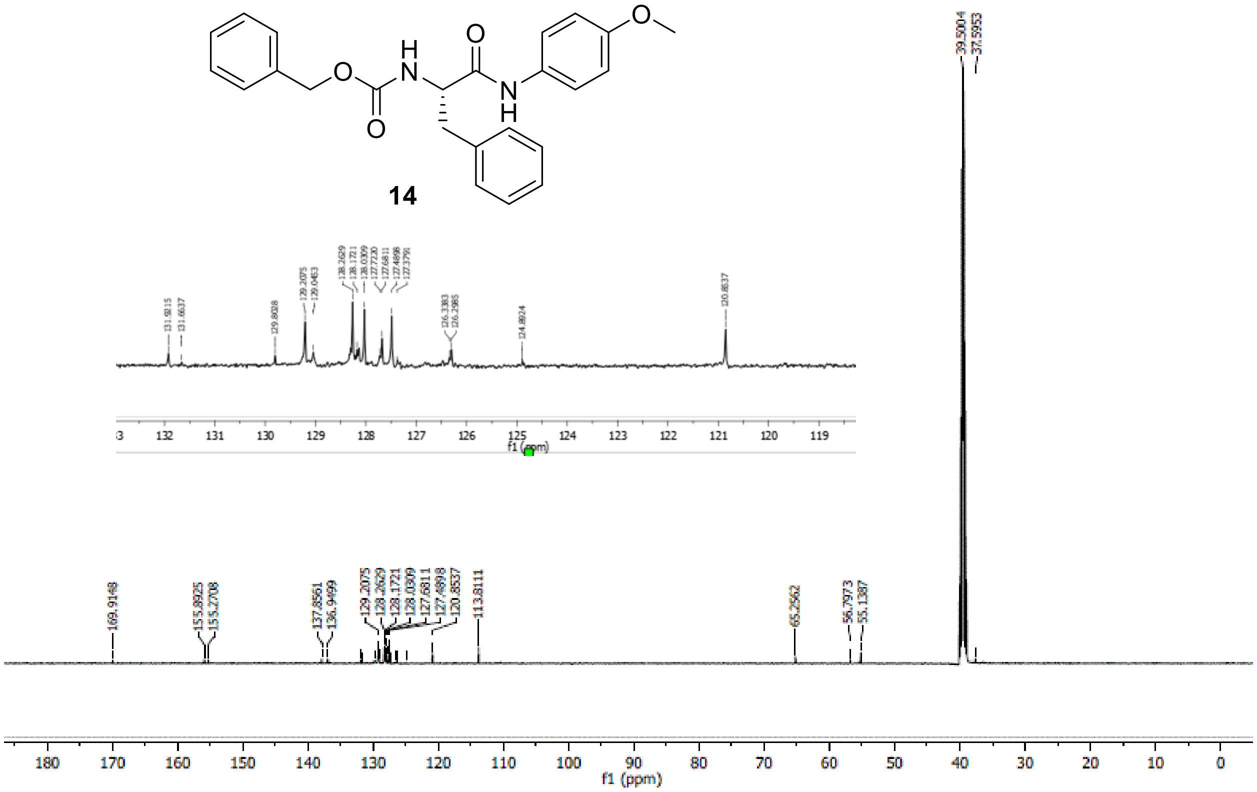
14
169.9148
155.8925
155.2708
137.8561
136.9499
113.8111
65.2562
56.7973
55.1387
39.5004
37.5953
f1 (ppm)

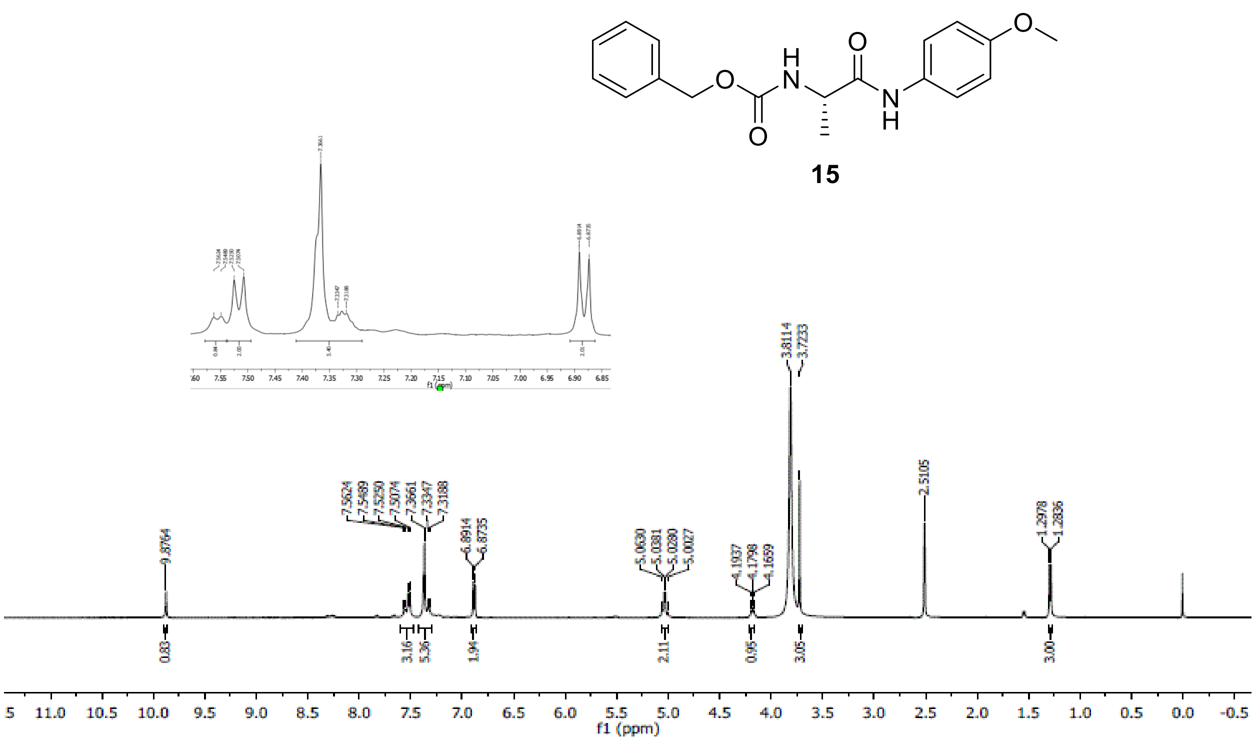

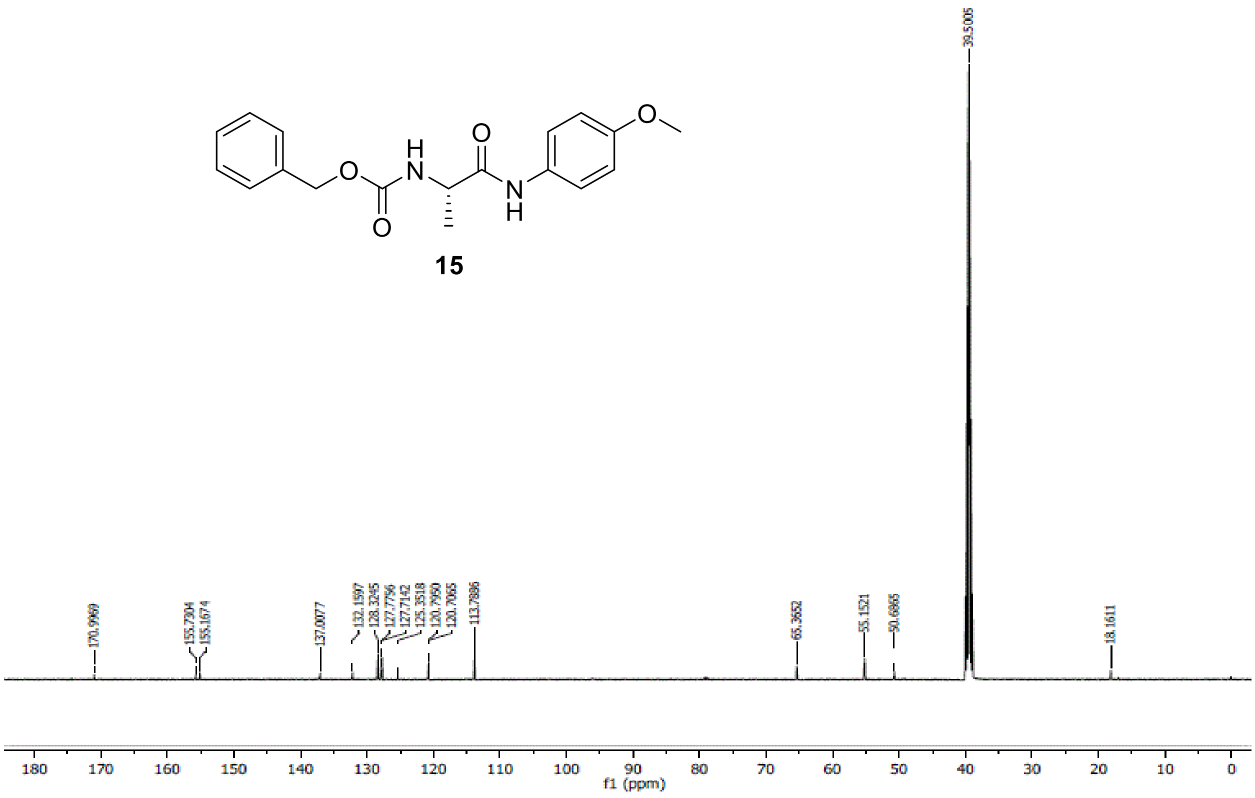

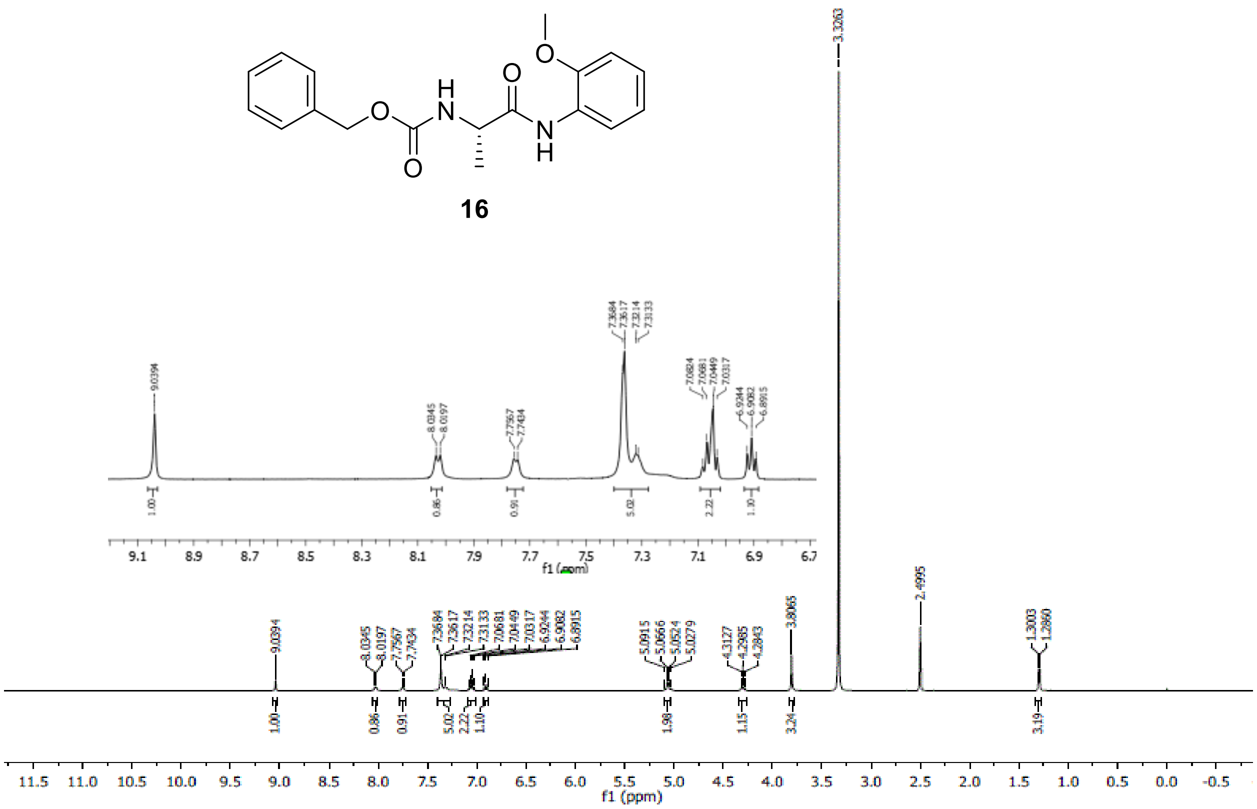

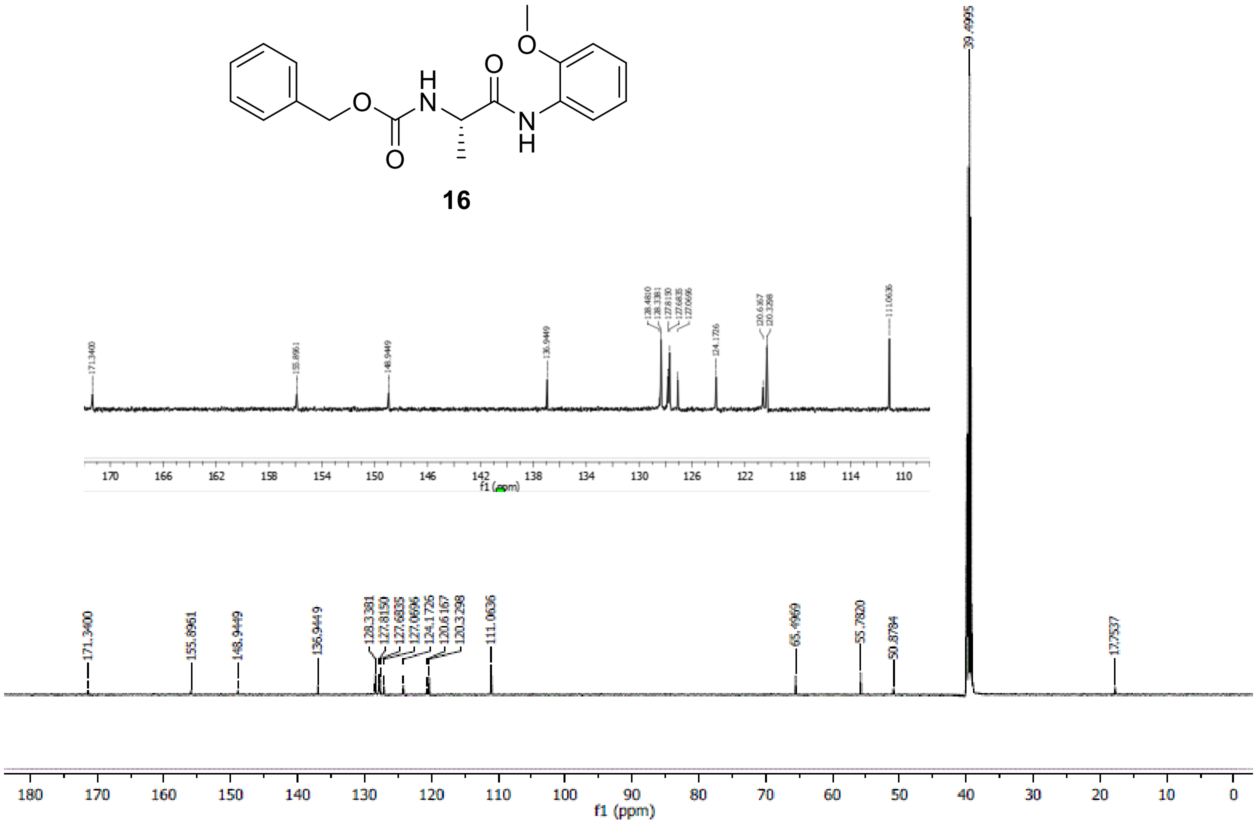

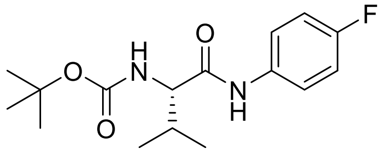

17

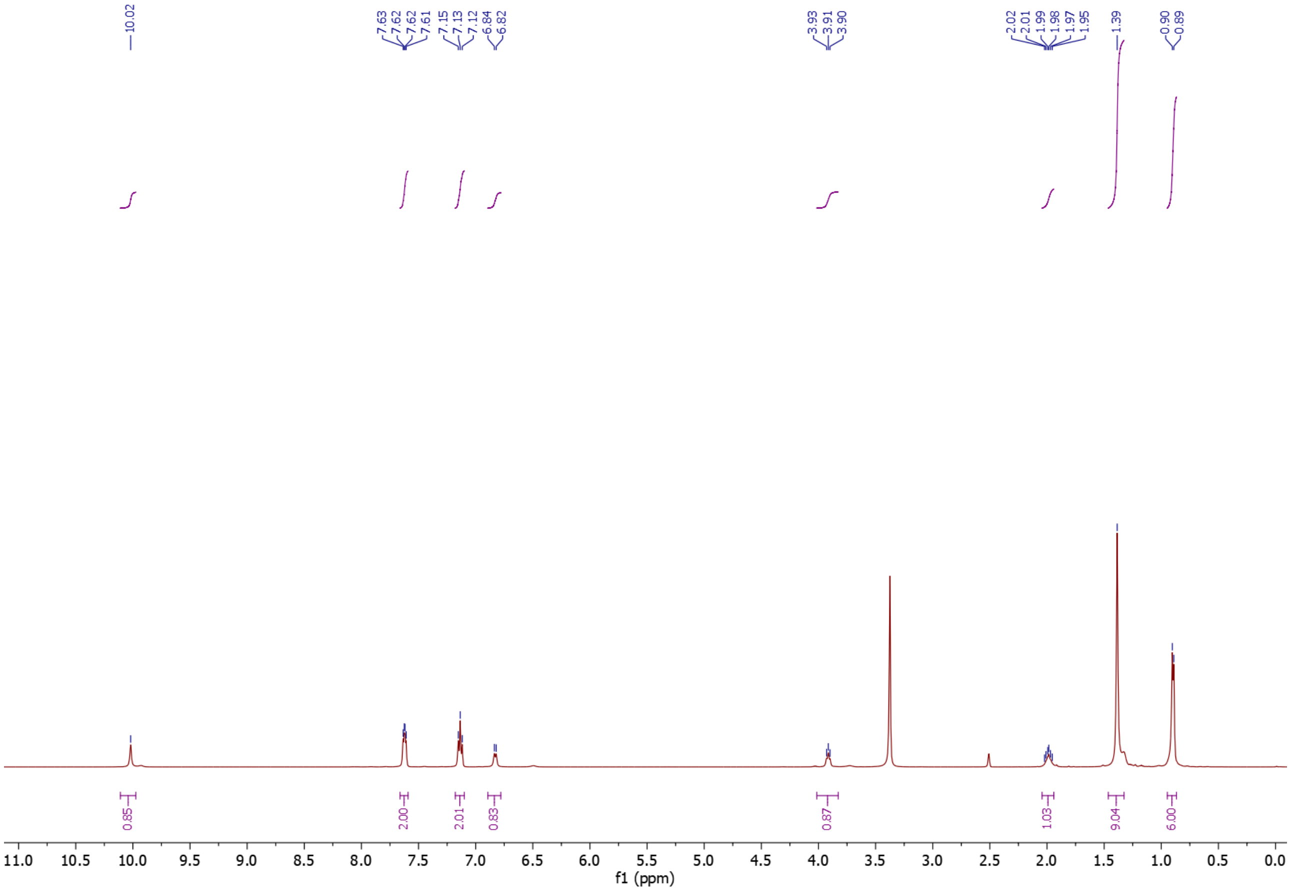
10.02
7.63
7.62
7.62
7.61
7.15
7.13
7.12
6.84
6.82
3.93
3.91
3.90
2.02
2.01
1.99
1.98
1.97
1.95
1.39
0.90
0.89
0.85
2.00
2.01
0.83
0.87
1.03
9.04
6.00
11.0 10.5 10.0 9.5 9.0 8.5 8.0 7.5 7.0 6.5 6.0 5.5 5.0 4.5 4.0 3.5 3.0 2.5 2.0 1.5 1.0 0.5 0.0
f1 (ppm)

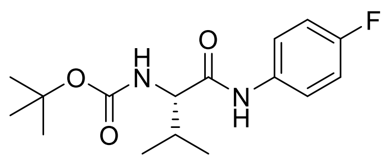
O
H
N
O
N
H
F
O

17

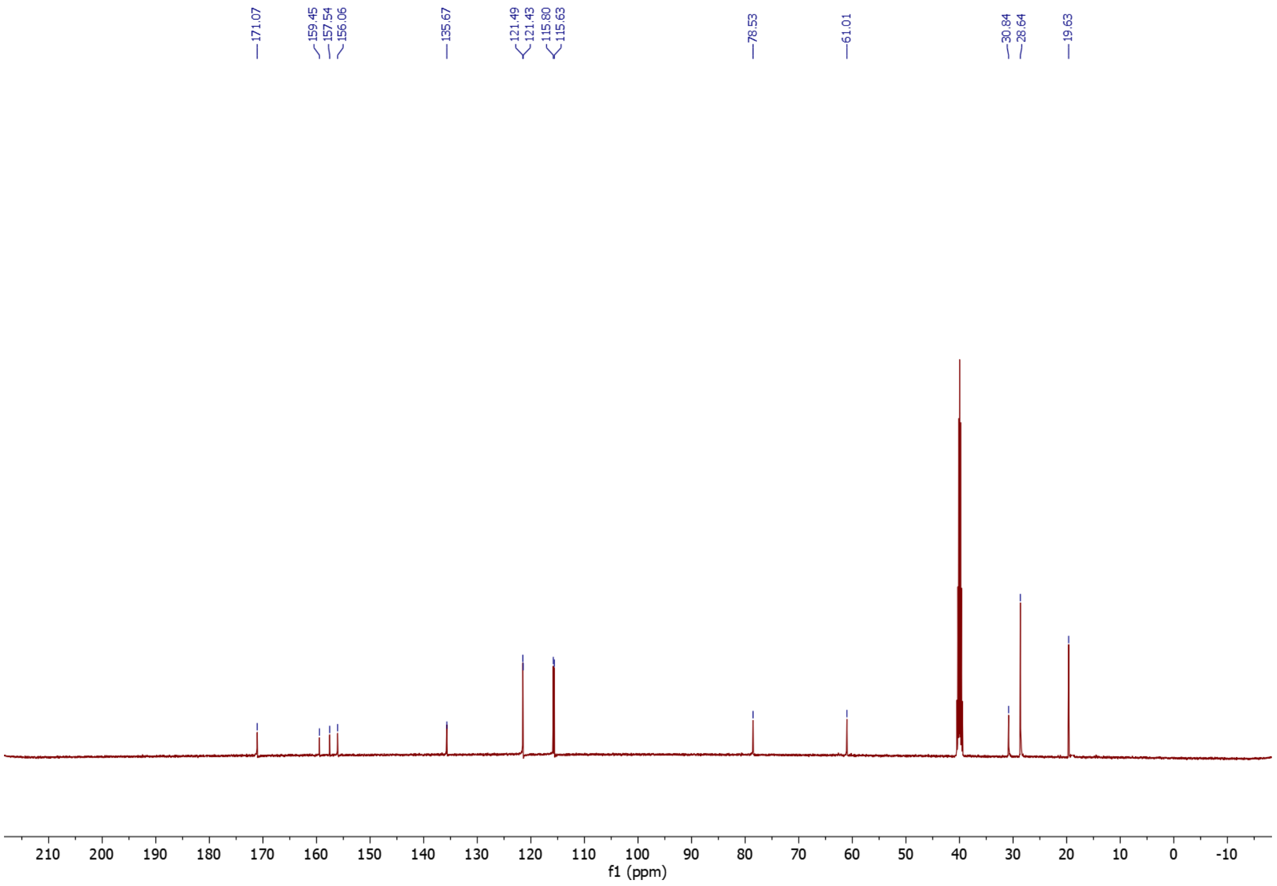
171.07
159.45
157.54
156.06
135.67
121.49
121.43
115.80
115.63
78.53
61.01
30.84
28.64
19.63
210
200
190
180
170
160
150
140
130
120
110
100
90
80
70
60
50
40
30
20
10
0
-10
f1 (ppm)

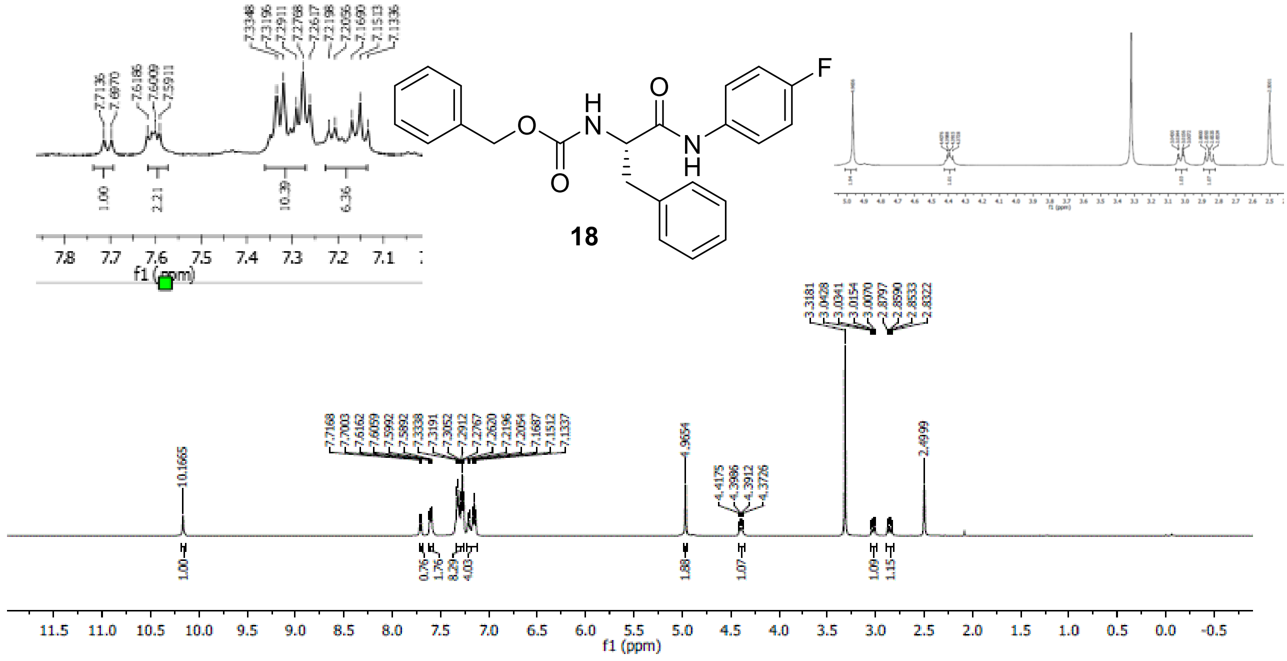

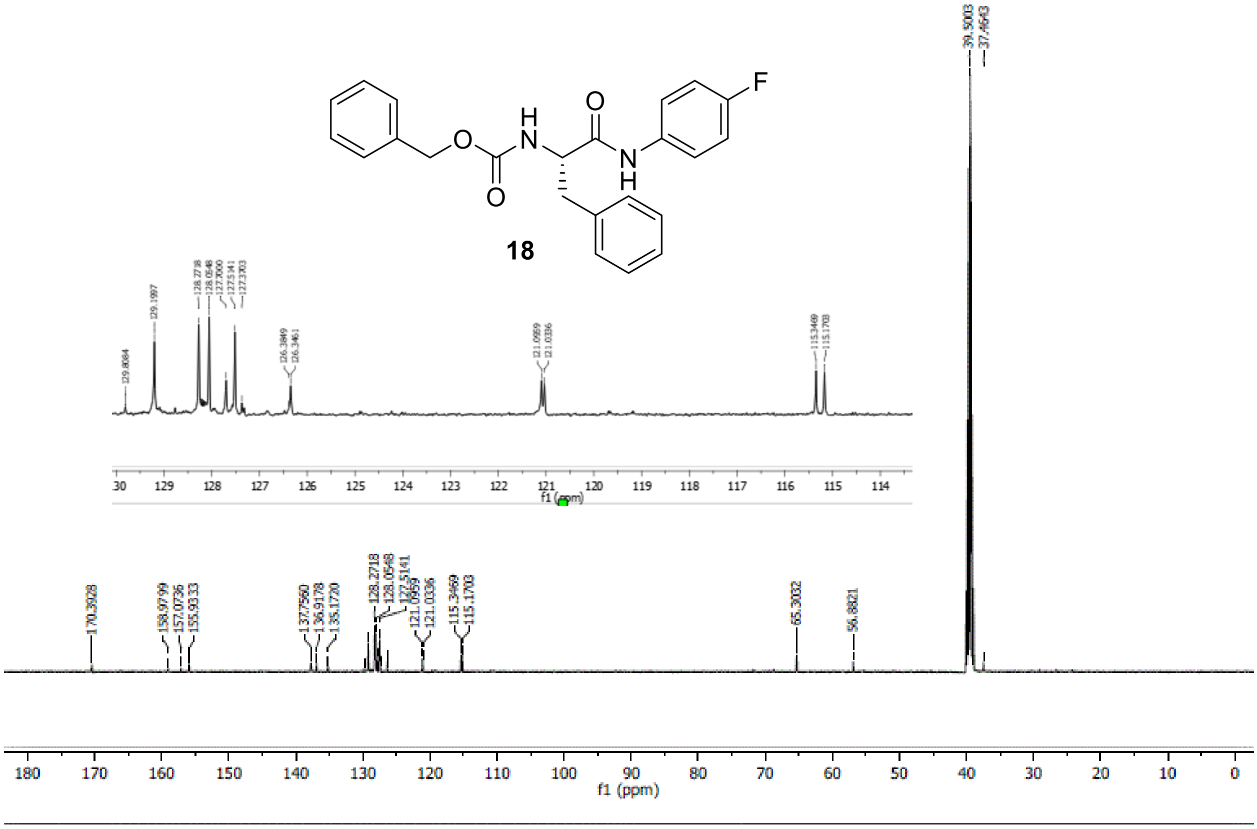

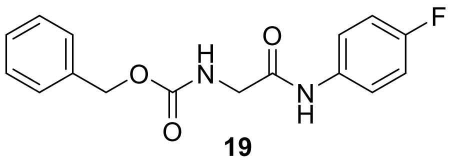

19

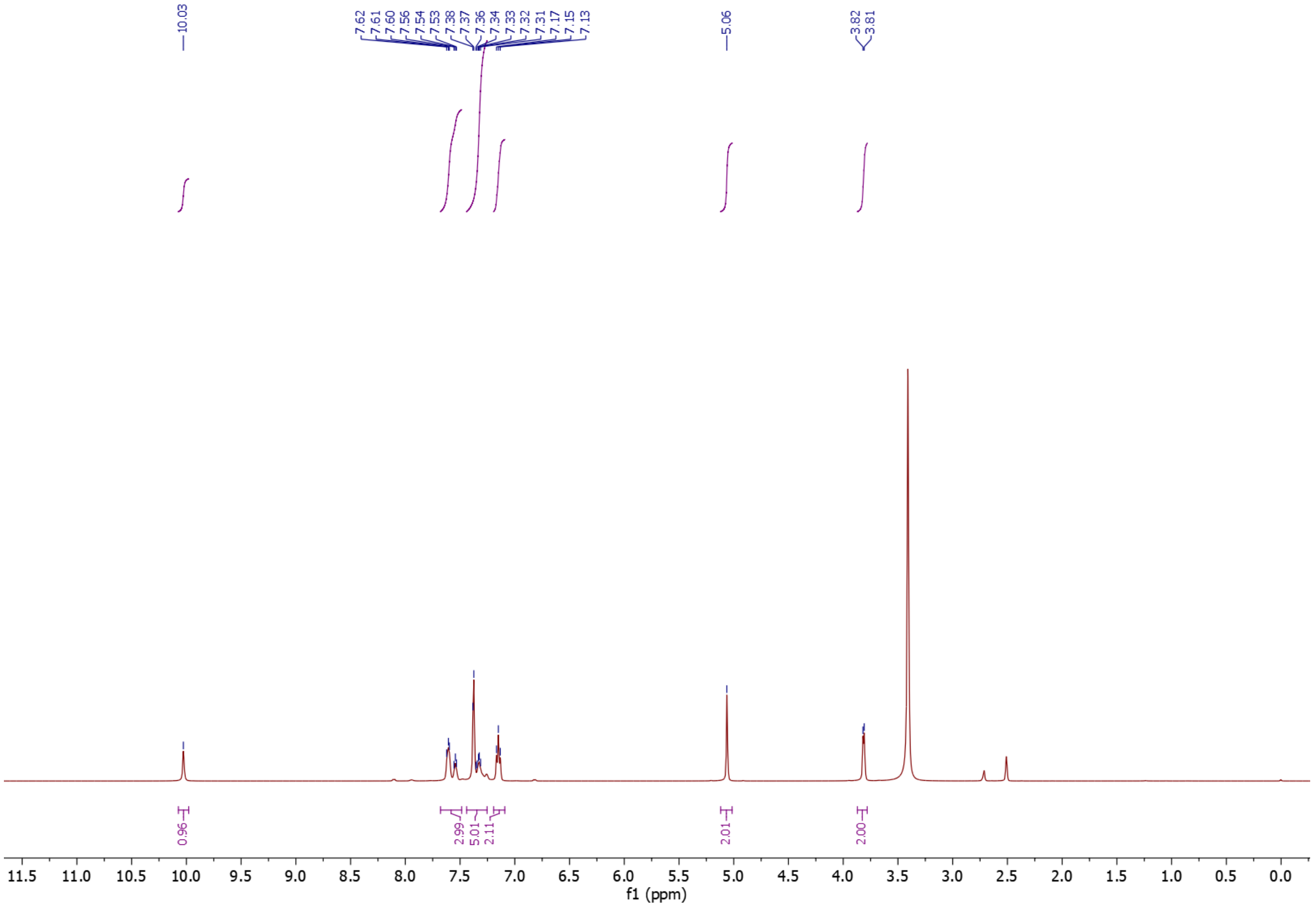
10.03
7.62
7.61
7.60
7.56
7.54
7.53
7.38
7.37
7.36
7.34
7.33
7.32
7.31
7.17
7.15
7.13
5.06
3.82
3.81
0.96
2.99
5.01
2.11
2.01
2.00
11.5 11.0 10.5 10.0 9.5 9.0 8.5 8.0 7.5 7.0 6.5 6.0 5.5 5.0 4.5 4.0 3.5 3.0 2.5 2.0 1.5 1.0 0.5 0.0
f1 (ppm)

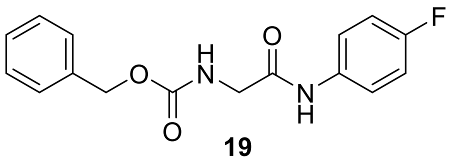

19

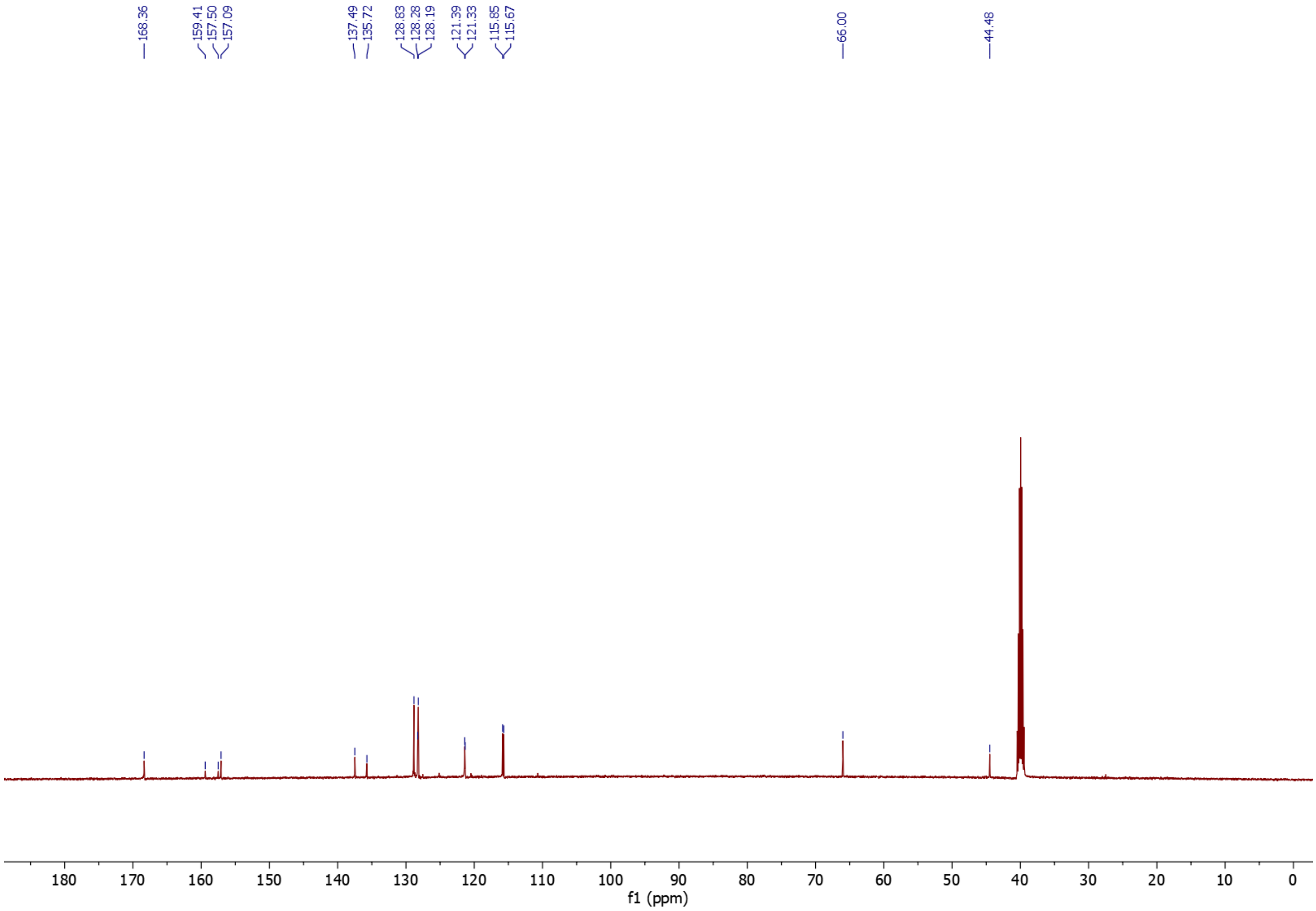
168.36
159.41
157.50
157.09
137.49
135.72
128.83
128.28
128.19
121.39
121.33
115.85
115.67
66.00
44.48
180
170
160
150
140
130
120
110
100
90
80
70
60
50
40
30
20
10
0
f1 (ppm)

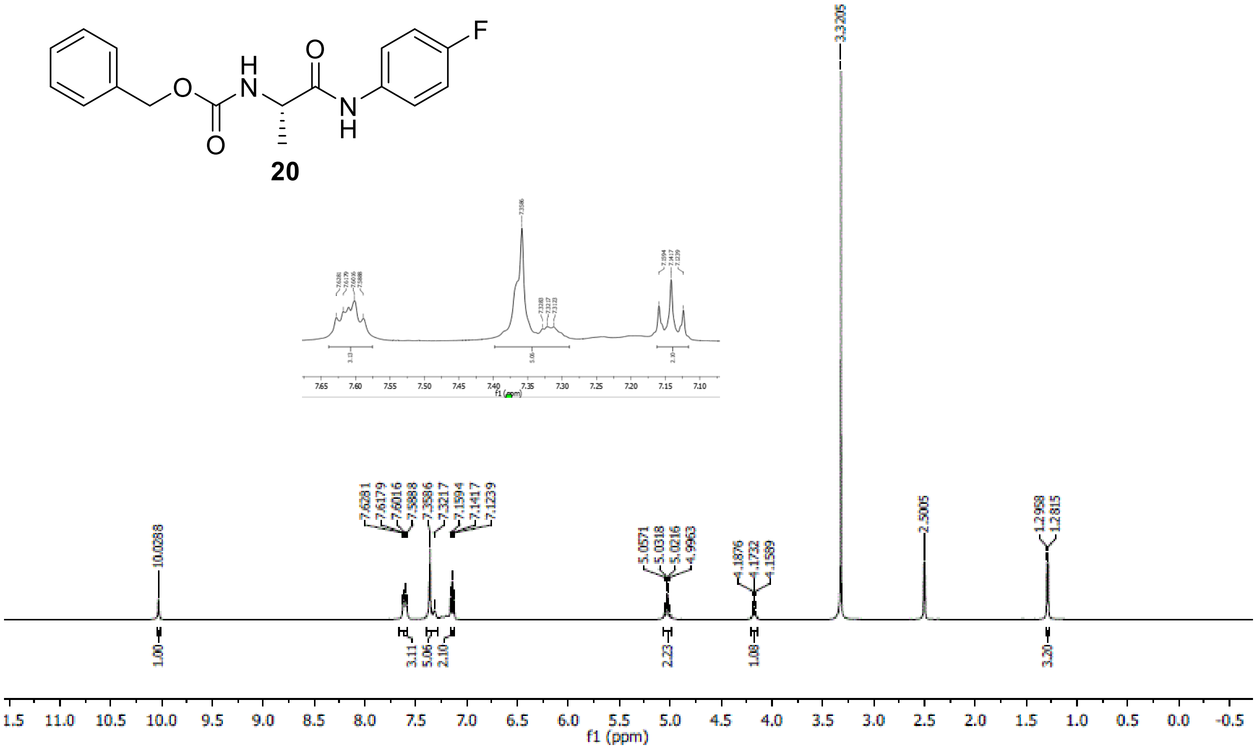
F
O
H
N
O
N
H
O
20
3.3205
10.0288
7.6281
7.6179
7.6016
7.5888
7.3586
7.3217
7.1594
7.1417
7.1239
5.0571
5.0318
5.0216
4.9963
4.1876
4.1732
4.1589
2.5005
1.2958
1.2815
1.00
3.11
5.06
2.10
2.23
1.08
3.20
f1 (ppm)

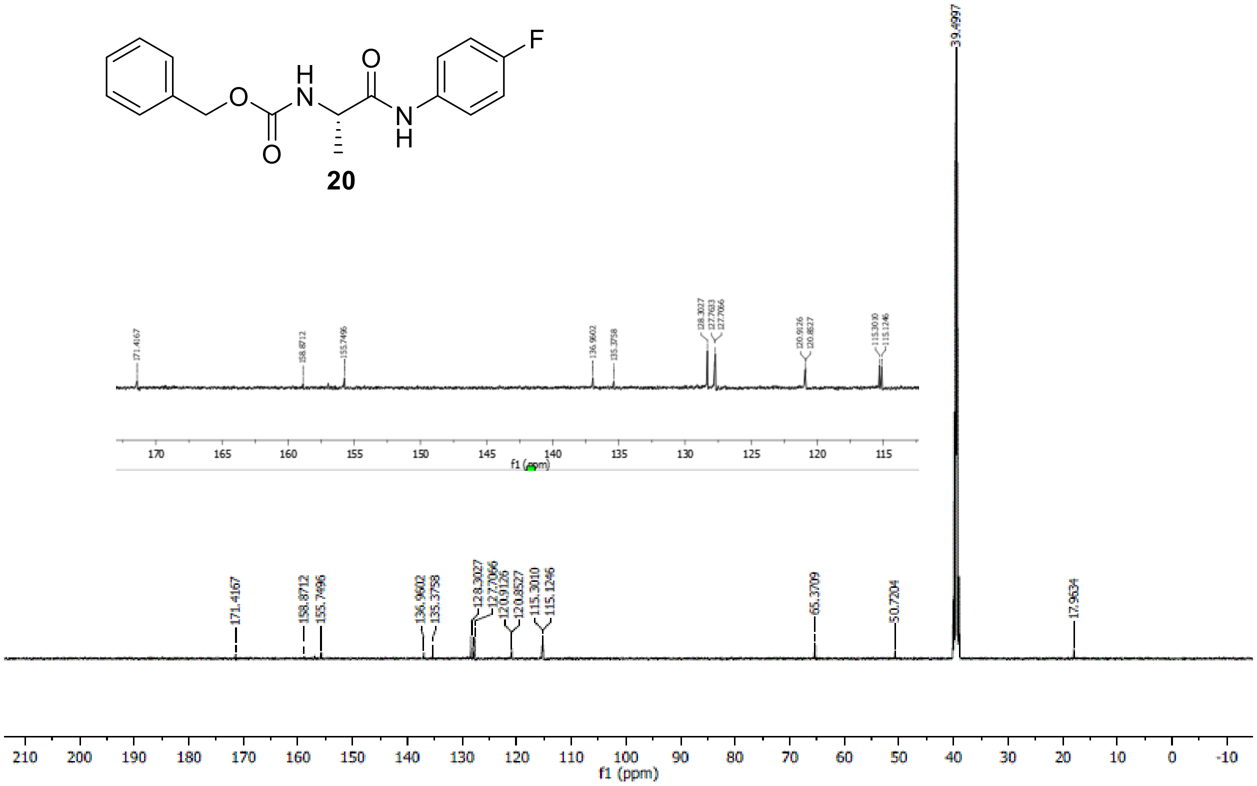
F
O
H
N
O
N
H
O
20
39.4997
171.4167
158.8712
155.7496
136.9602
135.3758
128.3027
127.7633
127.7066
120.9126
120.8527
115.3010
115.1246
65.3709
50.7204
17.9634
f1 (ppm)

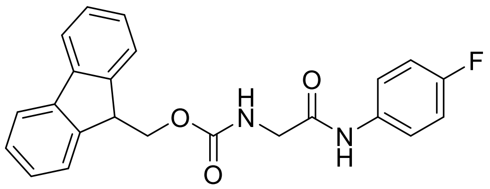

21

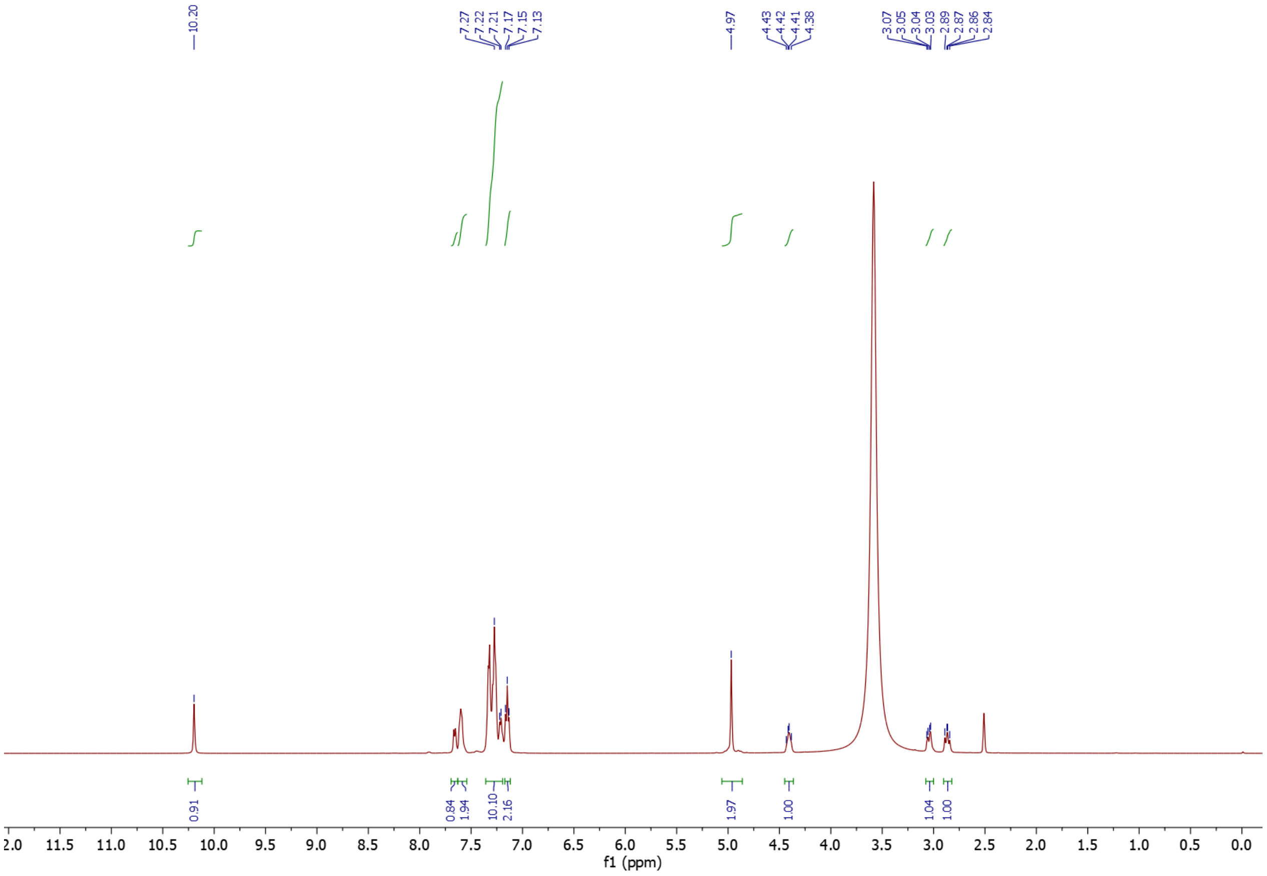

10.20
7.27
7.22
7.21
7.17
7.15
7.13
4.97
4.43
4.42
4.41
4.38
3.07
3.05
3.04
3.03
2.89
2.87
2.86
2.84
0.91
0.84
1.94
10.10
2.16
1.97
1.00
1.04
1.00
2.0
11.5
11.0
10.5
10.0
9.5
9.0
8.5
8.0
7.5
7.0
6.5
6.0
5.5
5.0
4.5
4.0
3.5
3.0
2.5
2.0
1.5
1.0
0.5
0.0
f1 (ppm)

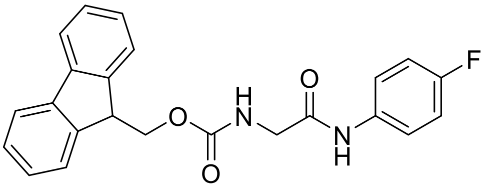
O
H
N
O
N
H
F
O

21

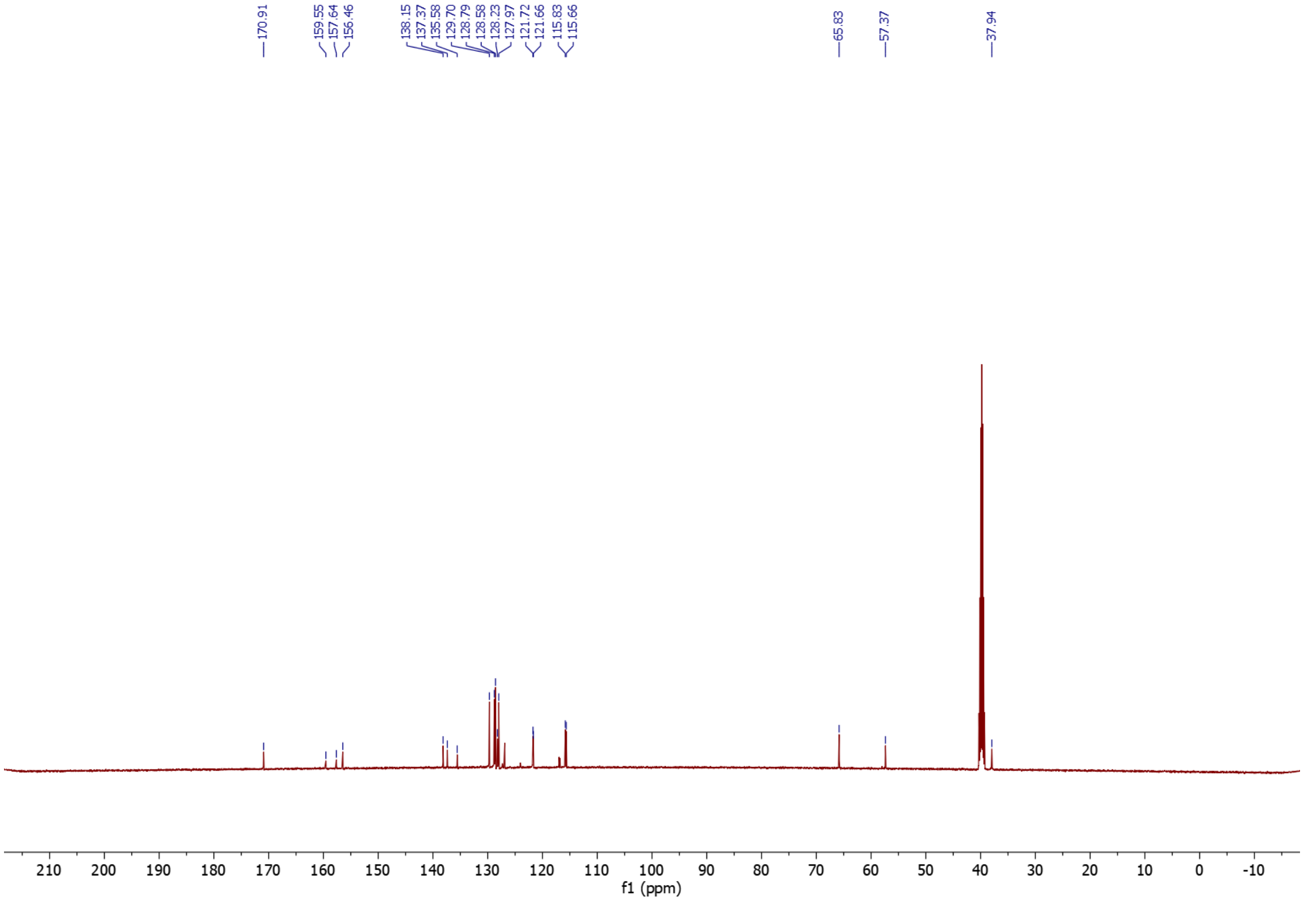
170.91
159.55
157.64
156.46
138.15
137.37
135.58
129.70
128.79
128.58
128.23
127.97
121.72
121.66
115.83
115.66
65.83
57.37
37.94
210 200 190 180 170 160 150 140 130 120 110 100 90 80 70 60 50 40 30 20 10 0 -10
f1 (ppm)

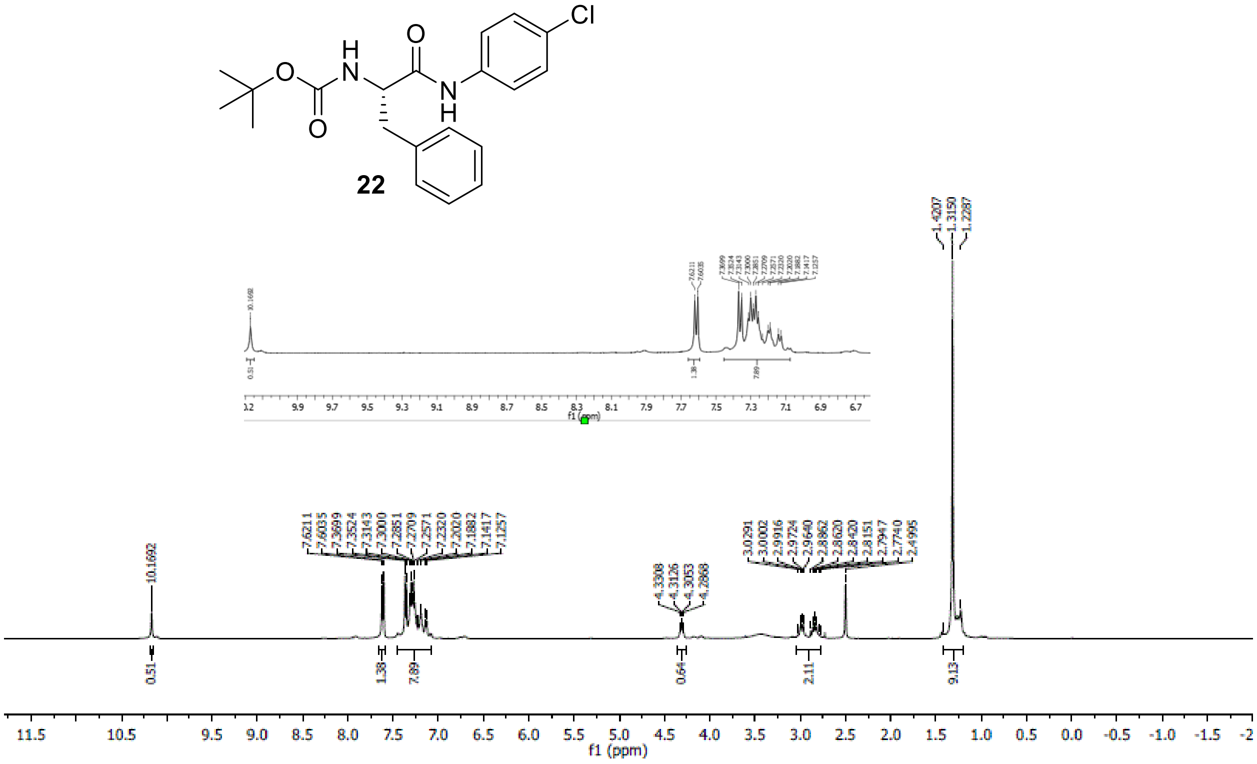
22
f1 (ppm)

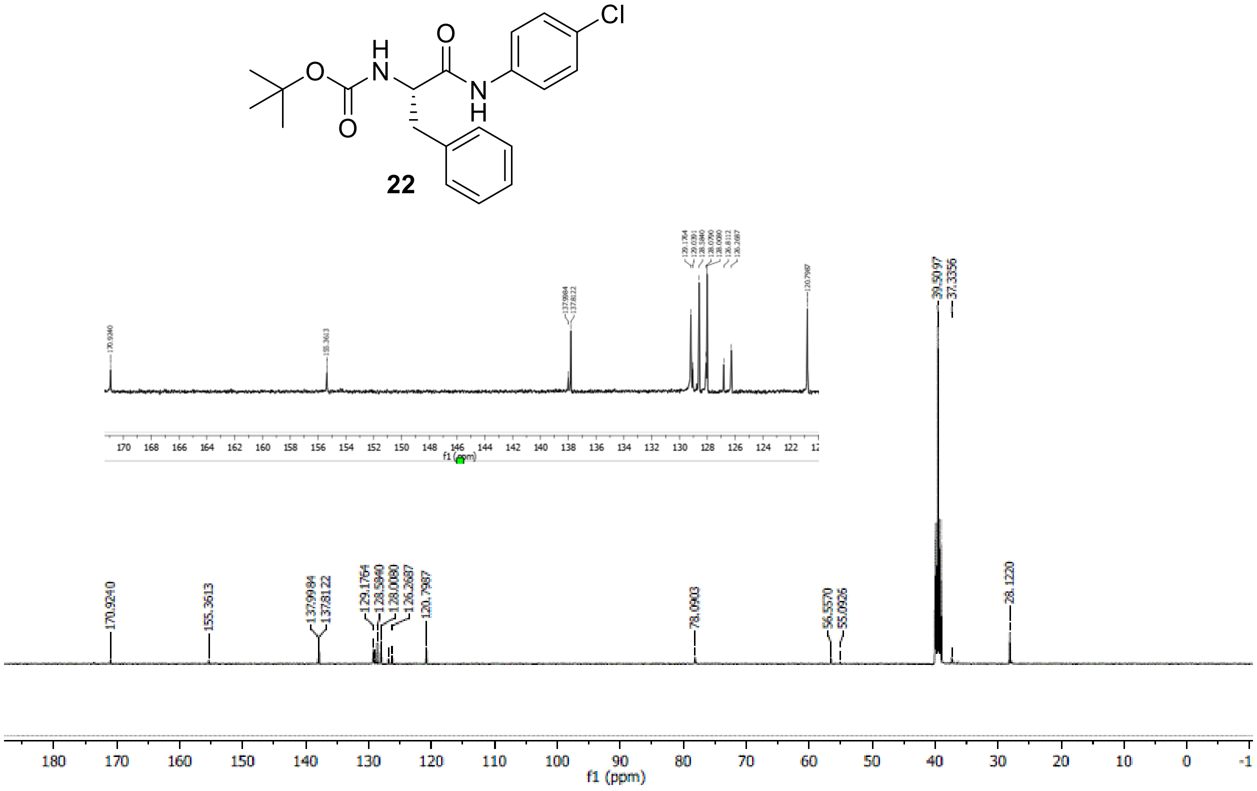
22
f1 (ppm)

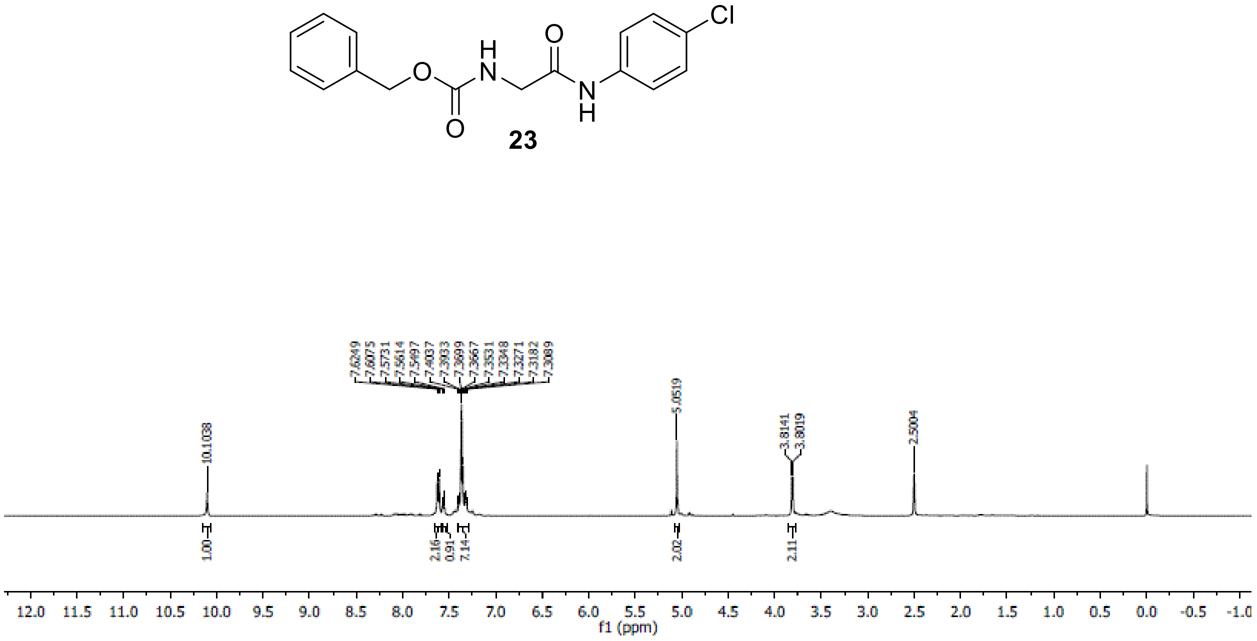
Cl
O
H
N
N
H
O
O
23
10.1038
5.0519
3.8141
3.8019
2.5004
1.00
2.16
0.91
7.14
2.02
2.11
f1 (ppm)

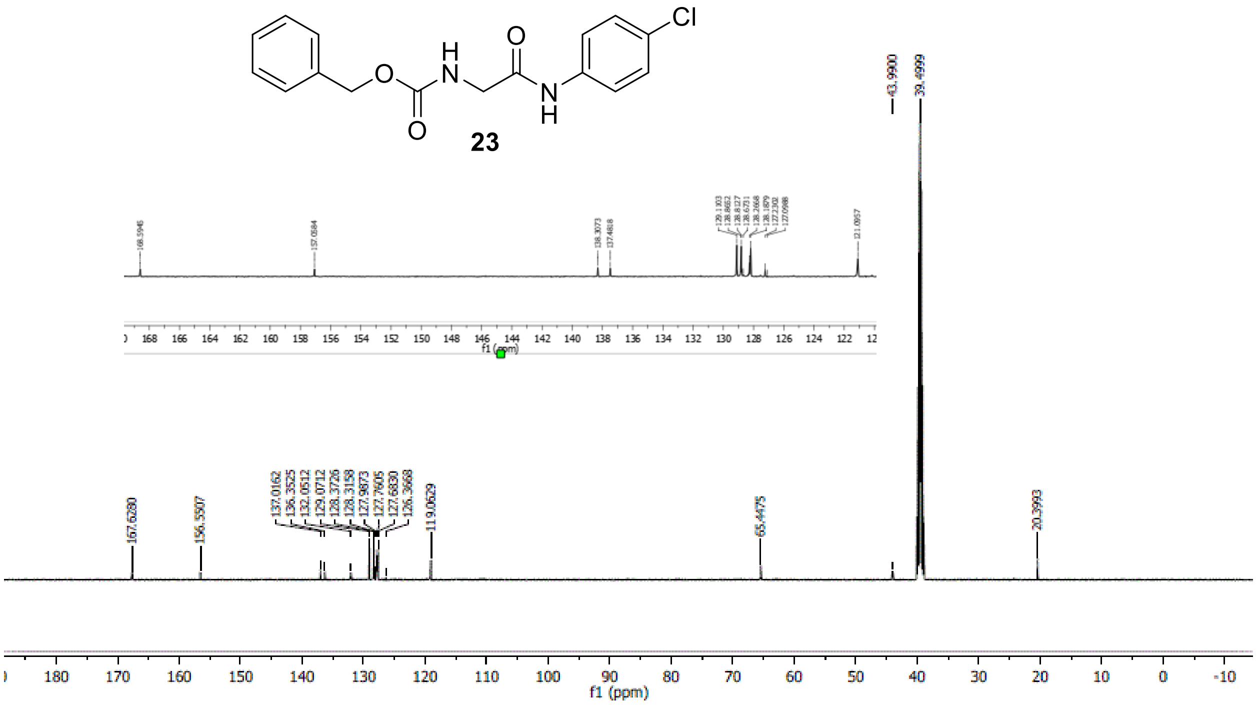
Cl
23
43.9900
39.4999
167.6280
156.5507
119.0629
65.4475
20.3993
f1 (ppm)

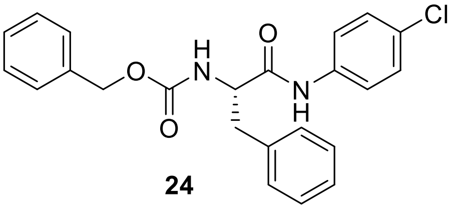
Cl
O
H
N
O
N
H
O

24

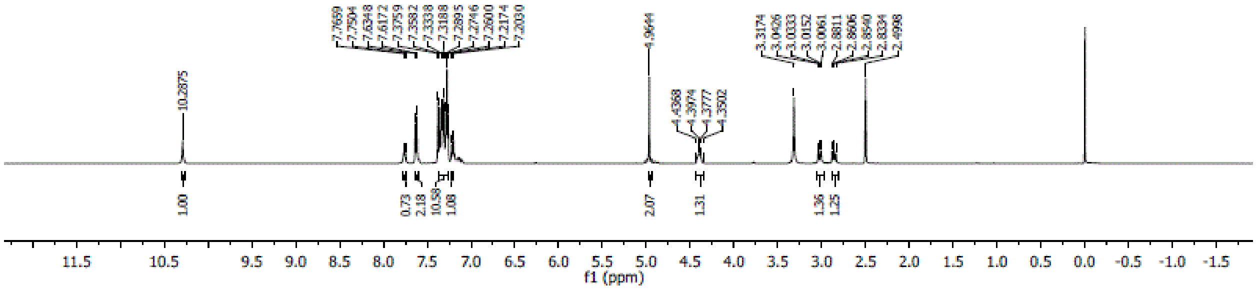
10.2875
7.7669
7.7504
7.6348
7.6172
7.3759
7.3582
7.3338
7.3188
7.2895
7.2746
7.2600
7.2174
7.2030
4.9644
4.4368
4.3974
4.3777
4.3502
3.3174
3.0426
3.0333
3.0152
3.0061
2.8811
2.8606
2.8540
2.8334
2.4998
1.00
0.73
2.18
10.58
1.08
2.07
1.31
1.36
1.25
f1 (ppm)

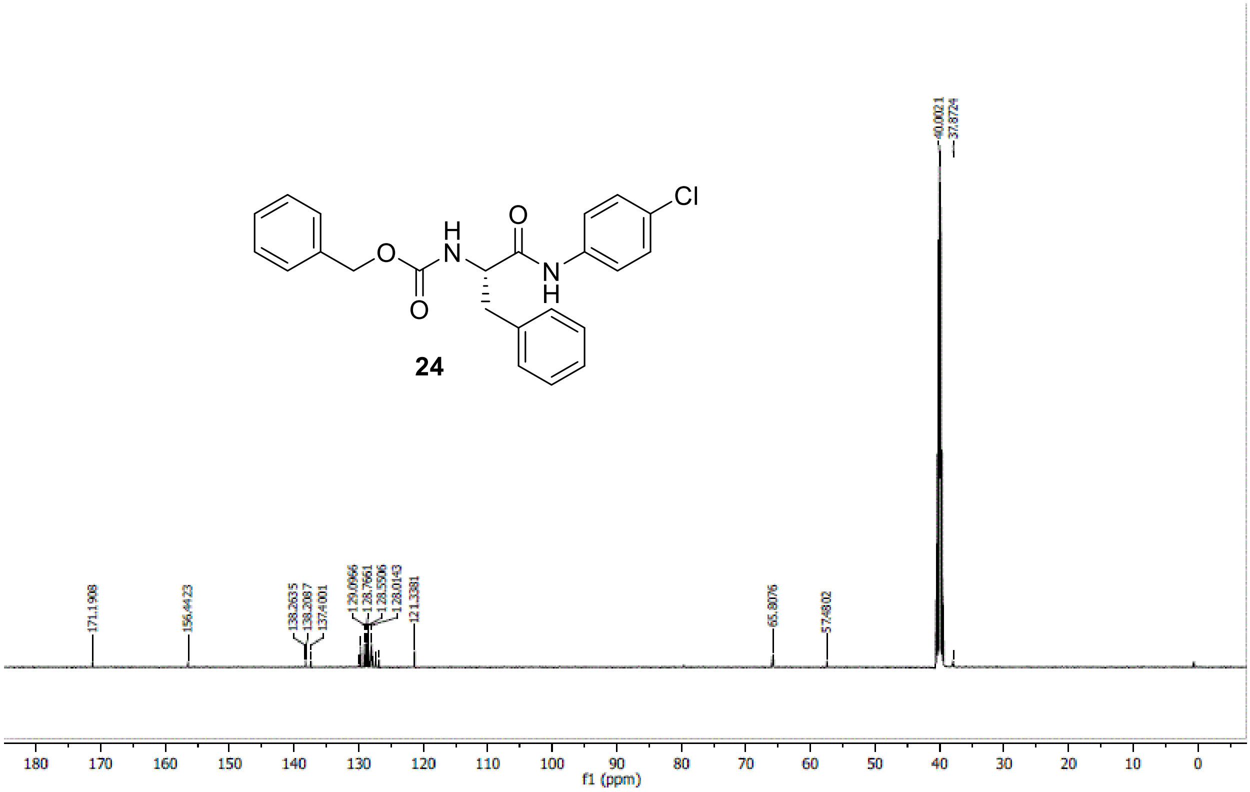
40.0021
37.8724
Cl
O
H
N
O
N
H
O
24
171.1908
156.4423
138.2635
138.2087
137.4001
129.0966
128.7661
128.5506
128.0143
121.3381
65.8076
57.4802
f1 (ppm)

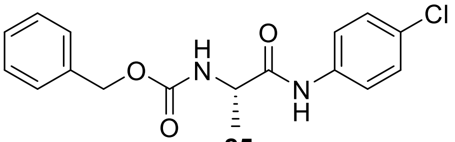

25

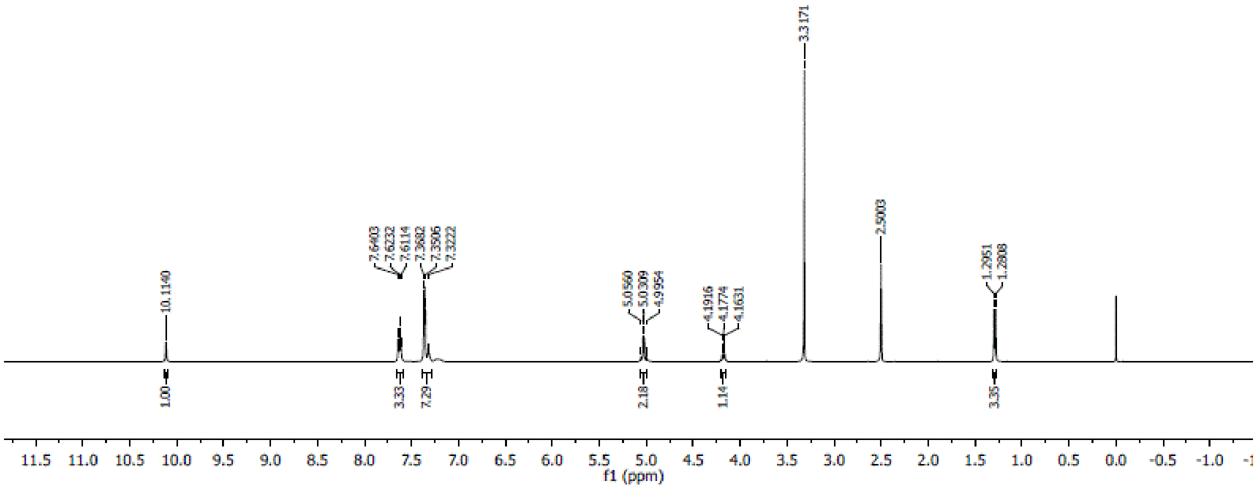

10.1140
7.6403
7.6232
7.6114
7.3682
7.3506
7.3222
5.0560
5.0309
4.9954
4.1916
4.1774
4.1631
3.3171
2.5003
1.2951
1.2808
1.00
3.33
7.29
2.18
1.14
3.35
f1 (ppm)

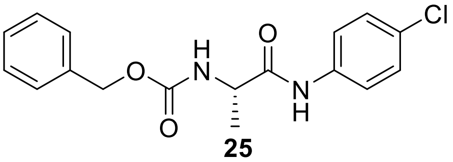

25

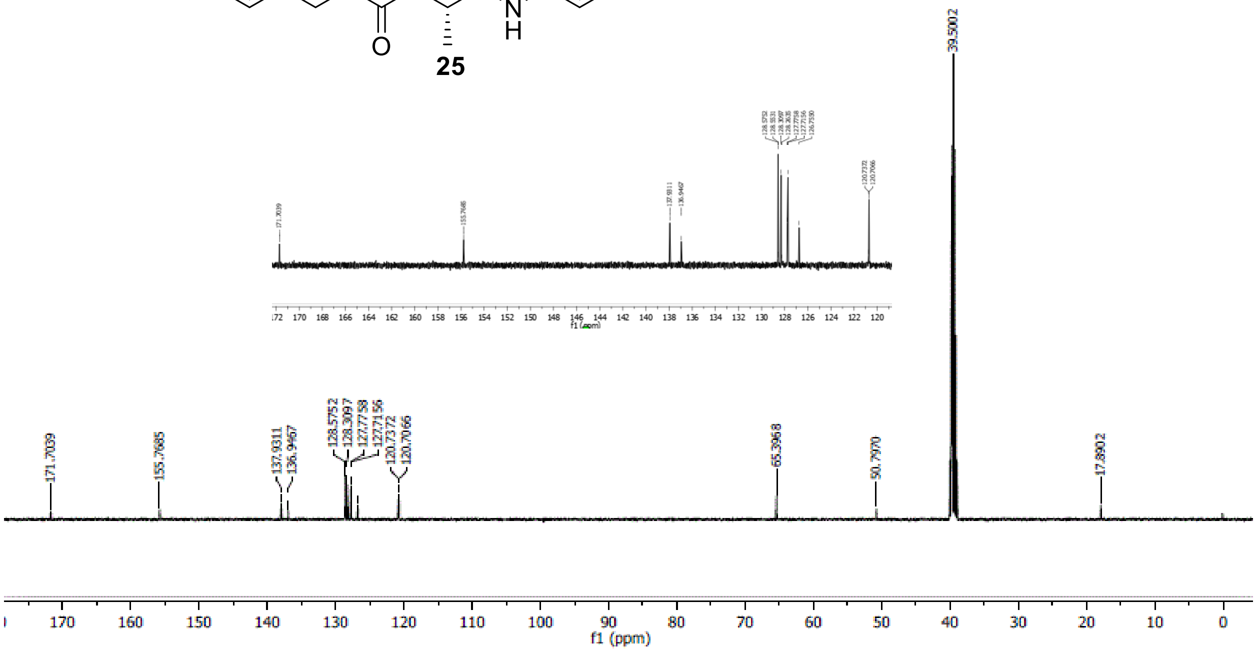

39.5002
171.7039
155.7685
137.9311
136.9467
128.5752
128.3097
127.7758
127.7156
120.7372
120.7066
65.3968
50.7970
17.8902
f1 (ppm)

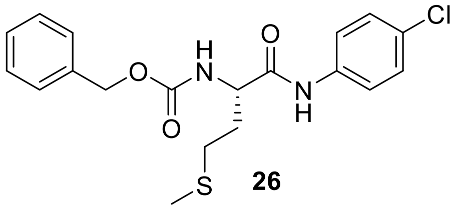

26

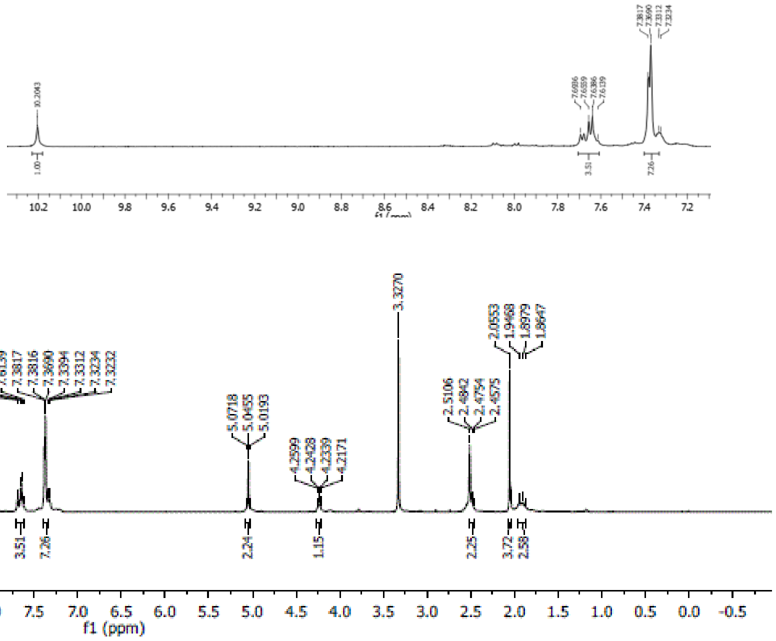

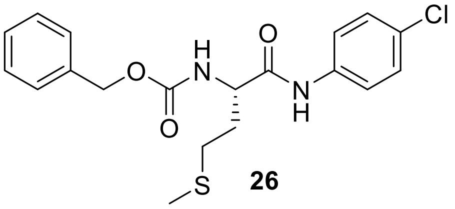

26

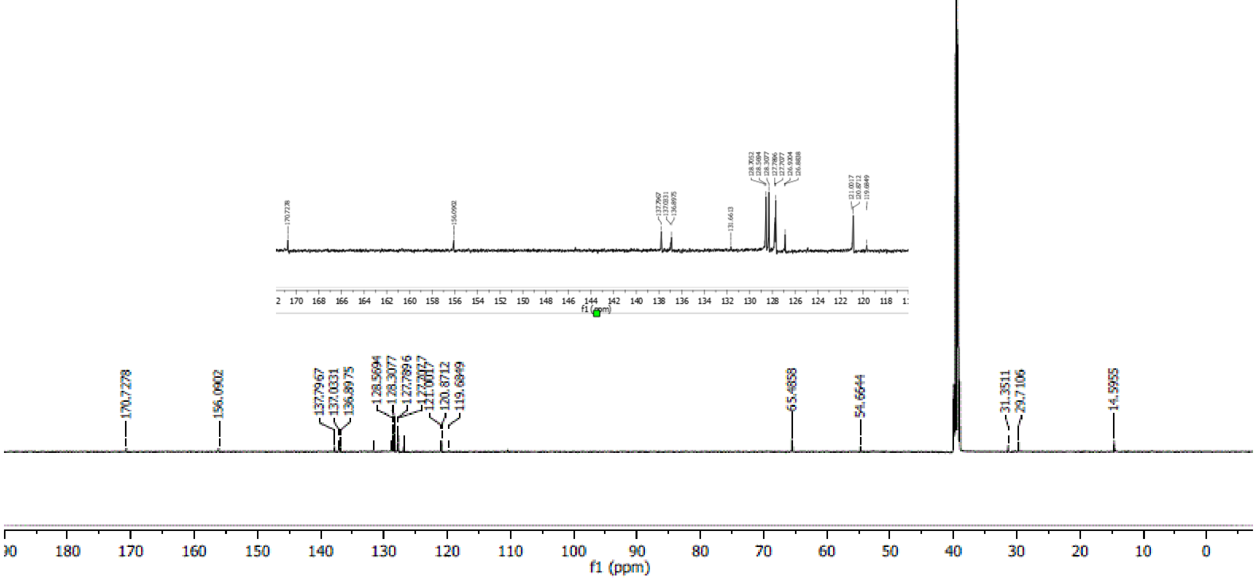

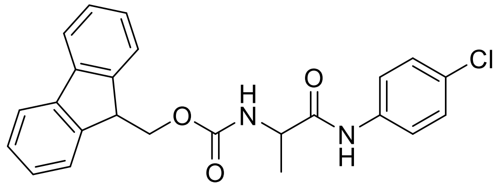
Cl
O
H
N
O
N
H
O

27

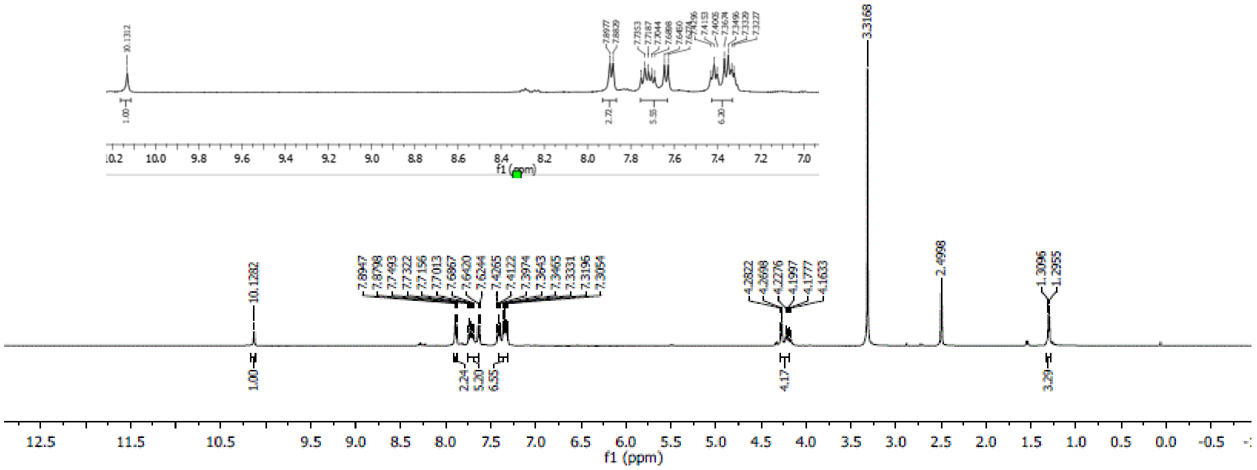
10.1282
3.3163
2.4998
1.3096
1.2955
f1 (ppm)

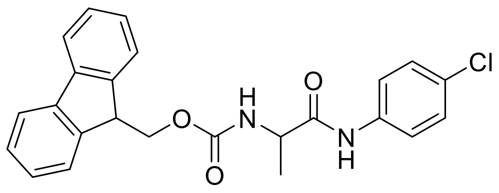
Cl
O
H
N
O
N
H
O

27

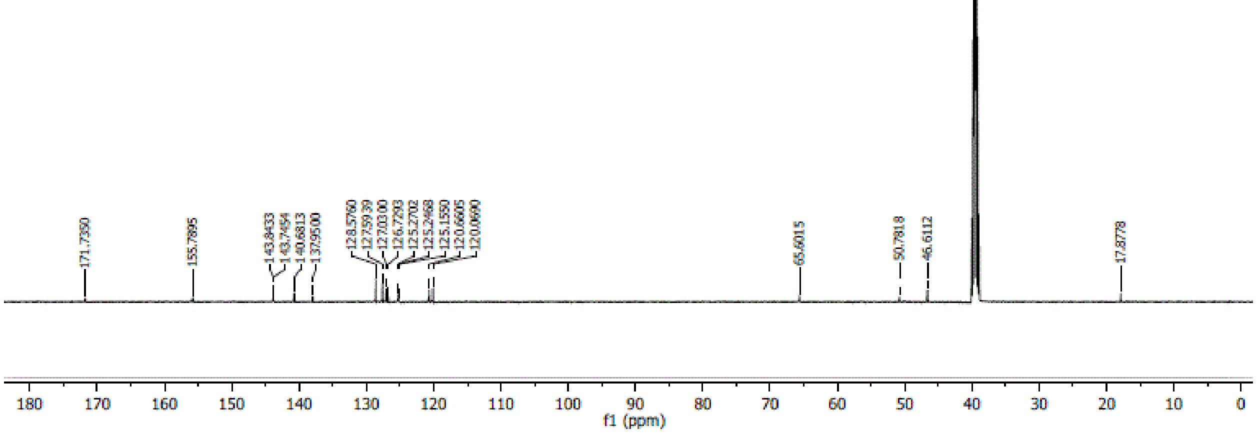
39.5004
171.7350
155.7895
65.6015
50.7818
46.6112
17.8778
f1 (ppm)

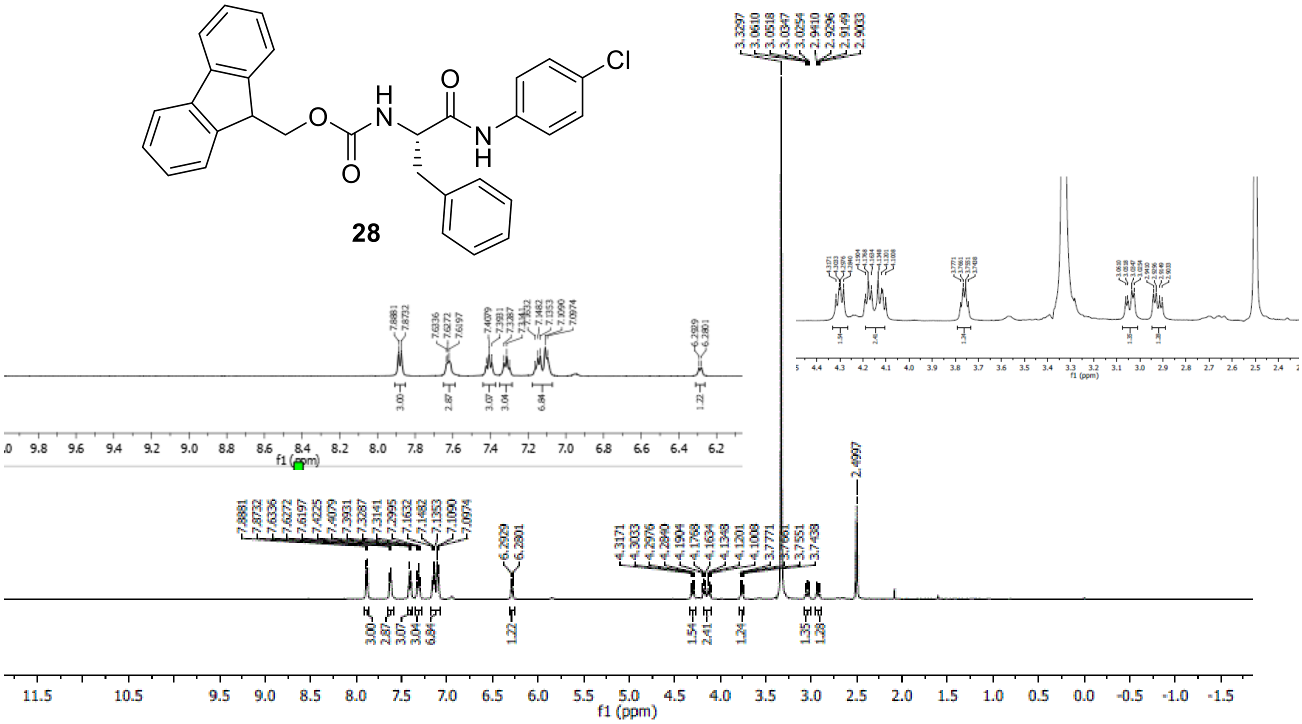

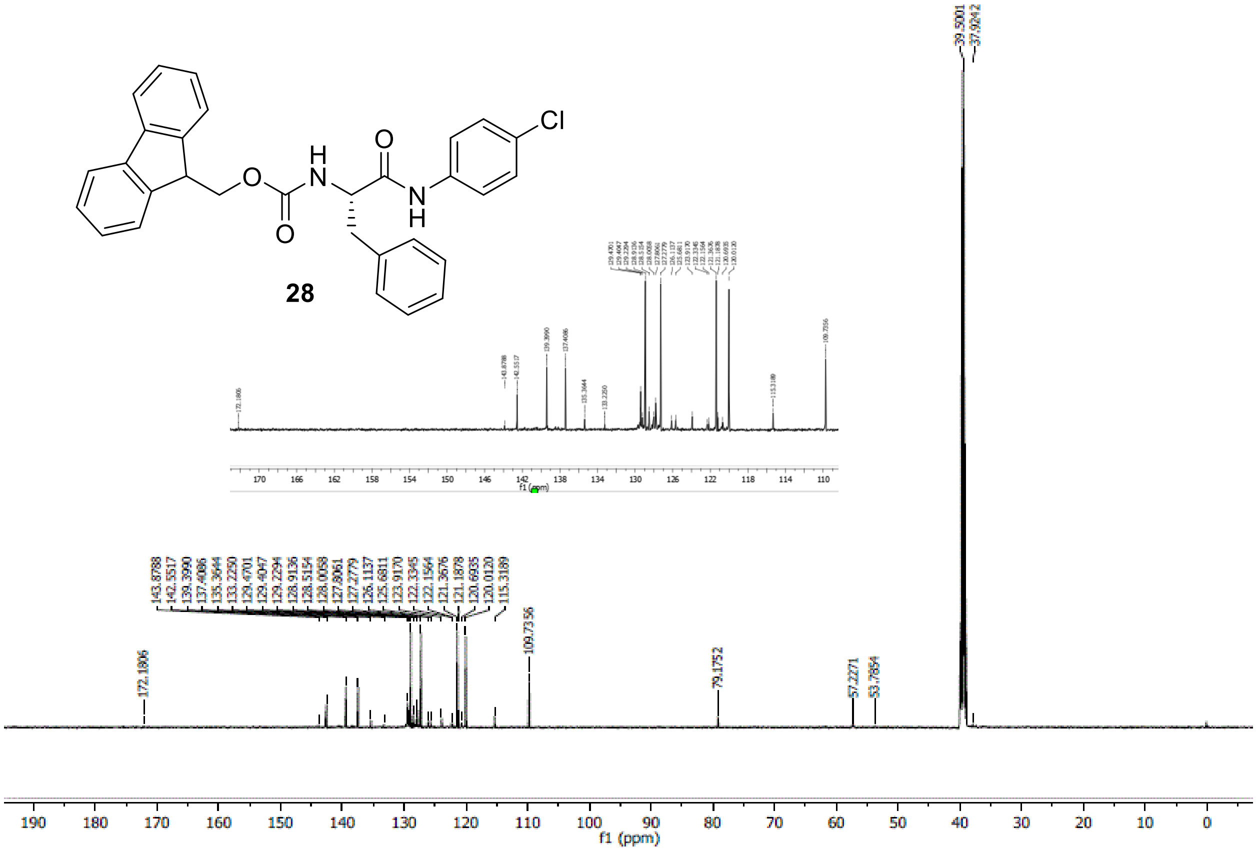

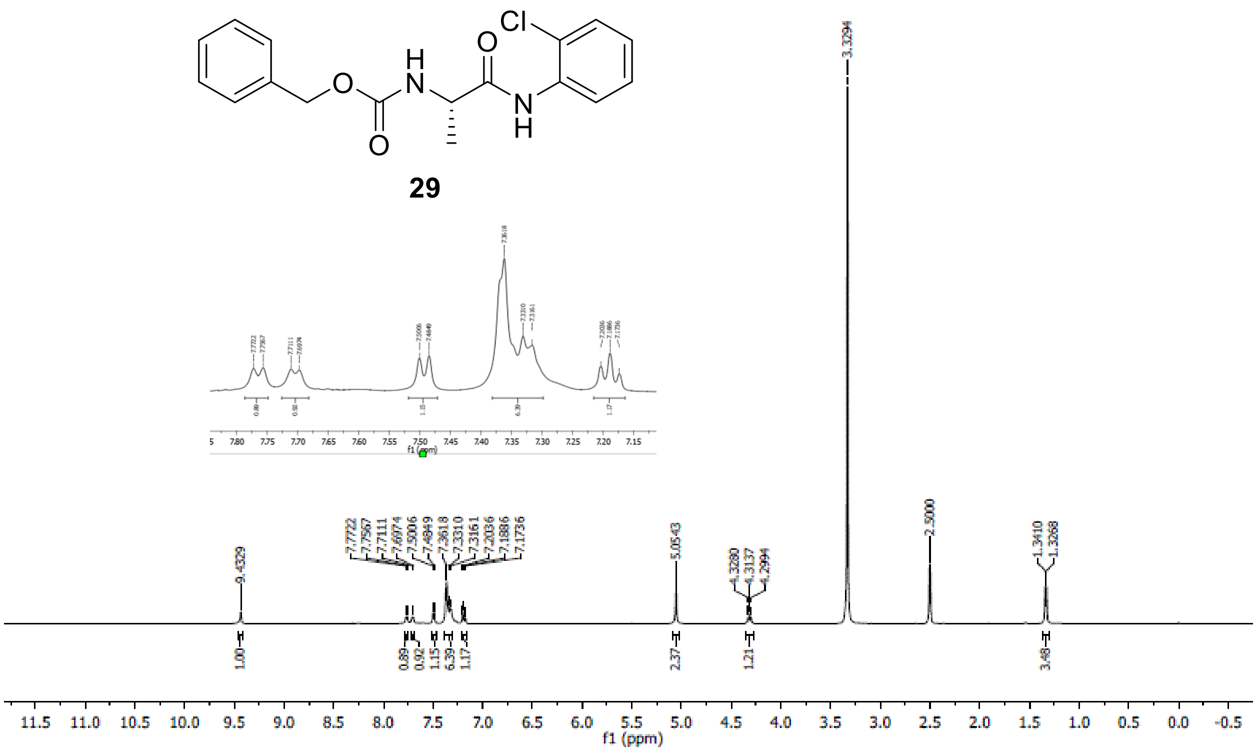

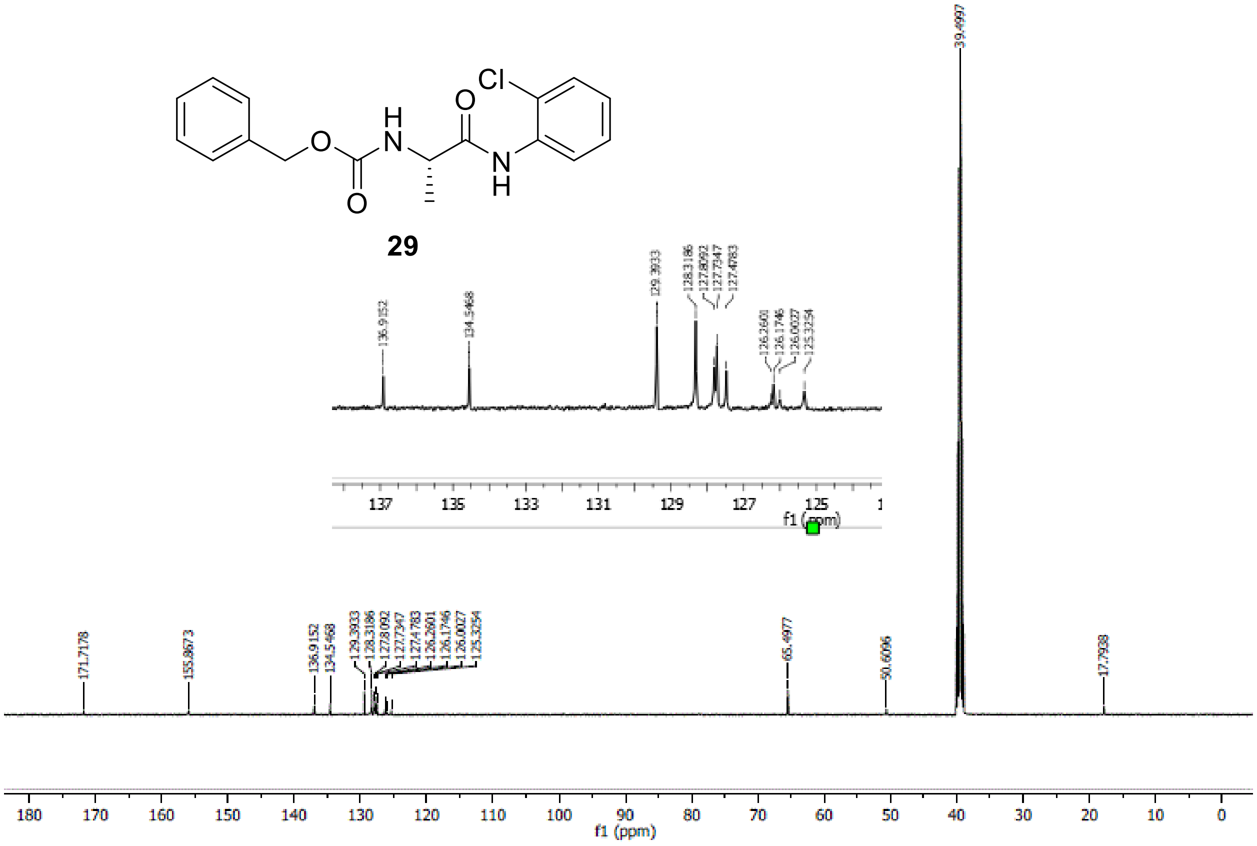

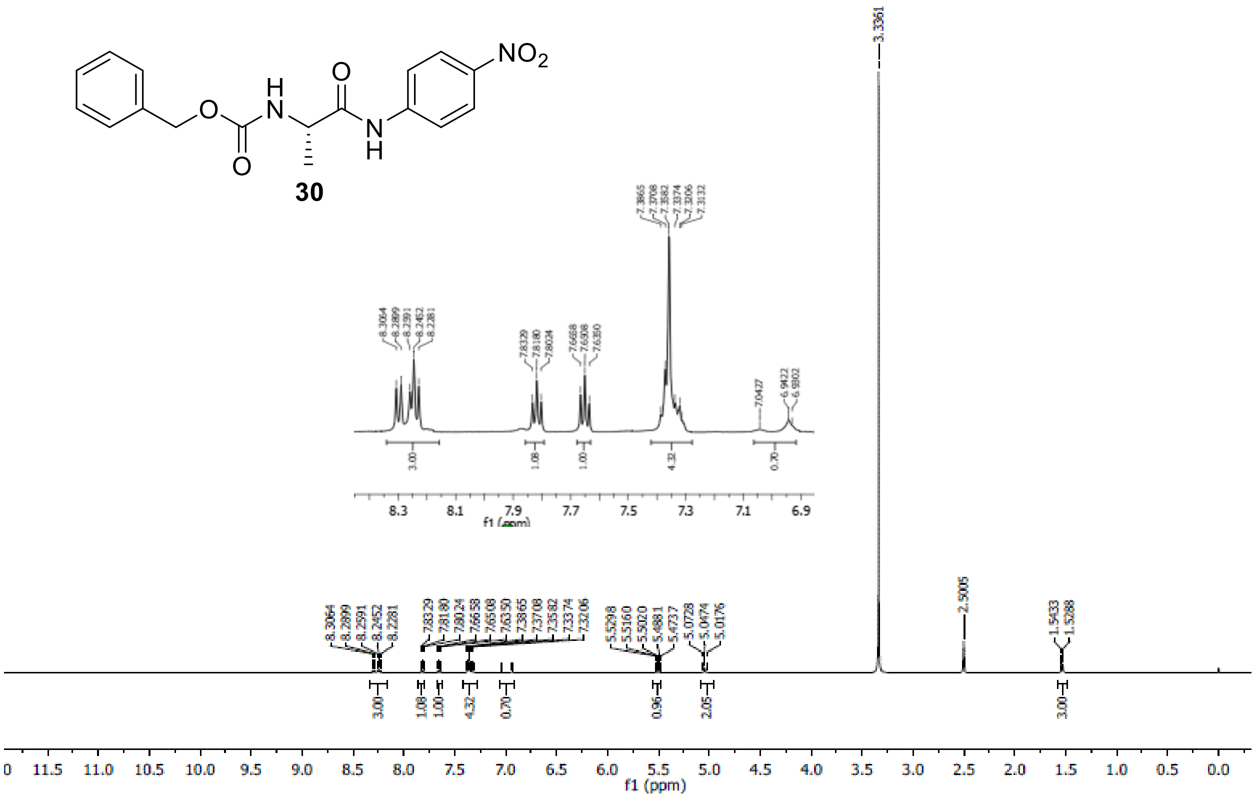

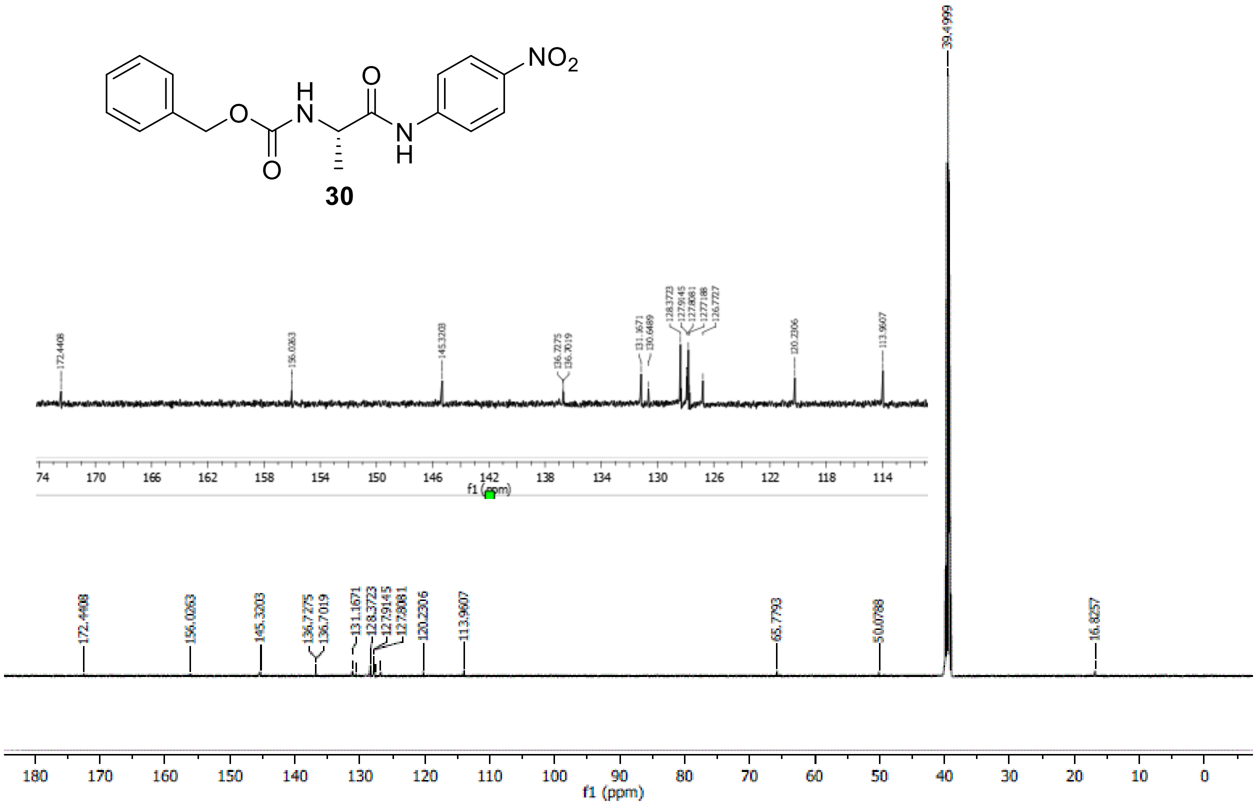

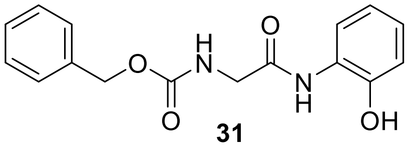

31

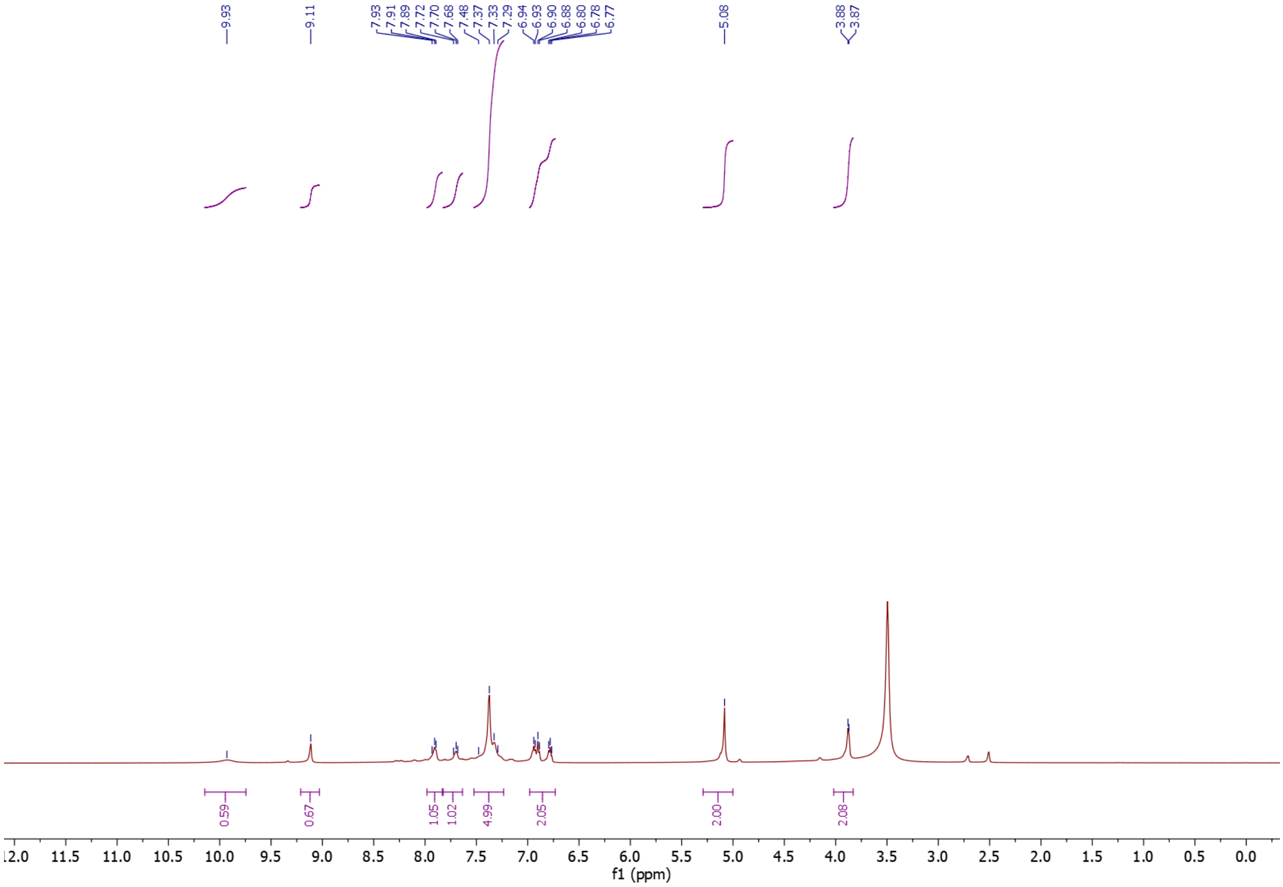
9.93
9.11
7.93
7.91
7.89
7.72
7.70
7.68
7.48
7.37
7.33
7.29
6.94
6.93
6.90
6.88
6.80
6.78
6.77
5.08
3.88
3.87
0.59
0.67
1.05
1.02
4.99
2.05
2.00
2.08
12.0 11.5 11.0 10.5 10.0 9.5 9.0 8.5 8.0 7.5 7.0 6.5 6.0 5.5 5.0 4.5 4.0 3.5 3.0 2.5 2.0 1.5 1.0 0.5 0.0
f1 (ppm)

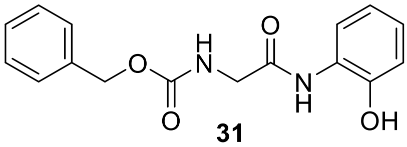
31

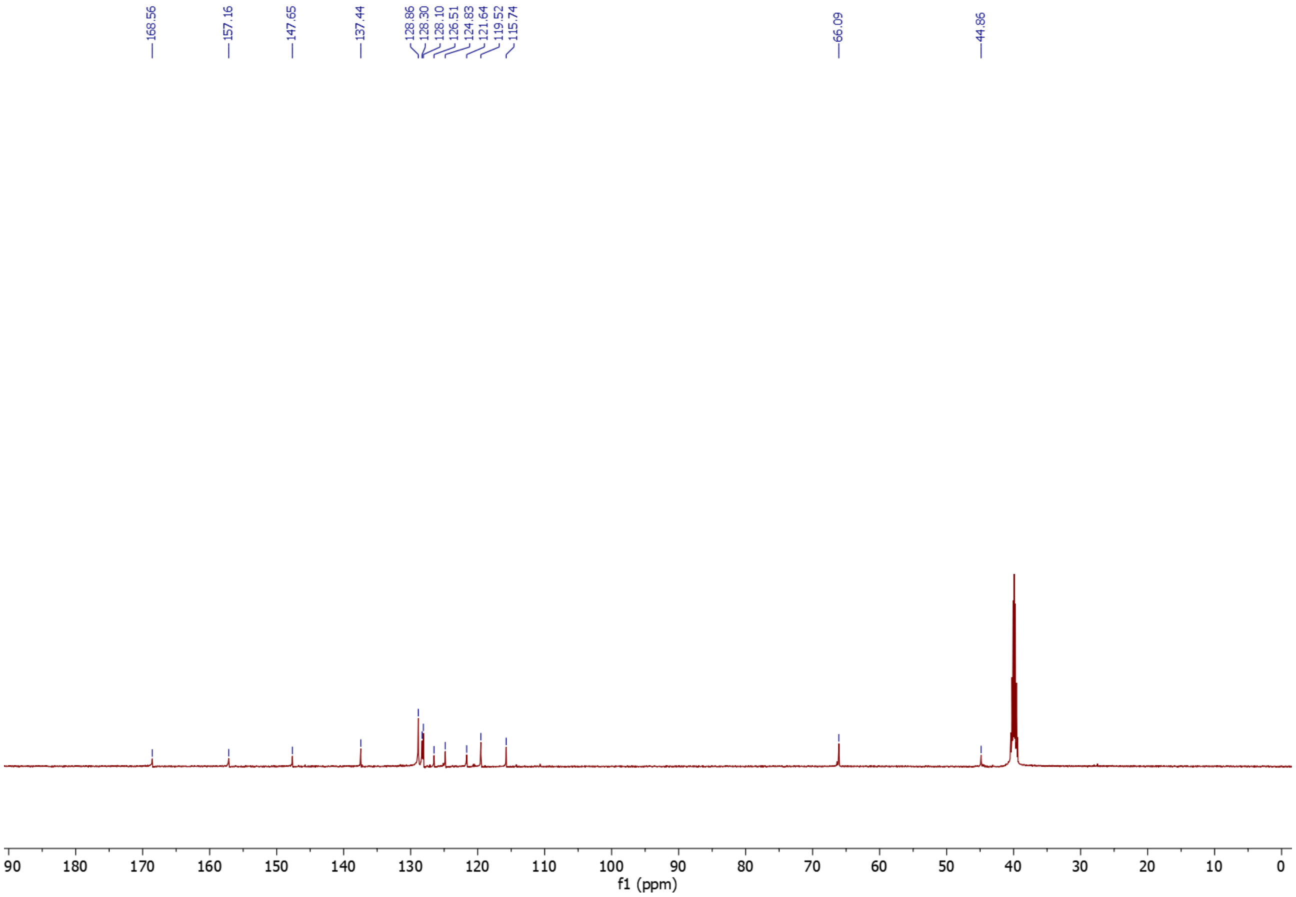
168.56
157.16
147.65
137.44
128.86
128.30
128.10
126.51
124.83
121.64
119.52
115.74
66.09
44.86
f1 (ppm)

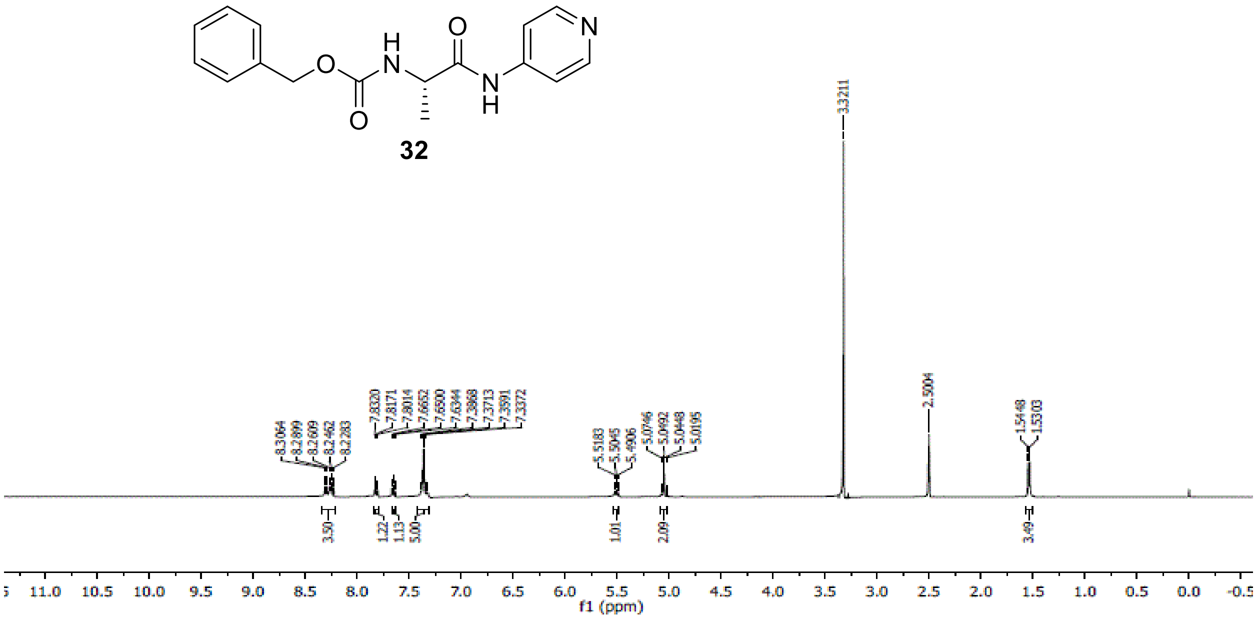
32
f1 (ppm)

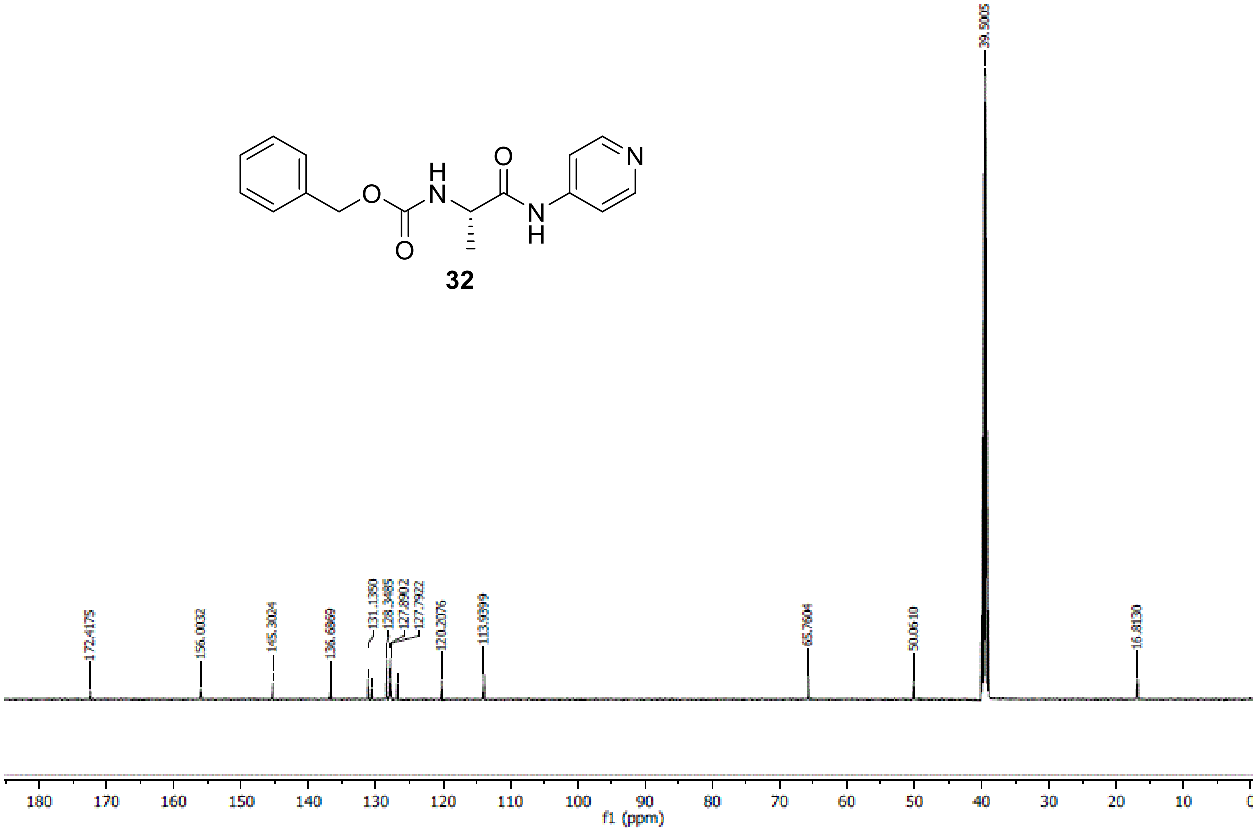
32
f1 (ppm)

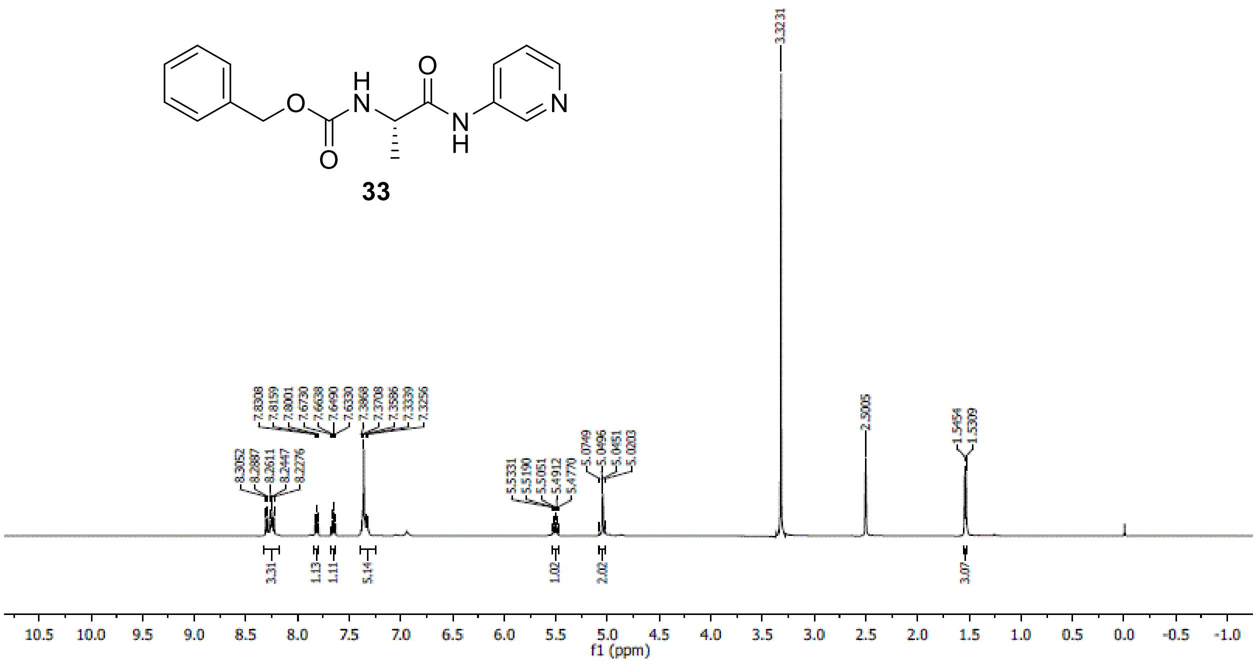
33
f1 (ppm)

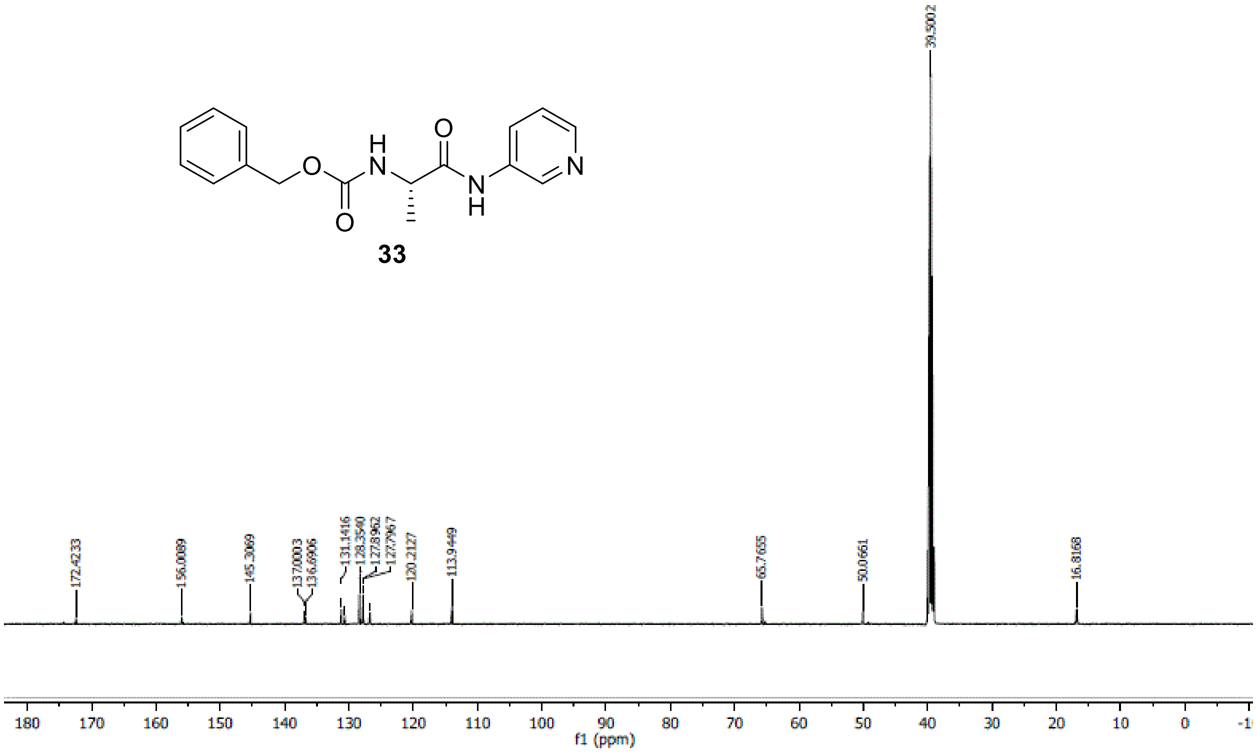
33
f1 (ppm)

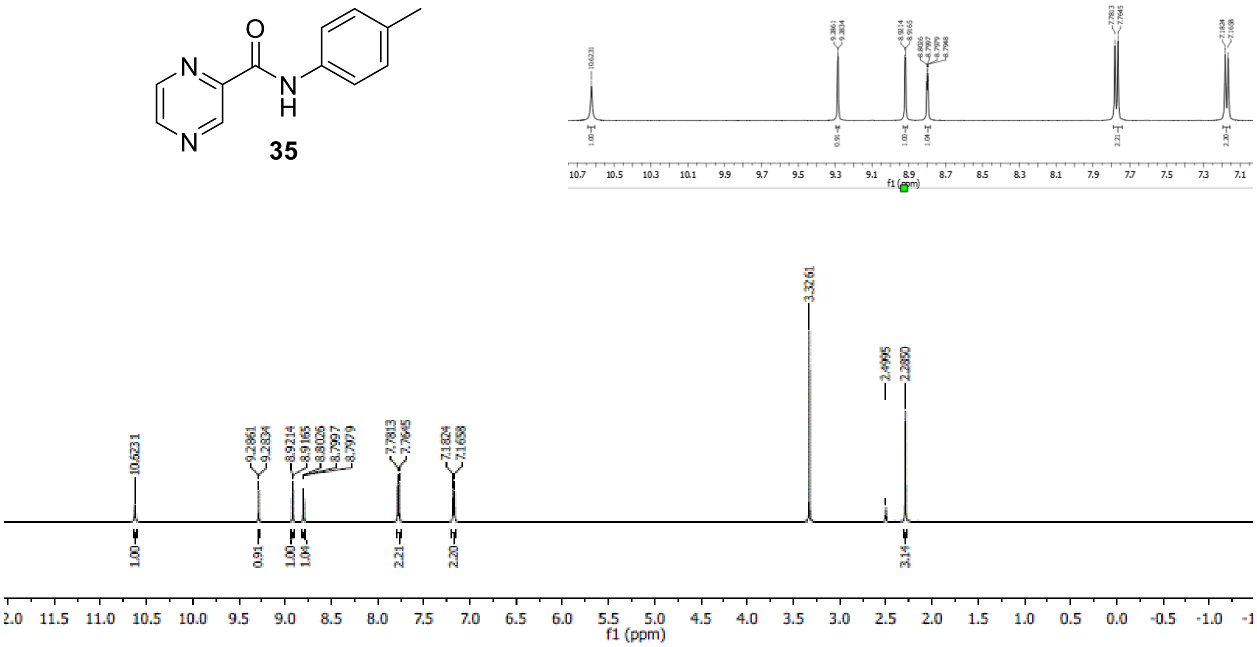
35
10.6231
9.2861
9.2834
8.9214
8.9165
8.8026
8.7997
8.7979
7.7813
7.7645
7.1824
7.1658
3.3261
2.4995
2.2850
1.00
0.91
1.00
1.04
2.21
2.20
3.14
f1 (ppm)

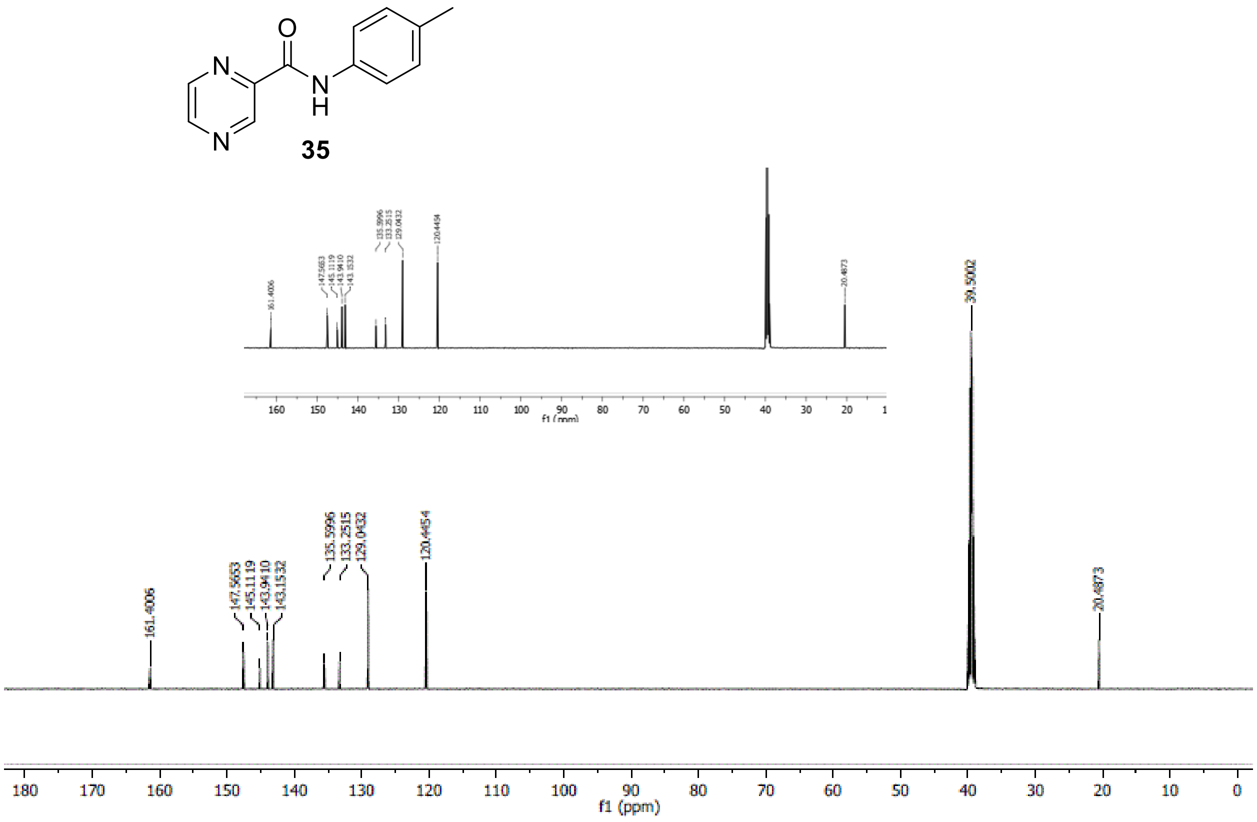
35
161.4006
147.5653
145.1119
143.9410
143.1532
135.5996
133.2515
129.0432
120.4454
39.5002
20.4873
f1 (ppm)

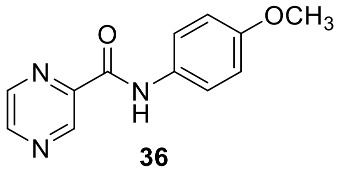
O
N
N
H
N
OCH_3

36

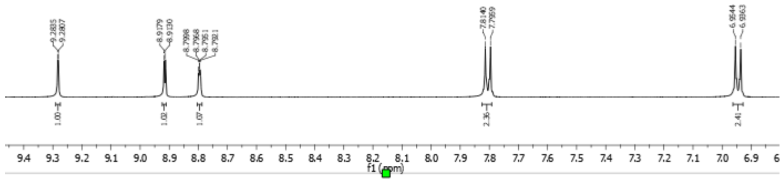
f1 (ppm)

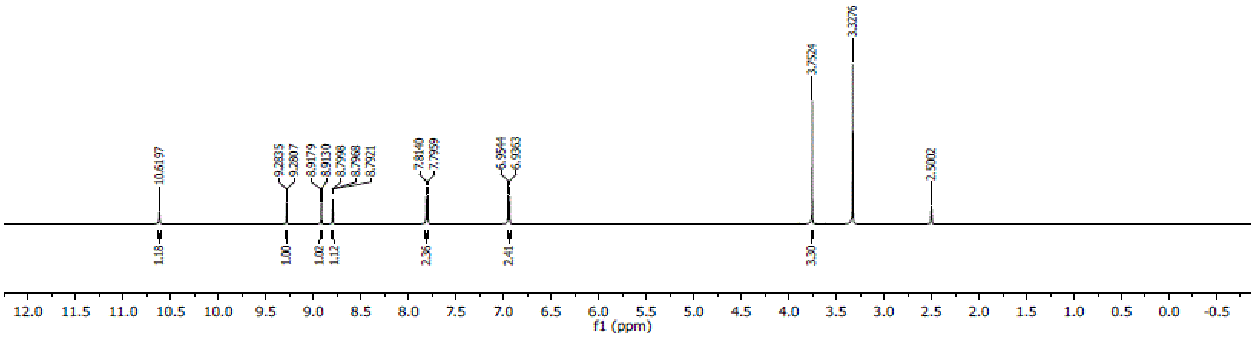
10.6197
9.2835
9.2807
8.9179
8.9130
8.7998
8.7968
8.7921
7.8140
7.7959
6.9544
6.9363
3.7524
3.3276
2.5002
1.18
1.00
1.02
1.12
2.36
2.41
3.30
f1 (ppm)

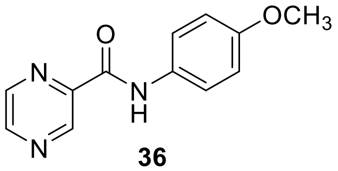
O
N
N
H
N
OCH_3

36

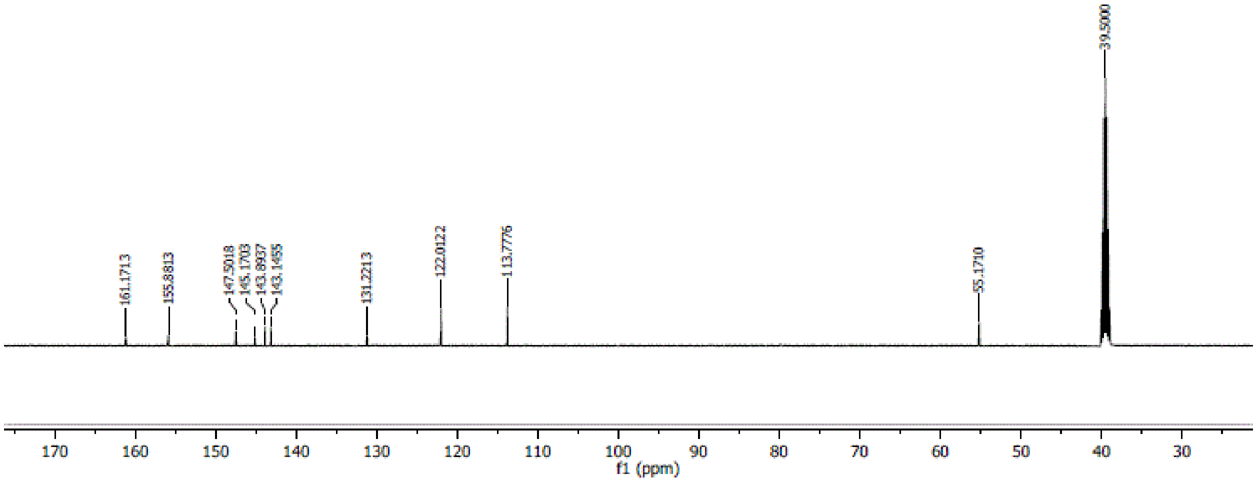
161.1713
155.8813
147.5018
145.1703
143.8937
143.1455
131.2213
122.0122
113.7776
55.1710
39.5000
f1 (ppm)

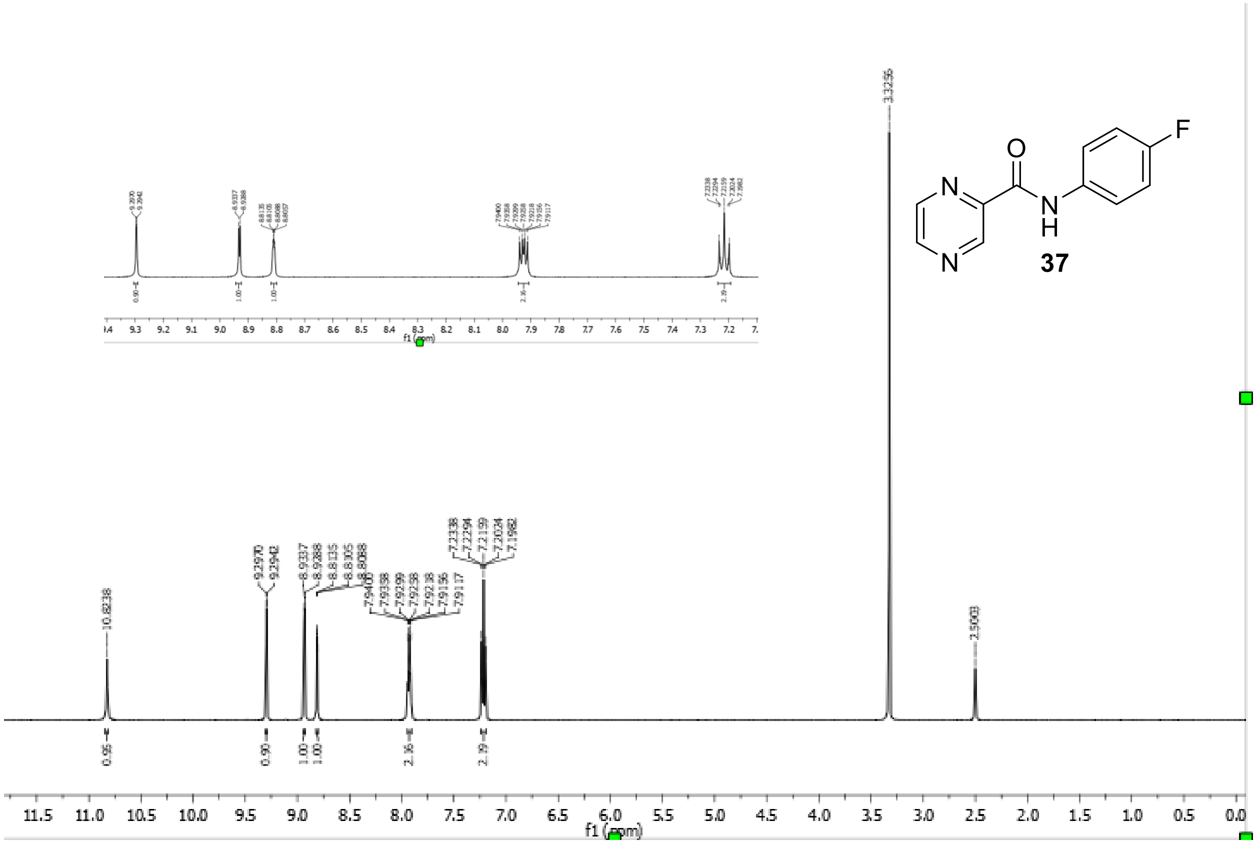

37
10.8238
9.2970
9.2942
8.9337
8.9288
8.8135
8.8105
8.8088
7.9400
7.9358
7.9299
7.9258
7.9218
7.9156
7.9117
7.2338
7.2294
7.2159
7.2024
7.1982
3.3256
2.5003
0.95
0.90
1.00
1.00
2.16
2.19
f1 (ppm)

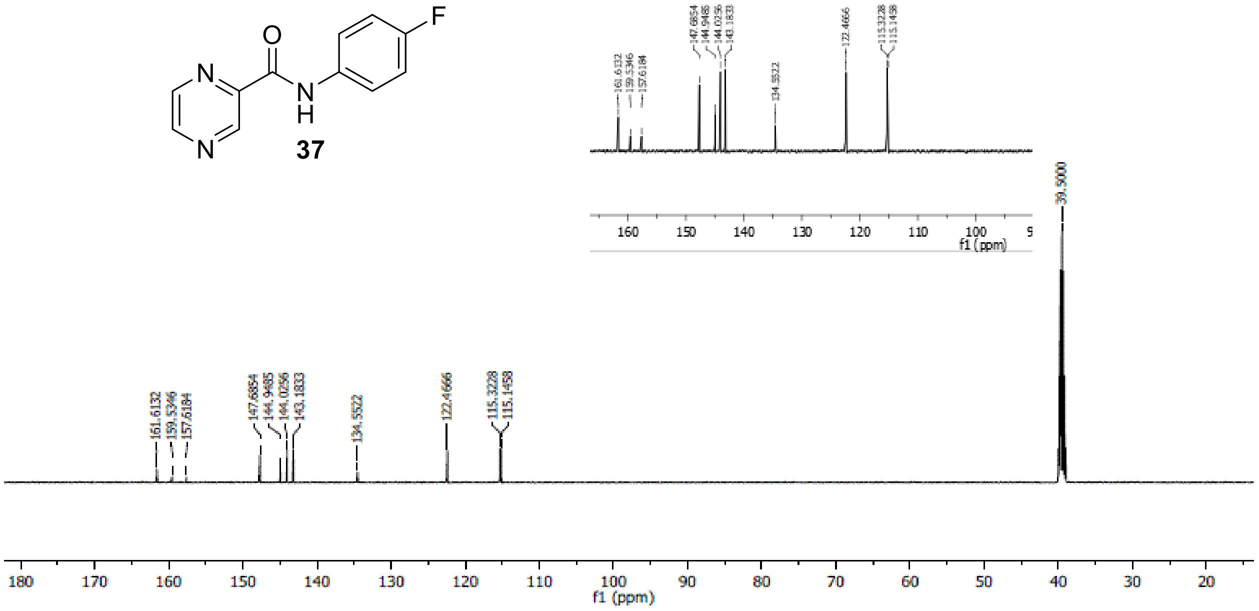

37
161.6132
159.5346
157.6184
147.6854
144.9485
144.0256
143.1833
134.5522
122.4666
115.3228
115.1458
39.5000
f1 (ppm)

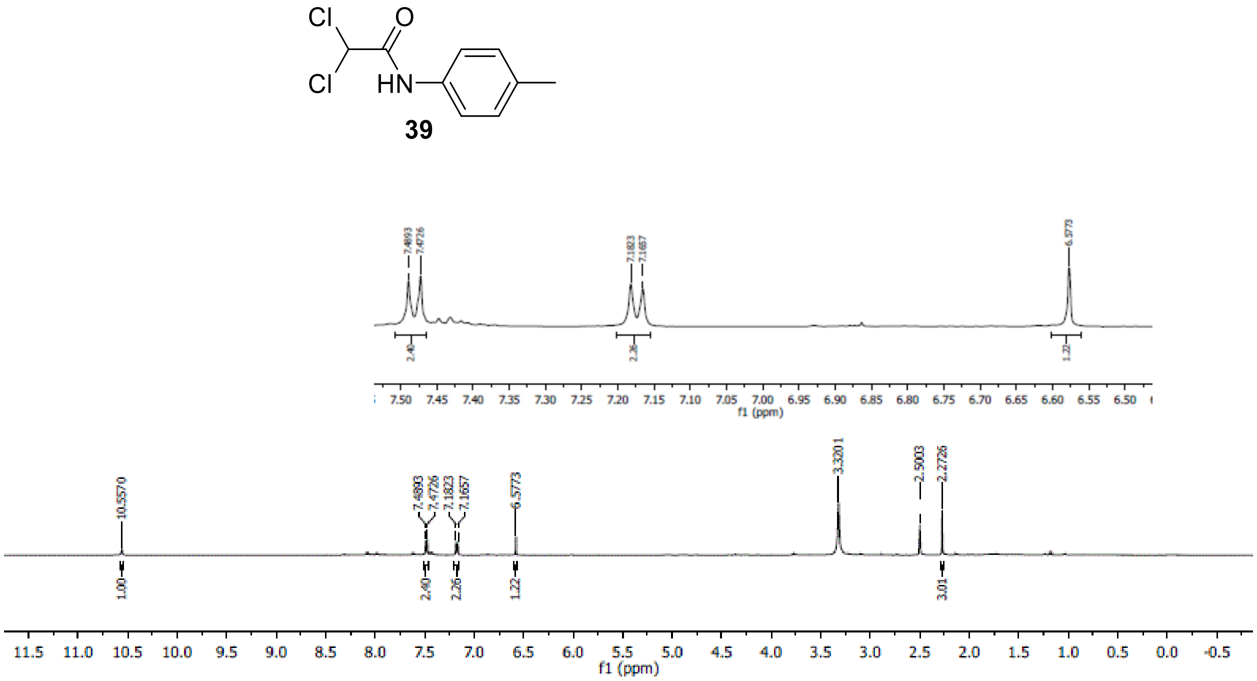
Cl
O
Cl
HN
39
7.4893
7.4726
7.1823
7.1657
6.5773
2.40
2.26
1.22
f1 (ppm)
10.5570
3.3201
2.5003
2.2726
1.00
3.01
f1 (ppm)

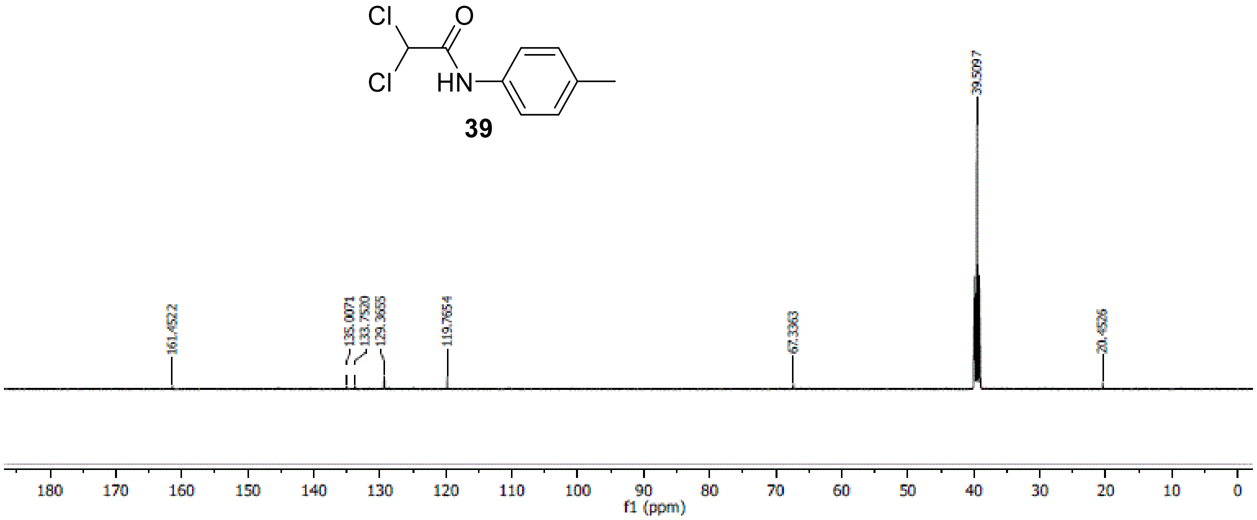
Cl
O
Cl
HN
39
39.5097
161.4522
135.0071
133.7520
129.3655
119.7654
67.3363
20.4526
f1 (ppm)

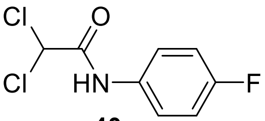

40

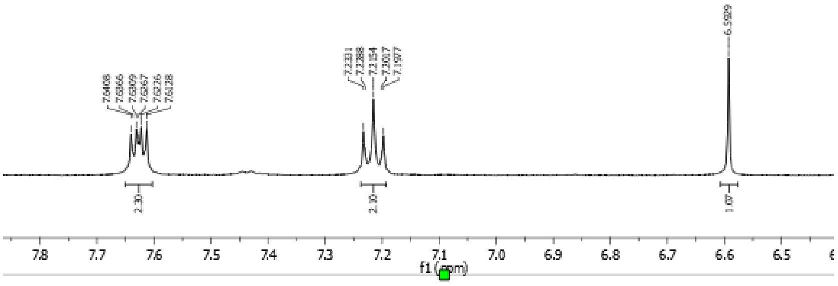

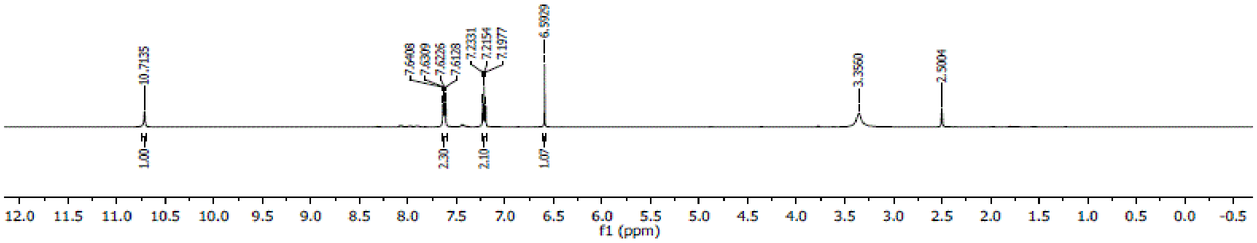

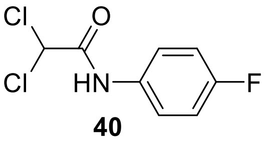

40

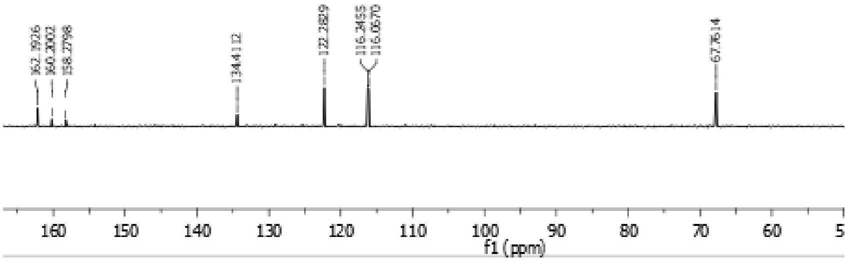

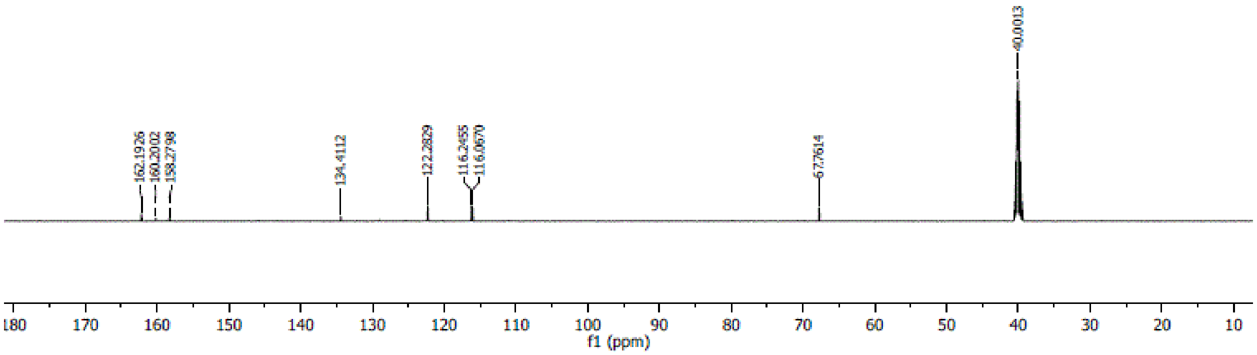

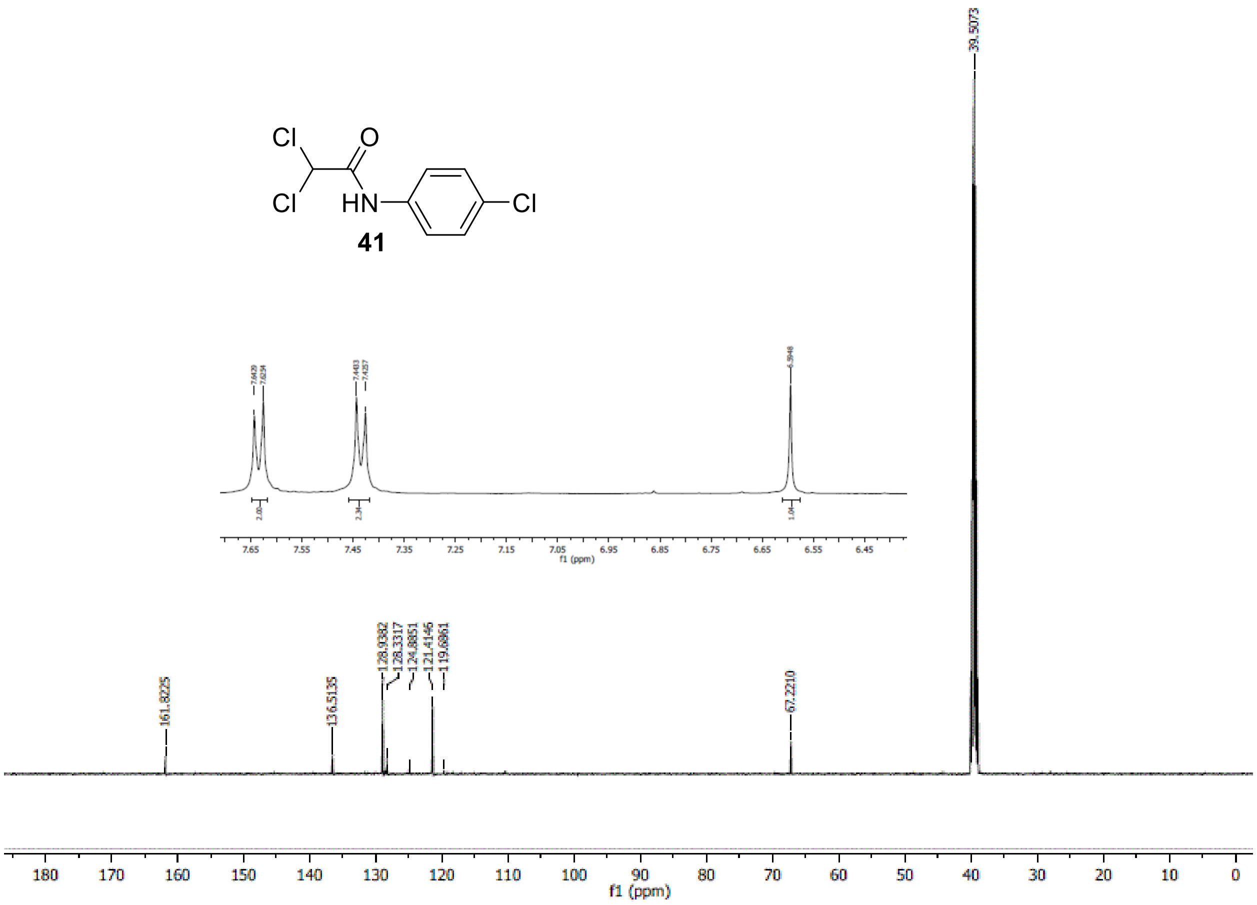

Cl
O
Cl
HN
Cl
41
7.6429
7.6254
7.4433
7.4257
6.5948
2.00
2.34
1.04
f1 (ppm)
161.8225
136.5135
128.9382
128.3317
124.8851
121.4146
119.6861
67.2210
39.5073
180 170 160 150 140 130 120 110 100 90 80 70 60 50 40 30 20 10 0
f1 (ppm)

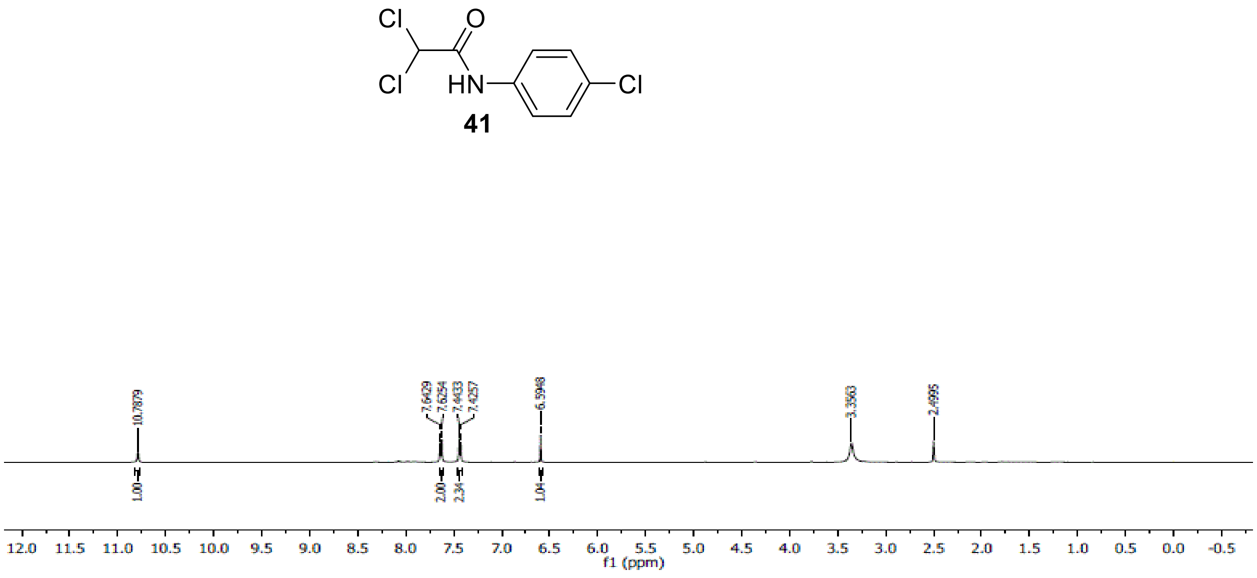

Cl
O
Cl
HN
Cl
41
10.7879
7.6429
7.6254
7.4433
7.4257
6.5948
3.3563
2.4995
1.00
2.00
2.34
1.04
12.0 11.5 11.0 10.5 10.0 9.5 9.0 8.5 8.0 7.5 7.0 6.5 6.0 5.5 5.0 4.5 4.0 3.5 3.0 2.5 2.0 1.5 1.0 0.5 0.0 -0.5
f1 (ppm)

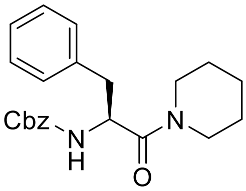

48

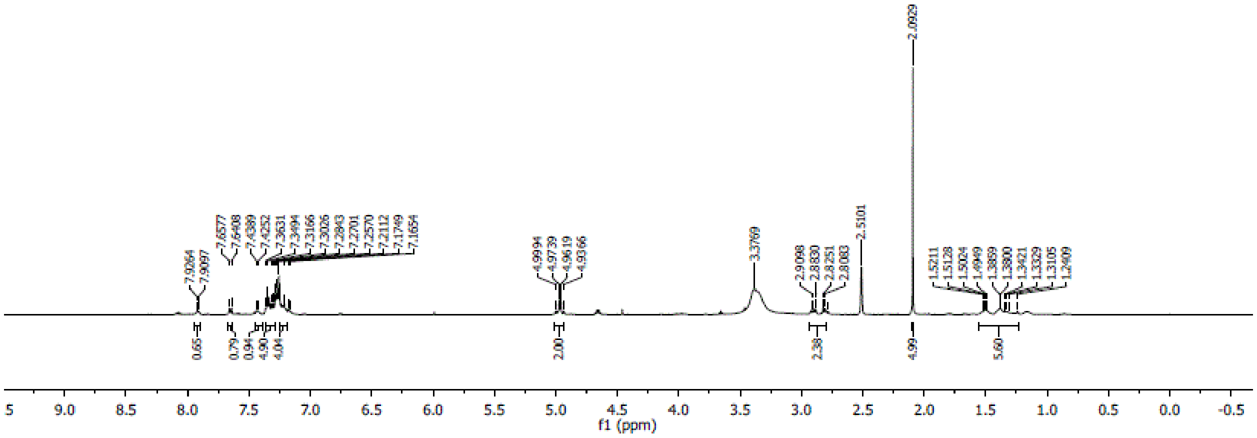

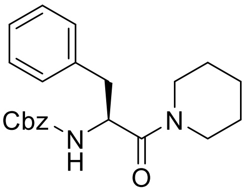

48

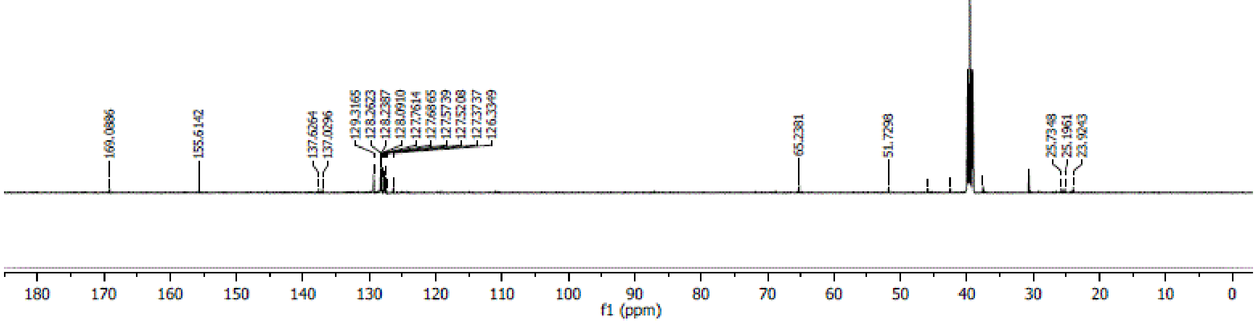

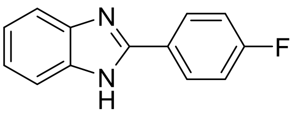

53

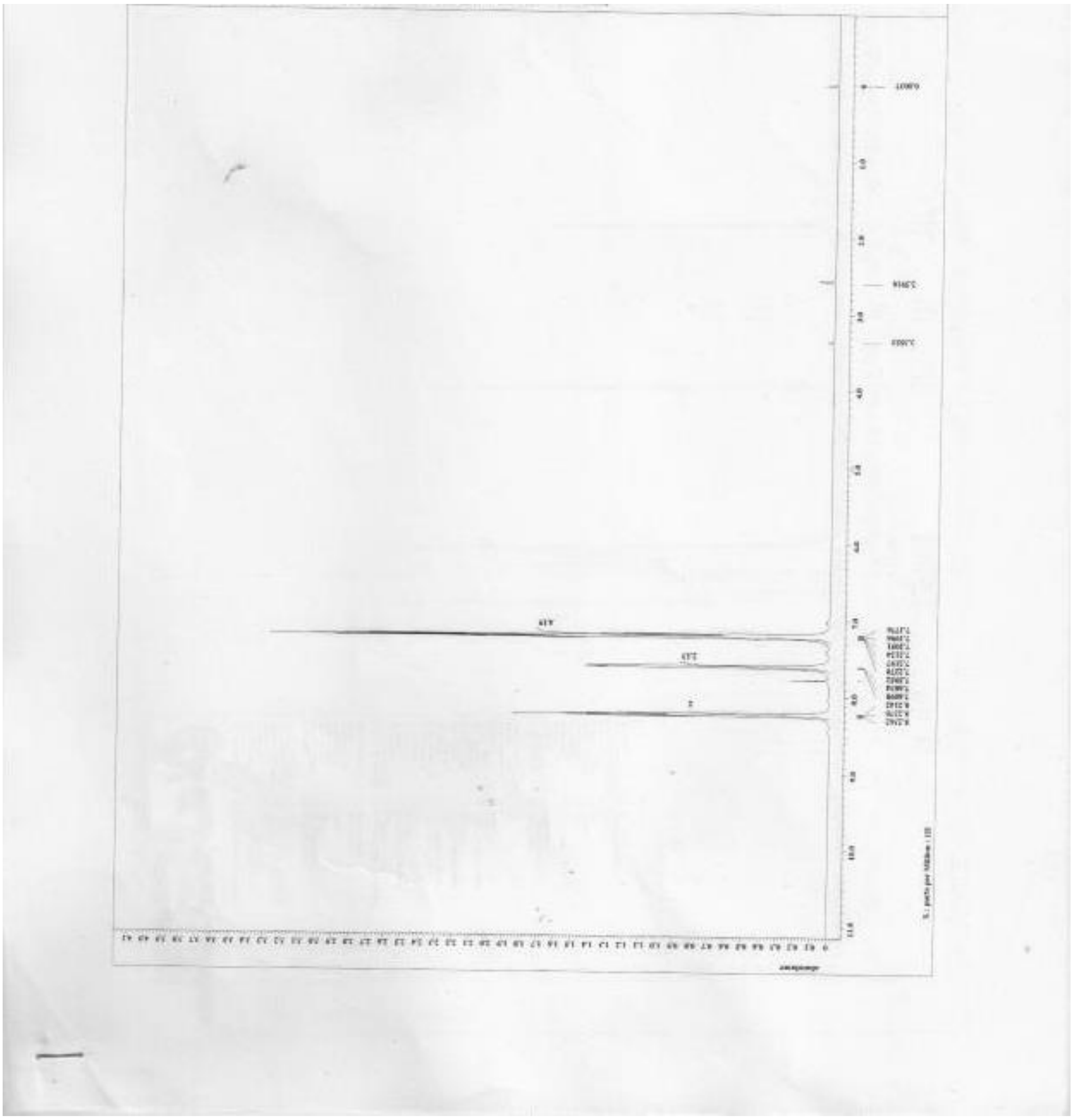

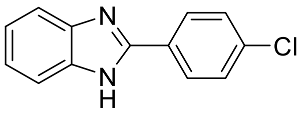

54

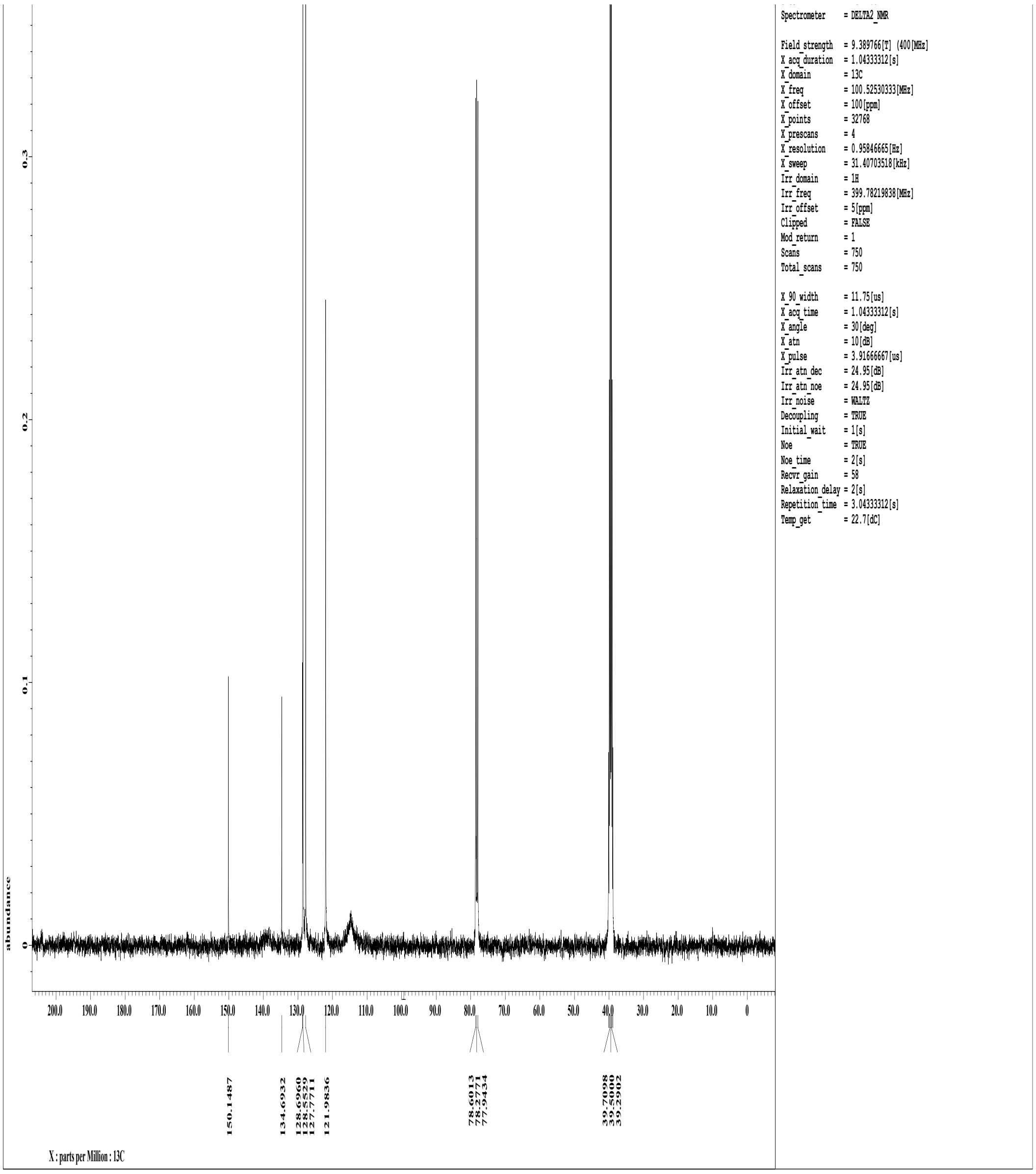
Spectrometer = DELTA2_NMR
Field_strength = 9.389766[T] (400[MHz]
X_acq_duration = 1.04333312[s]
X_domain = 13C
X_freq = 100.52530333[MHz]
X_offset = 100[ppm]
X_points = 32768
X_prescans = 4
X_resolution = 0.95846665[Hz]
X_sweep = 31.40703518[kHz]
Irr_domain = 1H
Irr_freq = 399.78219838[MHz]
Irr_offset = 5[ppm]
Clipped = FALSE
Mod_return = 1
Scans = 750
Total_scans = 750
X_90_width = 11.75[us]
X_acq_time = 1.04333312[s]
X_angle = 30[deg]
X_atn = 10[dB]
X_pulse = 3.91666667[us]
Irr_atn_dec = 24.95[dB]
Irr_atn_noe = 24.95[dB]
Irr_noise = WALTZ
Decoupling = TRUE
Initial_wait = 1[s]
Noe = TRUE
Noe_time = 2[s]
Recvr_gain = 58
Relaxation_delay = 2[s]
Repetition_time = 3.04333312[s]
Temp_get = 22.7[dC]
abundance
0.3
0.2
0.1
0
150.1487
134.6932
128.6960
128.5529
127.5721
121.9836
78.6013
78.2771
77.9434
39.7098
39.5000
39.2902
X : parts per Million : 13C

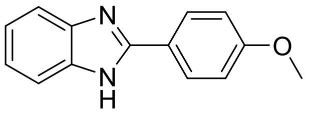
N
N
H
O

59

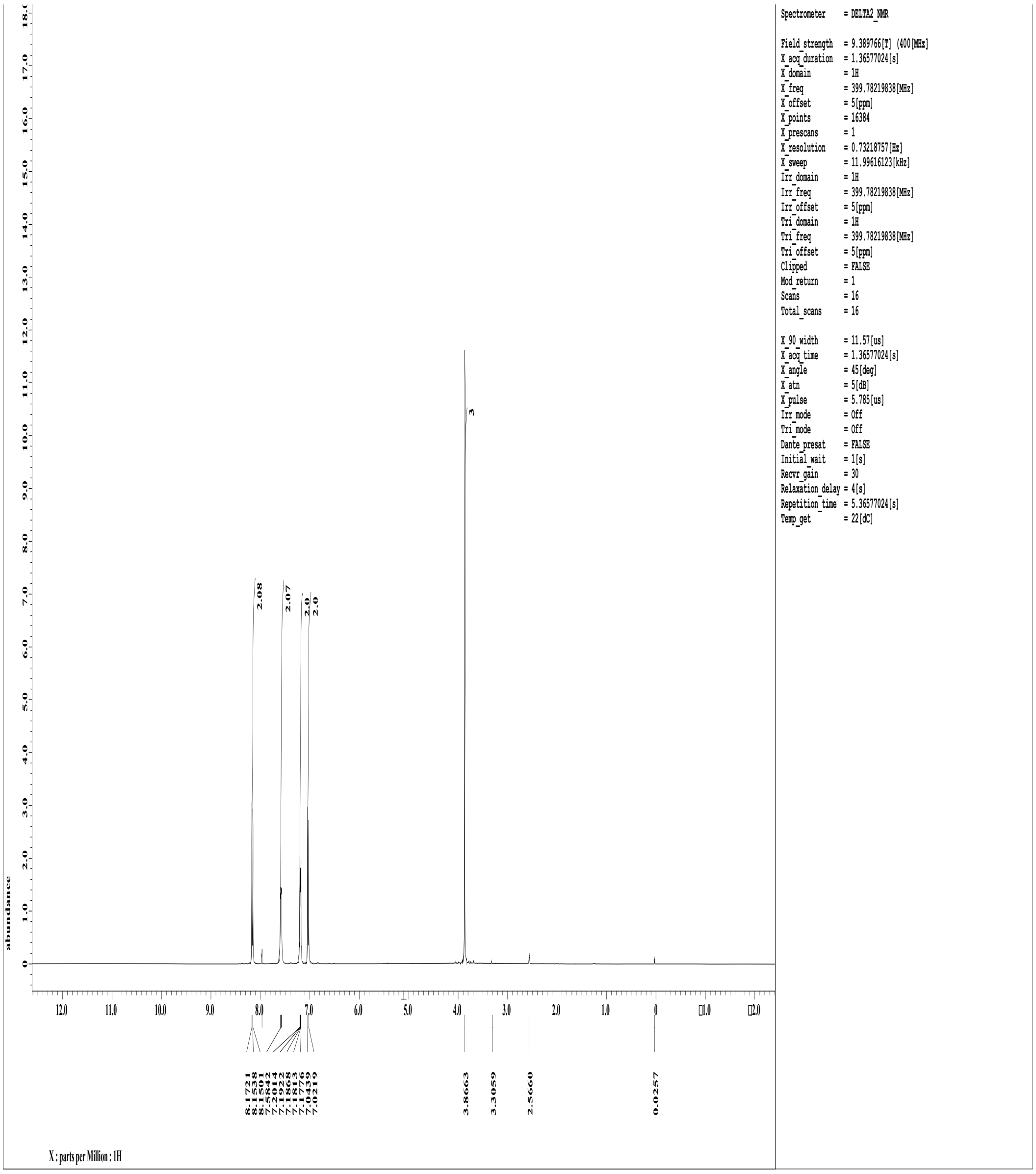
abundance
X : parts per Million : 1H
2.08
2.07
2.0
2.0
3
8.1721
8.1538
8.1501
7.5842
7.5814
7.1922
7.1868
7.1813
7.1776
7.0439
7.0219
3.8663
3.3059
2.5660
0.0257
Spectrometer = DELTA2_NMR
Field_strength = 9.389766[T] (400[MHz]
X_acq_duration = 1.36577024[s]
X_domain = 1H
X_freq = 399.78219838[MHz]
X_offset = 5[ppm]
X_points = 16384
X_prescans = 1
X_resolution = 0.73218757[Hz]
X_sweep = 11.99616123[kHz]
Irr_domain = 1H
Irr_freq = 399.78219838[MHz]
Irr_offset = 5[ppm]
Tri_domain = 1H
Tri_freq = 399.78219838[MHz]
Tri_offset = 5[ppm]
Clipped = FALSE
Mod_return = 1
Scans = 16
Total_scans = 16
X_90_width = 11.57[us]
X_acq_time = 1.36577024[s]
X_angle = 45[deg]
X_atn = 5[dB]
X_pulse = 5.785[us]
Irr_mode = Off
Tri_mode = Off
Dante_presat = FALSE
Initial_wait = 1[s]
Recvr_gain = 30
Relaxation_delay = 4[s]
Repetition_time = 5.36577024[s]
Temp_get = 22[dC]

HPLC spectra of compound **9** and **9a**

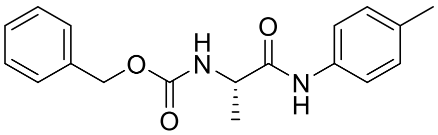

9

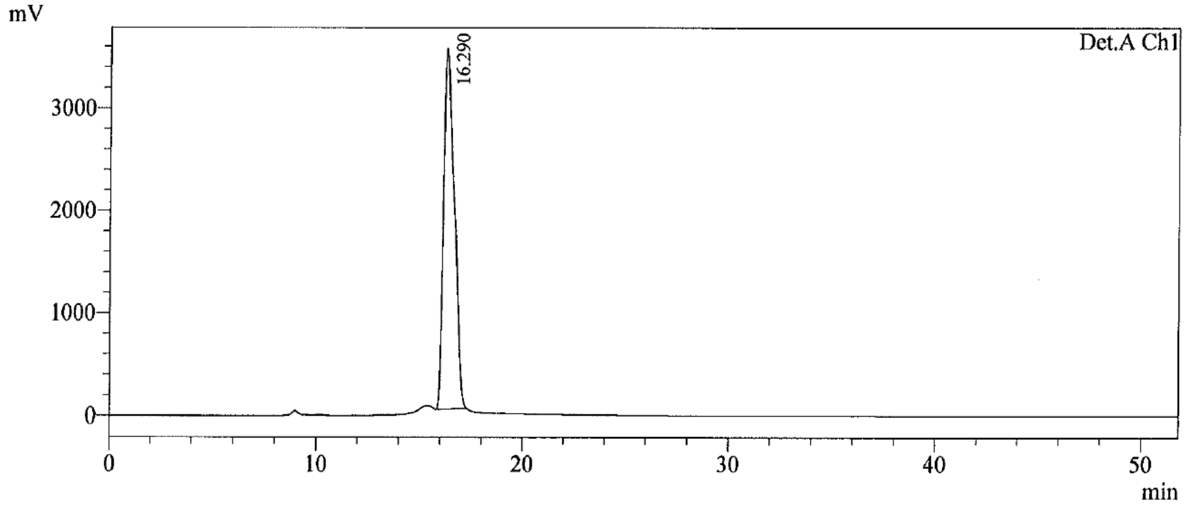

1 Det.A Ch1 / 254nm

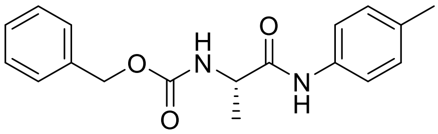

9

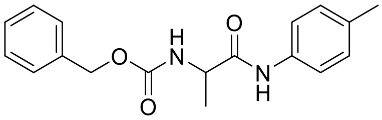

9+9'

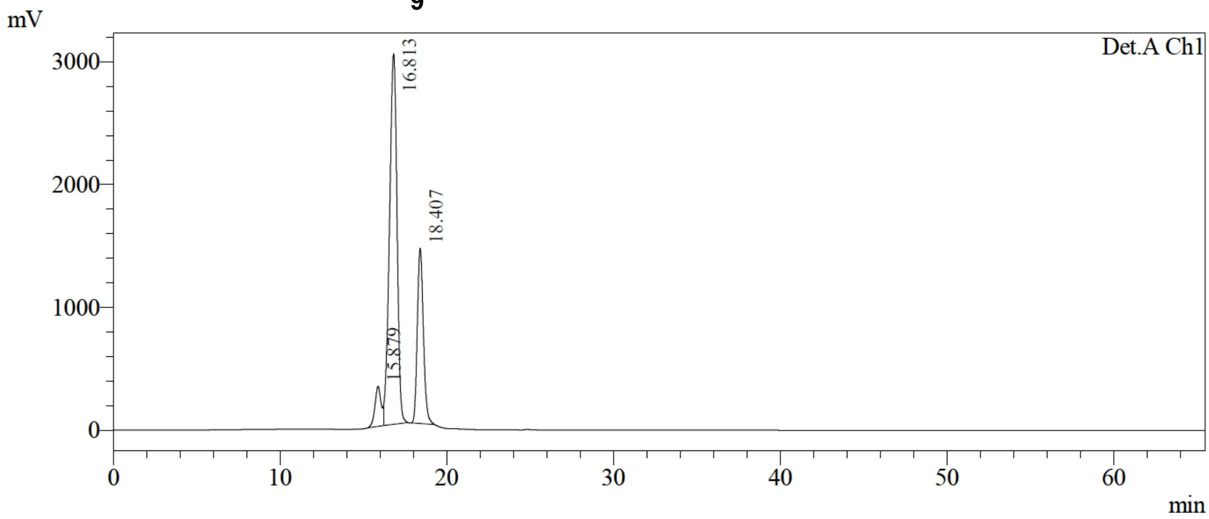

1 Det.A Ch1 / 254nm
